# Capturing the Biofuel Wellhead and Powerhouse: The Chloroplast and Mitochondrial Genomes of the Leguminous Feedstock Tree *Pongamia pinnata*

**DOI:** 10.1371/journal.pone.0051687

**Published:** 2012-12-14

**Authors:** Stephen H. Kazakoff, Michael Imelfort, David Edwards, Jasper Koehorst, Bandana Biswas, Jacqueline Batley, Paul T. Scott, Peter M. Gresshoff

**Affiliations:** 1 Australian Research Council Centre of Excellence for Integrative Legume Research, The University of Queensland, Brisbane, Queensland, Australia; 2 Advanced Water Management Centre, The University of Queensland, Brisbane, Queensland, Australia; 3 Australian Centre for Plant Functional Genomics, The University of Queensland, Brisbane, Queensland, Australia; J. Craig Venter Institute, United States of America

## Abstract

*Pongamia pinnata* (syn. *Millettia pinnata*) is a novel, fast-growing arboreal legume that bears prolific quantities of oil-rich seeds suitable for the production of biodiesel and aviation biofuel. Here, we have used Illumina® ‘Second Generation DNA Sequencing (2GS)’ and a new short-read *de novo* assembler, SaSSY, to assemble and annotate the Pongamia chloroplast (152,968 bp; cpDNA) and mitochondrial (425,718 bp; mtDNA) genomes. We also show that SaSSY can be used to accurately assemble 2GS data, by re-assembling the *Lotus japonicus* cpDNA and in the process assemble its mtDNA (380,861 bp). The Pongamia cpDNA contains 77 unique protein-coding genes and is almost 60% gene-dense. It contains a 50 kb inversion common to other legumes, as well as a novel 6.5 kb inversion that is responsible for the non-disruptive, re-orientation of five protein-coding genes. Additionally, two copies of an inverted repeat firmly place the species outside the subclade of the Fabaceae lacking the inverted repeat. The Pongamia and *L. japonicus* mtDNA contain just 33 and 31 unique protein-coding genes, respectively, and like other angiosperm mtDNA, have expanded intergenic and multiple repeat regions. Through comparative analysis with *Vigna radiata* we measured the average synonymous and non-synonymous divergence of all three legume mitochondrial (1.59% and 2.40%, respectively) and chloroplast (8.37% and 8.99%, respectively) protein-coding genes. Finally, we explored the relatedness of Pongamia within the Fabaceae and showed the utility of the organellar genome sequences by mapping transcriptomic data to identify up- and down-regulated stress-responsive gene candidates and confirm *in silico* predicted RNA editing sites.

## Introduction

In view of diminishing liquid fossil fuel reserves, the large-scale supply of plant-derived oil for sustainable biofuel production is an important challenge for translational biotechnology. *Pongamia pinnata* (also called *Millettia pinnata*), is an undomesticated arboreal legume that can provide biofuel feedstock through high productivity of oil-rich seeds [1]. Its drought- and salinity-tolerant capabilities, free it from concerns regarding the use of land, labour and water [2]. However, it is the low nitrogen input requirements that really give Pongamia an ‘edge’ over other biofuel candidates. As a legume, Pongamia is capable of forming specialized organs, called root nodules, through symbiotic interactions with nitrogen-fixing soil bacteria collectively called Rhizobia. Biological nitrogen fixation, resulting from a functional symbiosis, negates or reduces the need for nitrogen fertilizer application, which is critical for environmentally sustainable plant oil production. With respect to greenhouse gas emissions, all steps in the production, transport and application of nitrogen fertilizers are costly, can cause significant environmental pollution through run-off and seepage, and are highly dependent on fossil fuels. For Pongamia to become an industrial-scale feedstock for the emerging biodiesel and aviation biofuel industries, biotechnologies will be needed to advance selection and breeding ahead of the impending peak oil crisis.

Central to the domestication of Pongamia is an understanding of its molecular genetics, and this includes aspects of gene content and structural organization in relation to other plant genomes [3]. Here, we have taken a crucial step forward in the development pathway of this biofuel tree by using Illumina® Second Generation Sequencing (2GS) and SaSSY bioinformatics software [4] to sequence and assemble its chloroplast (cpDNA) and mitochondrial (mtDNA) genomes. At a fraction of the cost of whole nuclear genome sequencing, the Pongamia organellar genomes not only provide valuable insight into the evolution of a largely unexplored species, but will also greatly assist and accelerate the process of germplasm improvement through breeding and molecular manipulation. For example, these organelles have prokaryotic ancestral origins as well as a functional and phylogenetic relationship to the evolution of their nuclear genomes. They also undergo specific molecular events (e.g. post-transcriptional RNA editing, codon usage patterns) and, due to their high level of conservation and maternal inheritance, can be used to develop population markers for phylogenetic and phylogeographic studies. This uniparental mode of inheritance also makes plastid genomes excellent targets for genetic transformation; this popular field of research is called transplastomics. Plastid transformation has various advantages over nuclear gene transformation, including increased levels of transgene expression, the ability to co-express multiple genes and transgene containment because plastid DNA, in most plant species, is not transmitted through pollen [5]. Thus in order to advance the selection and breeding of elite Pongamia germplasm through the development of a plastid engineering protocol, obtaining whole organellar genome sequence is a decisive first step.

The cpDNA of most flowering plants are circular molecules that are highly conserved in terms of gene content, size and structural organization. These genomes are each approximately 150 kb, often with a quadripartite structure that includes a large single copy region (LSC) and a small single copy region (SSC) separated by a pair of large, approximately 25 kb, inverted repeats (IRA and IRB). Since the publication of the first cpDNA, belonging to *L. japonicus* [6], it has become increasingly evident that the Fabaceae consist of two contrasting patterns of cpDNA evolution. Temperate legumes, such as pea and clover, lack one copy of the large inverted repeat that is otherwise almost universally present in angiosperm cpDNA. The clade that contains both copies of the inverted repeat includes tropical legumes, such as *G. max*, Pongamia and *L. japonicus*, and these share a highly conserved sequence order that differs by a single, 50 kb, inversion from the arrangement typical of most vascular plants [7]. Interestingly, the structural differences between the cpDNA of the two legume clades also correspond to two contrasting nodulation types [8]. The tropical legumes predominately form determinate nodules, which lack meristematic activity (hence their spherical shape), whereas the temperate legume species all give rise to indeterminate nodules, which have a cylindrical appearance and persistent meristem. The cpDNA of ten legumes, half each of tropical and temperate species, can now be found on the National Center for Biotechnology Information (NCBI) website (http://www.ncbi.nlm.nih.gov/genomes/GenomesGroup.cgi?opt=plastid&taxid=3814).

Angiosperm mtDNA are dynamic organellar genomes; much unlike their cpDNA counterparts. These mtDNA are the largest, most variable in size (200 to 2,500 kb) and the least gene-dense amongst the eukaryotes because they harbour expanded intergenic regions. They have a multipartite organization that consists of a variety of coexisting subcircles and/or isomeric circles, which when unfolded represent a presumptive “master circle” containing all mitochondrial-derived DNA. These subcircles and isomeric circles are alternate forms of the master circle; generated via intramolecular recombination between sets of large (often greater than 1 kb) direct and inverted repeat sequences, respectively. The master circle may also contain multiple sets of such repeats and these contribute to the genome’s complex structural organization and regulation. Compared with cpDNA, plant mtDNA have limited use in domestication studies because they have a lower (approximately four-fold) mutation rate and therefore evolve more slowly [9]. However, angiosperm mtDNA is often maternally inherited, and this enables the plant’s maternal lineage to be traced far back in time, thus providing a useful phylogenetic tool to evaluate distant evolutionary relationships. Additionally, mitochondria are also viable transformation vessels capable of transgene containment and high levels of gene expression. Recently, the first legume mtDNA belonging to *V. radiata* (mung bean) was published and is now available on the NCBI website (http://www.ncbi.nlm.nih.gov/genomes/GenomesGroup.cgi?opt=organelle&taxid=3814) [10]. This genome, at 401,262 bp, has a similar size to other seed plant mtDNA; however it lacks the large repeats that other angiosperms use for frequent homologous recombination.

In this study, we broaden the knowledge base of legume mtDNA three-fold and report the first organellar genomes of an arboreal legume. Our analysis and genomic comparison with the *L. japonicus* and *V. radiata* organellar genomes, including the *V. radiata* cpDNA [11], reveals structural and functional information that will provide a genetic basis for further domestication of Pongamia as a biofuel feedstock through selection, breeding and molecular improvement. Furthermore, our study exemplifies the use of 2GS and new assembly tools to allow rapid insight into the organellar genomes of previously unexplored species.

## Results

### Organization and Structure of the Pongamia Chloroplast Genome

**Figure 1:**
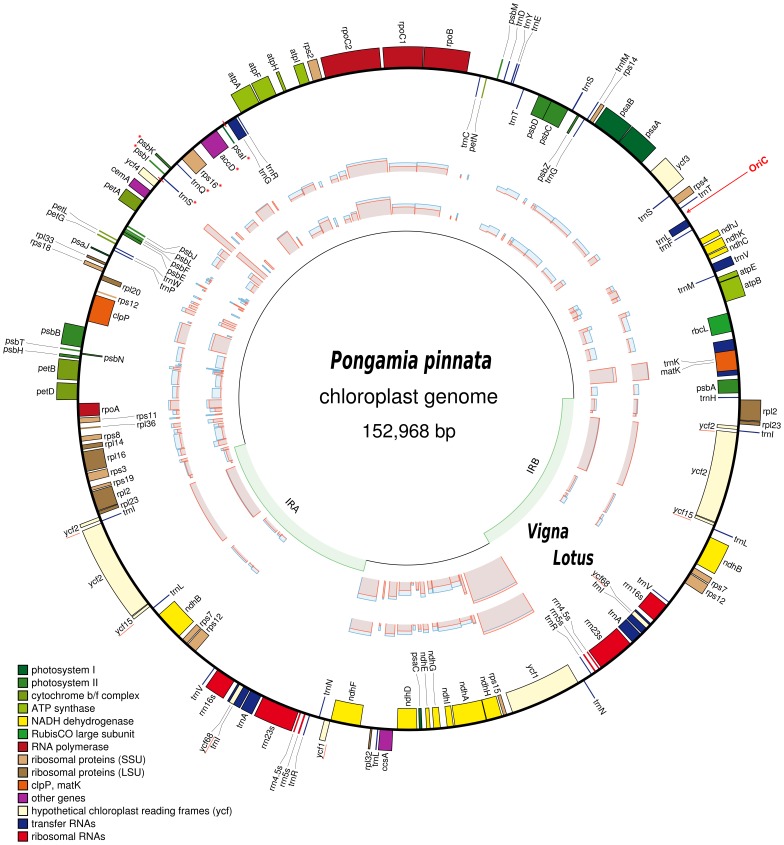
Map of the Pongamia cpDNA (152,968 bp). The innermost circle indicates the locations of the IRs (IRA and IRB, 25,528 bp), which separate the LSC (83,401 bp) and SSC (18,511 bp) regions. Genes on the outermost of the map are transcribed in a counter-clockwise direction and those on the inside clockwise. The graphs plotted between these circles represent the percentage synonymous (blue) and non-synonymous (red) divergence of each protein-coding gene, between Pongamia and *V. radiata*, and, Pongamia and *L. japonicus*. The red arrow indicates the position of the replication origin, *oriC*. Genes marked with a red asterisk are genes in the Pongamia unique inversion event (the bounds of this region have been marked out in red). Genes underlined in red are pseudogenes.

The Pongamia cpDNA (Fig. 1) has a length of 152,968 bp; repeats IRA and IRB of 25,528 bp, a LSC region of 83,401 bp and a SSC region of 18,511 bp. This genome encodes 135 genic features, of which 94 are single copy genes and 16 are duplicated genes on the inverted repeats. The remaining nine features are comprised of two copies of the *ycf2*, *ycf15* and *ycf68* pseudogenes (a copy of each pseudogene appears on both IRA and IRB), two copies of an IR-spanning trans-spliced gene, *rps12*, and a partial copy of the *ycf1* gene present as a pseudogene on the end of IRA (the intact *ycf1* gene spans the junction between the IRB and SSC). We noted four distinct rRNA genes, 30 distinct tRNA genes and 77 distinct protein-coding genes. Four genes, *infA*, *sprA*, *rpl21* and *rpl22*, are commonly lost from angiosperm cpDNA and these were notably absent from the Pongamia cpDNA. When the second inverted repeat is taken into consideration, a further six protein-coding genes, four rRNA and seven tRNA genes can be added to the list. This gives a total of eight rRNA genes, 37 tRNA genes and 83 protein coding genes. The genome thus consists of 51.1% protein-coding, 5.9% rRNA and 1.8% tRNA sequences. The sequence “GGAGG”, the Shine-Dalgarno sequence, was also found upstream of ten distinct protein coding genes: *rbcL*, *psaA*, *psaB*, *psbZ*, *psbD*, *rpoC2*, *psbJ*, *psbF*, *psaJ* and *ycf2*. Additionally, 12 distinct genes contain introns, of which three (*clpP*, *ycf3* and *rps12*) have two introns. Of these 12, three intron-containing genes (*ndhB*, *rpl2* and *trnA*) are duplicated on the IRs, taking the total to fifteen. Although *rps12* is a trans-spliced gene, it also contains an intron that resides on both IRA and IRB. When aligned, the Pongamia and *L. japonicus* cpDNA share a pairwise identity of 82.4%. In addition, as with other cpDNA, the Pongamia cpDNA is AT rich (i.e. the GC content is 34.8%).

**Figure 2:**
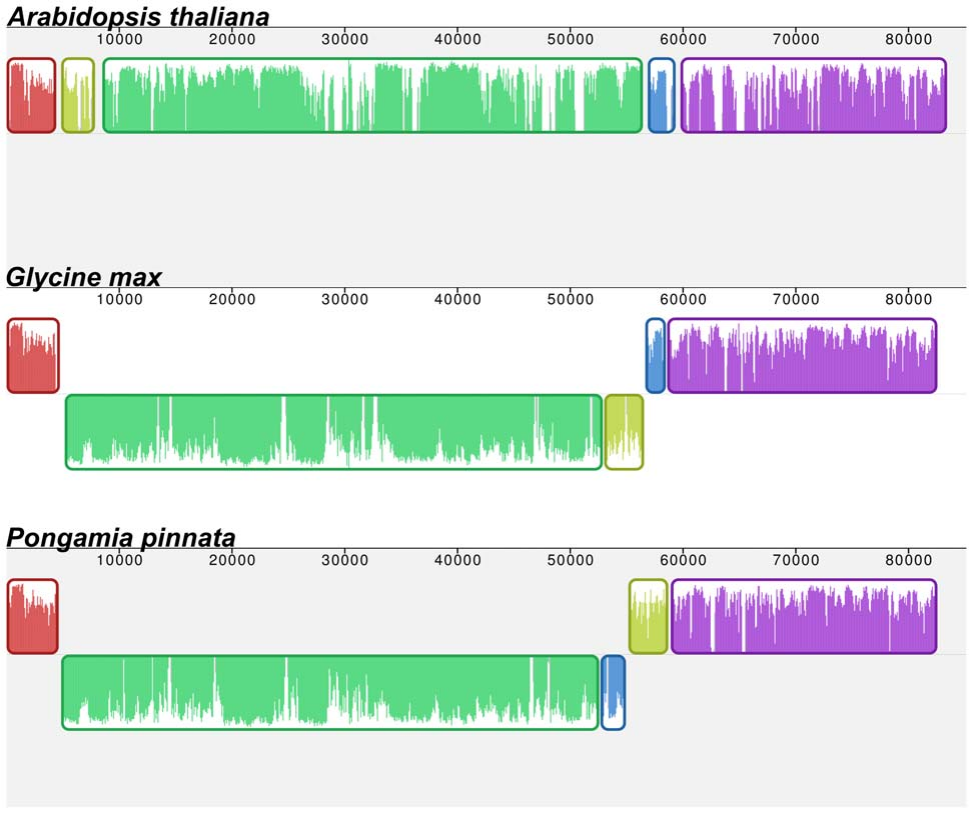
Mauve alignment of the Pongamia cpDNA LSC to representatives of the flowering plants (*Arabidopsis thaliana*) and Fabaceae (*G. max*) showing a 50 kb inversion that is a unique event in the evolution of the Fabaceae (green and gold blocks) as well as a 6.5 kb inversion that is unique to the Pongamia cpDNA (gold and blue blocks). Plots of similarity can be found inside of each element.

Structurally, the Pongamia cpDNA is similar to other legume cpDNA containing a set of IRs. It contains the 50 kb inversion that is unique in the evolution of the Fabaceae [7]. In addition, the Pongamia cpDNA also contains a small inversion of approximately 6.5 kb that appears unique when compared to other species of flowering plants (Fig. 2). This inversion reverses and complements about 6.5 kb across the second junction of the 50 kb inversion, leaving *psaI*, *accD*, *rps16*, *trnQ* and *trnS* to be transcribed in the opposing direction and *psbK* and *psbI* to be transcribed in the reverse direction, relative to the start of the LSC. Further, no disruption to the coding or regulatory sequences of the impacted genes has taken place.

A highly AT rich region between 14,400 bp and 14,600 bp downstream of the start of the LSC was identified as the genome’s origin of replication, *oriC*, by comparison with currently available origin of replication sequences in the NCBI databases. Additionally, the relative coverage of Pongamia cpDNA by DNA sequence reads during assembly (Fig. S1), shows significantly lower coverage in this location (Fig. S2). Although lower coverage at this position was also identified during the re-assembly of the *L. japonicus* cpDNA (Fig. S3), identification of an *oriC* or palindromic sequence was limited (Fig. S4). Despite this, a region believed to surround the *L. japonicus oriC* (between positions 14,500 bp and 14,900 bp; Fig. S5), was also AT rich.

### Organization and Structure of the Pongamia and *L. japonicus* Mitochondrial Genomes

**Figure 3:**
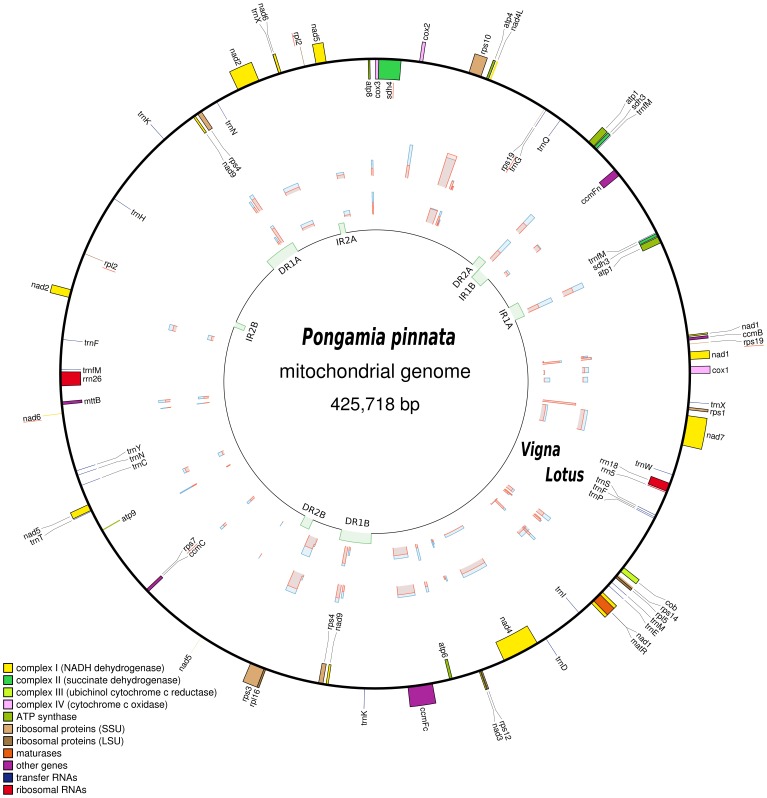
Map of the Pongamia mtDNA (425,718 bp). The innermost circle indicates the locations of two sets of inverted repeats (IR1A and IR1B, 6,229 bp; IR2A and IR2B, 2,274 bp) and two sets of direct repeats (DR1A and DR1B, 13,319 bp; DR2A and DR2B, 3,919 bp). Furthermore, IR1B and DR2A share an overlap of 669 bp. Genes on the outside of the map are transcribed in a counter-clockwise direction and those on the inside clockwise. The graphs plotted between these circles represent the percentage synonymous (blue) and non-synonymous (red) divergence of each protein-coding gene, between Pongamia and *V. radiata*, and, Pongamia and *L. japonicus*. Genes underlined in red are pseudogenes.

**Figure 4:**
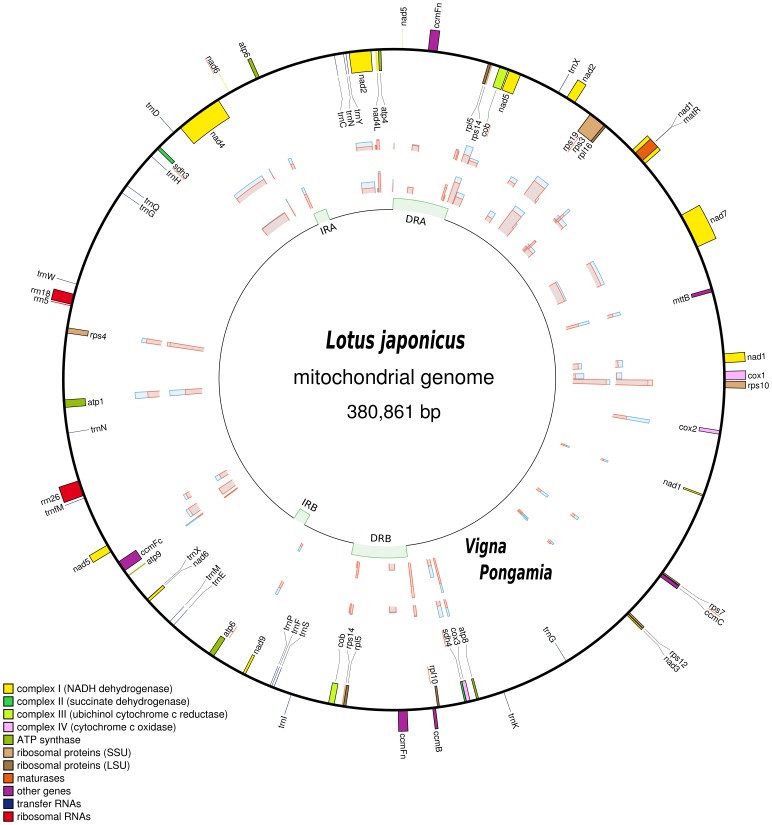
Map of the *L. japonicus* mtDNA (380,861 bp). The innermost circle indicates the locations of a set of inverted repeats (IRA and IRB, 4,460 bp) and a set of direct repeats (DRA and DRB, 18,971 bp). Genes on the outermost of the map are transcribed in a counter-clockwise direction and those on the inside clockwise. The graphs plotted between these circles represent the percentage synonymous (blue) and non-synonymous (red) divergence of each protein-coding gene, between *L. japonicus* and *V. radiata*, and, *L. japonicus* and Pongamia. Genes underlined in red are pseudogenes.

The Pongamia mtDNA (Fig. 3) and *L. japonicus* mtDNA (Fig. 4) were determined to be 425,718 bp and 380,861 bp in size, respectively. The Pongamia mtDNA contains two sets of inverted repeats and two sets of direct repeats (Fig. S6). The first set of inverted repeats, IR1A and IR1B, are each 6,229 bp whilst the second set of inverted repeats, IR2A and IR2B, are each 2,274 bp. The first set of direct repeats, DR1A and DR1B, are each 13,319 bp whilst the second set of direct repeats, DR2A and DR2B, are each 3,919 bp. It should be noted that IR1B and DR2A share an overlap of 669 bp and that this overlap may be of significance when considering the secondary structure and folding of this genome. Comparatively, the *L. japonicus* mtDNA is structurally much simpler than that of the Pongamia mtDNA. It contains a single set of inverted repeats, IRA and IRB, each of 4,460 bp and a single set of direct repeats, DRA and DRB, each of 18,971 bp (Fig. S7). Furthermore, the Pongamia mtDNA contains the same intron containing and trans-spliced protein-coding genes that could be found in the *L. japonicus* mtDNA.

The Pongamia mtDNA contains 71 features, of which there are 37 protein-coding genes, three rRNA genes, 24 tRNA genes and seven pseudogenes. We found 33 distinct protein-coding genes, three distinct rRNA genes, 22 distinct tRNA genes and six distinct pseudogenes. Hence, the Pongamia mtDNA contains duplicated genes that reside in repeat regions. Three genes, *trnfM*, *sdh3* and *atp1*, reside in IR1A and IR1B, and three more, *rps4*, *nad9* and *trnK*, reside in DR1A and DR1B. Although no genes were found to reside in IR2A and IR2B or DR2A and DR2B, an 87 bp fragment of *rpl2* was found in IR2A and IR2B. It is also worth noting that none of the 71 features found in the Pongamia mtDNA were found to reside across any of the repeat region boundaries. When looking for the list of 17 genes frequently lost during angiosperm evolution, including *rpl2*, eight of these were either absent from the Pongamia mtDNA (*rpl10, rps2*, *rps11*, *rps13*) or were found as pseudogenes in various stages of attrition (*rpl2*, *rps7*, *rps19*, *sdh4*). Surprisingly, Pongamia *sdh3* did not make the list of pseudogenes. It was found translationally uninterrupted and divergence from the *L. japonicus* mitochondrial pseudo-orthologue was calculated at 11.3%. In contrast, Pongamia *sdh4* remains a pseudogene, the open reading frame is interrupted not only by a 439 bp fragment of cpDNA, but also by over 3 kb of flanking DNA of unknown origin. Additionally, we were able to locate a single *rps7* fragment as well as two *rps19* fragments, suggesting the transfer of these genes to the nucleus. We propose that one fragment represents the start of the *rps19* gene and the other represents the end of the *rps19* gene, as these sequences do not overlap. Comparative analysis also revealed numerous short fragments with strong similarity to known functional genes. While many of these could be considered illegitimate, a partial match (90 bp) to *nad6* was detected. Interestingly, this fragment is not at all similar to an orthologous fragment found in the *L. japonicus* mtDNA. We also calculated a gene density of 8.0% protein-coding, 1.2% rRNA and 0.4% tRNA gene sequences as well as a GC content of 45.0%.

The *L. japonicus* mtDNA contains six fewer genic features than the Pongamia mtDNA, with three rRNA genes, 20 tRNA genes, 34 protein-coding genes and eight pseudogenes. Of the 34 protein-coding genes, 31 are unique. Hence, like the Pongamia mtDNA the *L. japonicus* mtDNA contains duplicated genes that reside in repeat DNA. However, the genes that can be found in the *L. japonicus* mtDNA are different to those found in the repeat regions of the Pongamia mtDNA. These genes, *ccmFn*, *rpl5* and *rps14*, are each duplicated with single copies of each on DRA and DRB. These direct repeats also lead to partial duplication of *cob*. As such, whilst a complete 621 bp copy of *cob* is positioned across DRB, a *cob* pseudogene resides across the boundary of DRA. The inverted repeats also lead to partial duplication of *atp6*. Here, a complete 720 bp copy of *atp6* resides across the IRA boundary, with an *atp6* pseudogene across the IRB boundary. We also interrogated the *L. japonicus* mtDNA for a list of 17 genes (15 ribosomal protein and two respiratory) that are well understood to have been lost frequently during angiosperm evolution. Ten of these were either absent from *L. japonicus* mtDNA (*rpl2*, *rps1*, *rps2*, *rps11*, *rps13*) or were present as pseudogenes in various stages of attrition (*rpl10*, *rps7*, *rps19*, *sdh3*, *sdh4*). The *rpl10* and *sdh3* pseudogenes were the most intact, were both created by a single point mutation and each contain just one internal stop codon. Another gene, *sdh4*, is also very much intact; however, multiple frame-shift mutations have disrupted the reading frame and introduced a premature stop codon. A 333 bp fragment of *rps7* was also located immediately downstream of *ccmC*. It contains an internal stop codon and lacks almost 40 codons from the 5′ end of the gene that are likely required for functionality. We also found eight intron-containing genes, of which three (*nad1*, *nad2*, *nad5*) are trans-spliced. Sensitive BLAST searches of this genome also revealed numerous short DNA fragments (<50 bp) with strong similarity to a number of genes. Another hit was to *nad6*, and this represents the eighth pseudogene. The *L. japonicus* nuclear genome BLAST databases [12] were also interrogated to confirm the absence of *rps1*. Our preliminary results conclude that *rps1* has been transferred to the nuclear genome where a functional copy of *rps1* exists. Furthermore, the genome was found to be almost 10% gene dense; it consists of 8.2% protein-coding, 1.3% rRNA and 0.4% tRNA sequences with a GC content of 45.4%.

### RNA Editing, Codon Usage and Utility of Legume Organelle DNA

Codon usage and putative RNA editing sites of Pongamia and *L. japonicus* cpDNA and mtDNA were determined using the organellar genomes of *Vigna radiata* [10], [11] as references (Table S1). Using the CURE software, we were able to predict 556, 517 and 485 C-to-U RNA editing sites on gene coding sequences in the *L. japonicus*, Pongamia and *V. radiata* mtDNA, respectively. C-to-U RNA editing was predicted at the start of *nad1*, *nad4L* and *rps10* as well as the stop of *atp6*, *ccmFc* and *rps10* for all three species. This type of editing may also be required for the initiation of the Pongamia *mttB*. The mitochondrial *nad5* of *V. radiata* is also reportedly initiated by C-to-U RNA editing; however we were unable to predict this site using CURE. During our analysis of RNA editing and codon usage it was necessary to adjust the start sites of *rpl16* and *mttB* and stop sites of *rps10* and *atp6* of the *V. radiata* mtDNA. CURE-Chloroplast software was also used to predict 50 C-to-U RNA editing sites in the cpDNA of the same three species. Although RNA editing does not create any in-frame stop codons, *psbL* and *ndhD* often start at ‘ACG’. However, with a total of only 50 RNA editing sites, analysis of codon usage after RNA editing is limited (Table S2). As expected, the codon usage pattern of all three cpDNA was also found to be highly similar (comparison not shown).

Both before and after RNA editing, similarity was found between the codon usage patterns of all three mtDNA. When comparing the codon usage of cpDNA and mtDNA of Pongamia, both before and after RNA editing, most of the amino acids of the cpDNA that encode proteins had a stronger association for a particular codon or set of codons. However, when we look at the frequency of particular codons, some points of difference could be noticed. For example, when compared to the Pongamia mtDNA, the Pongamia cpDNA has a higher frequency of codons for glutamic acid. Yet, when we look at the frequency of codons for phenylalanine, for example, RNA editing increases the number of ‘UUU’ and ‘UUC’ codons on the Pongamia mtDNA by over 10%. Interestingly, the number of ‘UUU’ and ‘UUC’ codons created by RNA editing is similar to the number of like codons found on the Pongamia cpDNA. We also notice a preference of codons for leucine, and indifference for proline, arginine and serine.

Mapping transcriptomic data [13] to Pongamia organellar DNA has also provided evidence for the computationally predicted RNA editing sites (Table S3 and Table S4, respectively). This data was also used to confirm protein-coding gene splice-site junctions as well as to provide candidate chloroplast and mitochondrial genes for further gene expression studies involving leaf (Table S5) and root (Table S6) tissue, under stress conditions involving salt-water treatment.

### Legume Organelle Protein-coding Gene Sequence Divergence

We measured the divergence of almost 120 organellar protein-coding genes from the three legume species (Table S7). Despite the structural fluidity of plant mtDNA and the frequent lack of structural conservation, synonymous and non-synonymous divergence of mitochondrial protein-coding genes was approximately 5.26- and 3.74-fold lower than the divergence of the chloroplast protein-coding genes, respectively. On average, synonymous divergence of the chloroplast genes of *L. japonicus* and Pongamia, Pongamia and *V. radiata*, and *V. radiata* and *L. japonicus* were calculated at 7.99%, 7.49% and 9.62%, respectively. Synonymous divergence of the protein-coding mitochondrial genes of the same groupings of species was calculated at 1.60%, 1.29% and 1.87%, respectively. These results simply indicate that chloroplast genes have a higher rate of nucleotide substitution. However, when focusing on non-synonymous mutations, of the cpDNA and mtDNA, a high degree of identity was noticed between *L. japonicus –* Pongamia (91.58% and 97.74%, respectively) and Pongamia *– V. radiata* (91.53% and 97.7%, respectively). When compared to these groups, non-synonymous identity of the cpDNA and mtDNA of the *V. radiata – L. japonicus* group was slightly lower at 89.91% and 97.37%, respectively. This of course suggests that the *V. radiata* and *L. japonicus* are the most divergent of the three species.

### Phylogenesis and the Promiscuity of Plastid DNA

After compiling all the published organelle genome sequences of the Streptophyta, we present the relatedness of the Pongamia and *L. japonicus* cpDNA (Fig. S8) and mtDNA (Fig. S9). The cpDNA alignment makes obvious the separation of the Fabaceae into two groups based on the presence or absence of two copies of the inverted repeat. Whilst the mitochondrial phylogram appears to simply confirm the relatedness of the Fabaceae, it also suggests that the nuclear genomes of *Glycine max*, *Phaseolus vulgaris*, *V. radiata* and *L. japonicus* are worth investigating when attempting to study the Pongamia nuclear genomic environment.

The *L. japonicus* mtDNA contains six tRNA genes of chloroplast origin. Of these, there is one copy each of the aspartic acid, histidine, tryptophan, and methionine tRNA genes, and two copies of the asparagine tRNA gene. We found an identical set of tRNA genes in the Pongamia mtDNA, however, one copy of the tRNA for asparagine folds with a low Cove score, the product of which is most likely dysfunctional. BLAST was also able to identify four and ten other chloroplast-like features that appear to be present in both the Pongamia and *L. japonicus* mtDNA, respectively. Two of the ten fragments of cpDNA in the *L. japonicus* mtDNA have been incorporated into the genome’s direct repeat. Thus in total, the *L. japonicus* mtDNA comprised 1.52% cpDNA, whilst the Pongamia mtDNA is made up of just 0.70% cpDNA.

Similarity between the Pongamia and *L. japonicus* organellar repeat regions was also measured (Fig. S10). Additionally, the GC content of these repeats was also calculated. We found that while the IRs of the cpDNA were 41.6% GC, the LSC and SSC regions had lower GC content, at 32.2% and 28.0%, respectively. Interestingly, the Pongamia and *L. japonicus* mtDNA repeat elements had very similar GC content, of approximately 45%, when compared to their non-repetitive counterparts.

## Discussion

Pongamia seed oil is going to play an important role as a sustainable feedstock in the future bioenergy market. We have added to the genetic knowledge of this poorly-understood species with the sequencing and annotation of its two organellar genomes. Our study not only exemplifies the use of 2GS and in-house developed assembly software, but also supports further domestication of this emerging species. For example, Pongamia will benefit greatly from further phylogenetic and phylogeographic analysis. As a tree crop, selection and breeding for crop improvement is especially slow where key phenotypic traits, such as seed oil quality and yield, are only expressed in a mature form. However, its out-crossing nature, although problematic for plantation management, provides germplasm with a diverse range of genotypes and phenotypes. To resolve inter- and intra-population relationships, Pongamia organelle DNA could be used to develop robust genetic markers, based on their high level of conservation. Variation in the reporting of these markers would ultimately assist future breeding programs and greatly accelerate the selection of genetically superior haplotypes. Additionally, the ability to distinguish individuals from a population of trees will also provide insight into cultivar diversity and overall population structure.

In many angiosperms, cytoplasmic inheritance is based on preferential transmission of cpDNA and mtDNA. Many traits have a maternal input. For example, we observed that while Pongamia seeds vary significantly in mass from tree to tree, seeds from a single parent tree show less variation in mass, suggesting maternal, physiological or genetic effects. Similarly, the susceptibility of a plant to a particular pathogen is often controlled by cytoplasmic genes; the catastrophic outbreak of Corn Blight in the USA in 1971 was solely contributed to the conservative breeding of corn lines based on just one “Texas” cytoplasm. Critical parts of fatty acid biosynthesis such as chain elongation and desaturation occur in the plastid. The detailed knowledge of the genomes maintaining the integrity of these critical organelles is thus pivotal for the future application of plant biotechnology to Pongamia improvement.

Using our sequencing and assembly pipeline we were also able to assemble the mtDNA of the model legume *L. japonicus*, thus allowing further insight into Pongamia through genomic comparison. For example, both legumes likely maintain multipartite organization using numerous sets of direct and inverted repeat sequences. However, Pongamia mtDNA harbours twice the number of repeat elements and, as a consequence, sustains a multipartite structure of proportional complexity. Additionally, the size of the repetitive sequences is directly proportional to their recombination frequency [14]. Thus, the larger repeat elements of Pongamia (particularly the large direct repeats) are more likely than their smaller counterparts to be involved in repeat-mediated intramolecular recombination. Whilst the repeat elements of the Pongamia and *L. japonicus* mtDNA may have varying rates of recombination activity, and thus an altered abundance of subcircular and isomeric molecules, both genomes retain a similar amount of repeat sequence relative to their size (12.09% and 12.30%, respectively). Hence, in terms of repeat-mediated homologous recombination, neither molecule appears likely to be significantly more or significantly less active than the other.

Although it is difficult to accurately elucidate *in silico* the secondary and tertiary structures of complex organellar genomes, the positions and orientations of recombinantly active repeats can be used to predict the sizes and isoforms of the derivatives of the master circle. The existence of DR1A and DR1B in the Pongamia mtDNA suggests that two subcircles of 264,796 bp and 160,922 bp are generated through intramolecular recombination, of which the former would have two isoforms due to the presence of IR1A and IR1B. Additionally, the presence of DR2A and DR2B adds another two subcircles of 230,592 bp and 195,126 bp. In this case, both subcircles could give rise to two isomeric circles that differ from their respective subcircles by segmental inversion with the former the result of IR2A and IR2B and the latter the result of IR1A and IR1B. Comparatively, the *L. japonicus* mtDNA would generate only two subcircles of 199,194 bp and 181,667 bp, the first of which would have two isoforms due to the presence of IR1A and IR1B. Although our predictions are for subcircles and isomeric circles (including isomers of the master circles), it is likely that electrophoresis and/or electron micrographs of Pongamia mtDNA, and indeed *L. japonicus* mtDNA, would show linear and circular DNAs of a variety of sizes with complex branched structures.

Further insight into Pongamia was realized via the comparative analysis of gene content with another legume, *V. radiata*. We found that Pongamia maintains the mitochondrial-encoded *cox2* and *rps1* that are otherwise absent in the *V. radiata* and *L. japonicus* mtDNA, respectively. Pongamia also maintains the respiratory gene, *sdh3*, encoding a key subunit of succinate dehydrogenase. Ribosomal protein genes, such as *rps1*, are frequently lost during angiosperm evolution and respiratory genes, such as *cox2* and *sdh3*, are lost only rarely [15]. In this sense, Pongamia mtDNA has remained relatively static compared to the other two legumes. We did however notice the pseudogenization and loss of multiple ribosomal genes, reflecting the ongoing loss and transfer of functionality to the plant’s nuclear genome. Our analysis reveals that another respiratory gene, *sdh4* (encoding a different subunit of succinate dehydrogenase), has been pseudogenized from all three legumes. Sequence comparison with the Pongamia transcriptomic datasets found an intact *sdh4* coding sequence, suggesting that this gene has been functionally transferred to the nucleus, as is the case with other legumes, including *G. max*, *Medicago truncatula* and *L. japonicus*. Relatively intact pseudogenes, such as *sdh4*, indicate that the gene has been lost or transferred to the host nuclear genome only recently. These events afford further opportunity to study the process of gene transfer, and thus insight into the establishment and evolution of the eukaryotic organelles. Furthermore, functional studies of their nuclear isoforms may reveal novel N-terminal presequences that can be used to target foreign proteins to the mitochondria.

Insight into plant organelle genomes was gained via the analysis of RNA editing sites, codon usage and GC content. By means of computational analysis, we confirmed that legume mtDNA contains a similar number of RNA editing sites, relative to their size and gene content. We have also used transcriptomic data to provide evidence of each RNA editing site (Table S3 and Table S4), and this may be used to predict novel, and perhaps even non-canonical, RNA editing sites. Our analysis also revealed that all three legume mtDNA have highly similar codon usage patterns and GC content. The comparatively slower mutation rate of the mtDNA, as compared to cpDNA, is kept in-check by a high GC content which, through increased thermostability, is vital for cellular longevity. Similarly, we propose that angiosperms maintain, at least in part, a large genome size with expanded inter-genic regions to further protect against cellular mutagens, such as ultraviolet light and reactive oxygen species [16]. We found rather ordinary synonymous and non-synonymous divergence rates of mitochondrial protein-coding genes, of approximately 5.26- and 3.74-fold lower respectively, compared to their chloroplast counterparts (as demonstrated in Table S7) and similar to previous findings [9]. Thus, maintaining a low mutation rate is critical for genic conservation and cellular permanence.

When characterizing the Pongamia cpDNA we found a novel 6.5 kb inversion responsible for the silent, re-orientation of five protein-coding and two tRNA genes. Furthermore, phylogenetic and sequence analysis of Pongamia places it firmly inside the subclade of tropical legumes. Hence, Pongamia is a legume that contains two copies of an inverted repeat and like other legumes, such as *G. max*, *V. radiata* and *P. vulgaris*, Pongamia generally produces spherical, determinate nodules confirming previous observations [2]. The Pongamia cpDNA will not only make a valuable phylogenetic tool, but will also allow development of a plastid engineering protocol. Such a feat would allow traits such as oil quality and content to be manipulated, critical for the large scale cropping of the species. Pongamia cpDNA could also be utilized through hyperexpression and a transplastomics approach to produce large amounts of foreign protein (up to 70% of the total soluble protein [17]) inside desirable transgenic tissues, such as the leaf and seed. For example, the foliage of Pongamia is surplus to the conventional requirements for seed oil harvest, and as a waste product from plantation management, could be better used to produce alternate high value products such as vaccines or pharmaceutical proteins often referred to as “molecular farming”. Alternatively, leaves could be ‘filled’ with oil, allowing the harvesting of the tree’s canopy during general plantation maintenance and greatly improving yield through means of pyrolysis and bio-oil [18].

Our re-sequencing and assembly of the *L. japonicus* cpDNA not only confirms work conducted by the KAZUSA group ten years ago, but also shows that SaSSY is capable of assembling novel prokaryotic-like genomes (even with low coverage datasets). Consequently, our analysis afforded us the opportunity to study a potentially high value plant species that is yet to be domesticated. We developed a baseline understanding of Pongamia which was built upon by mapping recently published transcriptomic data. We have identified candidate organellar salt responsive genes that may be used to gain insight into how Pongamia copes with stress under mangrove-like conditions. Finally, the organellar genomes of Pongamia presented here will serve as reference sequences or quality controls when assembling and further investigating its nuclear genome.

## Methods

### The SaSSY Assembler and Pipeline

*De novo* second-generation DNA sequencing of the Pongamia and *L. japonicus* organellar genomes was performed using samples of leaf-derived DNA (whole genomic DNA). We used the Illumina® GAIIx platform to generate 12,501,168 Pongamia paired-end sequences of 36 bp from genomic fragments of approximately 390 bp (Pon_03_001), as well as 29,474,558 mate-pair sequences of 75 bp with fragment lengths of approximately 3 kb (Pon_37_001). It should be noted that this large-insert library also contained a shadow (paired-end) library with a fragment length of approximately 220 bp. The same platform was also used to generate 65,930,582 *L. japonicus* paired-end sequences of 100 bp from genomic fragments of approximately 350 bp (LjDIMG_03_001). These three datasets have been deposited into the NCBI Short Read Archive (SRA) database under the accession number: SRA051251. Information regarding experimental methods, sample preparation and library construction can also be found under this resource.

SaSSY [4] (v0.1.1.3) is a short paired read *de novo* assembler written in C++ utilizing a novel set of algorithms. It was designed for the assembly of repetitive genomes, specifically plant BAC sequences. As an overlap-layout assembler, SaSSY avoids cutting reads into k-mers and building de Bruijn graphs [19]. Instead, SaSSY joins two reads by an edge if their offset (the length of the non-overlapping parts of two overlapped reads) is less than a specified maximum value. SaSSY is able to use paired read information to produce scaffolded contigs and has proved useful in a number of in-house projects. SaSSY has not been published but is freely available. For more information as well as a detailed description of the algorithm, please read the documentation available at http://sassy.mikeimelfort.com/.

We validated our assembly approach by re-assembling the *L. japonicus* cpDNA using the LjDIMG_03_001 paired-end dataset described above. SaSSY allows the user to alter two parameters; naïve and extension offsets which are used to determine the minimum overlap needed to assemble reads. To re-assemble the *L. japonicus* cpDNA, naïve and extension offsets of 2 and 6 were used respectively, as well as a readlength of 63 bp. These assembly parameters were chosen based upon recommendations in the SaSSY manual. When we aligned our re-assembly of the *L. japonicus* cpDNA to the published sequence [6], we found a single SNP (a ‘G’ to a ‘T’ substitution) at position 124,653. This change impacts *ycf1* at the codon for the 409^th^ amino acid changing it from a threonine to an asparagine. Confident of the reliability of SaSSY, we assembled the *L. japonicus* mtDNA and the two Pongamia organellar genomes. To re-assemble the *L. japonicus* mtDNA, we used SaSSY with naïve and extension offsets of 8 and 12, and a readlength of 63 bp respectively. We used the same parameters and a readlength of 35 bp to assemble the Pongamia cpDNA. To assemble the Pongamia mtDNA, we used SaSSY’s default offsets of 4 and 12, however we first cut and filtered the raw data using the Perl script ‘cut_Down_ILL.pl’ included in the SaSSY package. This script first trims the reads and then cuts the resulting sequence into a number of short fragments resulting in an increased number of fragmented reads. The user must specify the trim length and the amount that the start of each new fragment is offset from the next. The 75 bp reads were processed two times using an offset of 4 bp, an “out” readlength of 35 bp and trim lengths of 36 bp and 32 bp resulting in 58,949,116 and 88,423,674, shorter fragments respectively. We then ran SaSSY twice with a readlength of 35 bp and in each run we added the ‘Pon_03_001’ dataset.

In order to further scaffold and complete the genome assemblies, it was necessary to determine the origins of the contigs; as either chloroplast-, mitochondrial-, or nuclear-like DNA sequence. We did this by comparison with the nucleotide sequence database (v4) at the NCBI using BLAST+ (v2.2.24) with default search parameters. We also interrogated our datasets with these contigs using MegaBLAST [20] (v2.2.21): `megablast -a 24 -F F -W 12`. For each read in a ‘hit’, we applied a minimum alignment filter of 60 to the *L. japonicus* and Pongamia mate-pair libraries and a filter of 30 to the Pongamia paired-end library using Perl (v5.10.1). Perl script was also used to generate insert-size distribution and coverage statistics which were in turn used to visually assess and validate the structural integrity of these assemblies allowing re-assembly as necessary. All single base assembly errors and small indels were corrected by mapping the reads back onto the assemblies using BWA [21] (v0.6.1) and SAMtools [22] (v0.1.18). Tablet [23] (v1.12.03.26) was used for viewing the mapping files. An in-house Perl script with the Bio::DB::Sam module was also used to refine the assembly. Geneious Pro [24] (v5.4.6) was then used to circularize and orientate the sequences, as well as to correct annotation errors. We also generated large contigs with Velvet [25] (v1.2.03) and mapped these with high accuracy to their respective genomes to confirm our SaSSY assemblies.

### Gene Discovery and Annotation

The Pongamia cpDNA was annotated using DOGMA [26]. Nucleotide and CDS databases of flowering plant mtDNA were compiled, and the mtDNA assembled from this study were then interrogated against these databases to find protein and RNA genes using BLASTN and BLASTX. Blast2GO [27] was also used to find homologous sequences and confirm functional annotation. We considered annotation with a mean similarity greater or equal to 50% and an e-value smaller than 1E^−20^ with five or more hits. We manually curated these annotation databases, removing any ambiguous, uninformative or inaccurate entries (including those classed as hypothetical or unclassified). To identify tRNA genes, tRNAscan-SE [28] (v1.12) and the available web server [29] (http://lowelab.ucsc.edu/tRNAscan-SE) were utilized. To predict the bulk of mitochondrial and chloroplast RNA editing (cytidine to uridine) sites the computational tools CURE [30] (http://59.67.33.228/biosrv/cure/, v1.1) and CURE-Chloroplast [31] (http://bioinfo.au.tsinghua.edu.cn/software/pure, v1.0) were used, respectively. The four available transcriptomic datasets (described above) were combined and re-mapped against each of the 110 unique coding-sequences for potential RNA editing sites. This was done using our in-house Perl script with the Bio::DB::Sam module. Repeat regions as well as the designated chloroplast origin were identified with the nucleotide sequence database (v4) at the NCBI using BLASTN with default parameters. Genome sequences and annotations for the Pongamia chloroplast and mitochondrion, as well as the mitochondrion of *L. japonicus*, can be found in the GenBank data library under accession numbers JN673818, JN872550 and JN872551, respectively. All GenBank files contain predicted RNA editing sites. Genome map construction was performed with OGDraw [32] and Circos [33].

### Transcriptomic Mapping and Analysis

Four transcriptomic datasets were downloaded from the NCBI SRA under the accession number SRA046342. Each dataset was produced using the Illumina GAIIx platform and each consists of 24 million 75 bp paired-end reads taken from pooled samples of leaf or root tissue at two hours, four hours and eight hours under application of either fresh- or salt-water. These datasets were used to confirm predicted splice-site junctions of the Pongamia organellar whole genome sequences using TopHat [34] (v2.0.0). They were then re-mapped using BWA to a list of 110 unique protein-coding genes (deduced using methodology discussed above) taken from the two Pongamia organellar genomes. Using SAMtools, the mapping files were normalized to Reads Per Kilobase of exon model per Million mapped reads (RPKM) [35]. Unfortunately these datasets were not produced with any biological replication, thus statistical testing for the significance of the salt treatment was limited.

### Phylogenesis and Divergence

The cpDNA of nearly 190 species of Streptophyta and the mtDNA of 36 species in the same phylum were aligned using the progressiveMauve [36] (v2.3.1) algorithm. The alignment output was used to create three rectangular phylograms with TreeGraph [37] (v2.0.47). Whilst the mitochondrial phylogram shows all 36 species, a subset of 23 cpDNA (the most closely related species with respect to Pongamia) were taken to improve tree readability. Mauve [38] (v2.3.1) was also used to analyse genome alignment output. Divergence percentages were calculated: 100% minus pairwise percent identity. ClustalW2 [39] (v2.1) output was parsed using Perl script to calculate pairwise percent identity. This was computed in the same way that Geneious Pro calculates pairwise percent identity: by looking at all pairs of bases at each column position and scoring a hit, divided by the total number of pairs, for each column when they are identical.

## Supporting Information

Figure S1 Coverage (blue and green lines) and insert-size distribution (red and yellow dots) statistics generated during assembly of the Pongamia cpDNA using the two insert libraries Pon_03_001 and Pon_37_001, respectively. Each dot represents the middle point between two paired-end or mate-pair reads. A break in coverage or insert-size distribution of both libraries at the same position would indicate mis-assembly. Likewise, small decreases or small increases would suggest regions of excessive or inadequate overlapperation, respectively.
(TIF)

Figure S2 Coverage (blue and green lines) and insert-size distribution (red and yellow dots) statistics similar to those described in Figure S1 of the site surrounding the Pongamia chloroplast origin of replication. A highly AT rich and also lowly covered region between 14,400 bp and 14,600 bp downstream of beginning of the LSC has been annotated *oriC*.
(TIF)

Figure S3 Map of the *L. japonicus* cpDNA (150,519 bp). The innermost circle indicates the locations of the IRs (IRA and IRB, 25,156 bp), which separate the LSC (81,936 bp) and SSC (18,271 bp) regions. Genes on the outermost of the map are transcribed in a counter-clockwise direction and those on the inside clockwise. The graphs plotted between these circles represent the percentage synonymous (blue) and non-synonymous (red) divergence of each protein-coding gene, between *L. japonicus* and *V. radiata*, and, *L. japonicus* and Pongamia. The red arrow indicates the position of the replication origin, *oriC*. Genes underlined in red are pseudogenes.
(TIF)

Figure S4 Coverage (blue line) and insert-size distribution (red dots) statistics generated during assembly of the *L. japonicus* cpDNA using the LjDIMG_03_001 insert library. Each dot represents the middle point between two paired reads.
(TIF)

Figure S5 Coverage (blue line) and insert-size distribution (red dots) statistics similar to those described in Figure S4 of the site surrounding the *L. japonicus* chloroplast origin of replication.
(TIF)

Figure S6 Coverage (blue and green lines) and insert-size distribution (red and yellow dots) statistics generated during assembly of the Pongamia mtDNA using the two insert libraries Pon_03_001 and Pon_37_001, respectively. Each dot represents the middle point between two paired-end or mate-pair reads.
(TIF)

Figure S7 Coverage (blue line) and insert-size distribution (red dots) statistics generated during assembly of the *L. japonicus* mtDNA using the LjDIMG_03_001 insert library. Each dot represents the middle point between two paired reads.
(TIF)

Figure S8 Rectangular phylogram of 23 legume-related cpDNA. Legume species boxed in red indicate the indeterminate (maintaining a nodule meristem and encoding Nodule Cysteine-Rich [NCR] peptides involved in bacteroid differentiation) nodulators, while those boxed in blue indicate the determinate (lacking a persistent meristem and NCR peptide genes) nodulators. These red and blue boxes also indicate the IRLC and non-IRLC legume clades, respectively.
(TIF)

Figure S9 Rectangular phylogram of all 36 published mtDNA of species of Streptophyta. Three legume species have been boxed in pink.
(TIF)

Figure S10 Rectangular phylogram of the Pongamia and *L. japonicus* cpDNA and mtDNA repeat regions. A high level of synteny between the inverted repeats of the cpDNA and low level of similarity amongst the mitochondrial repeats is noticeable.
(TIF)

Table S1 Codon usage, scored per thousand bp of all coding sequence, before (in parentheses) and after (in brackets) RNA editing of the *L. japonicus* (top), Pongamia (middle) and *V. radiata* (bottom) mtDNA (usage includes duplicated genes). In each of the three sub-tables, usage has been ranked in order of decreasing frequency, before putative RNA editing.
(DOCX)

Table S2 Codon usage, scored per thousand bp of all coding sequences of the Pongamia chloroplast (including duplicated genes). Usage has been ranked as described in Table S1.
(DOCX)

Table S3 Table of potential RNA editing sites in a list of seventy-seven unique Pongamia chloroplast genes. Positions of these sites refer to the position after the coding sequences’ translational start site. In the table header, an “X” represents the original base (before RNA editing) and a “Y” indicates the change (after RNA editing). An allele balance ratio (ABR) is then listed alongside the evidence supporting the presence of the SNP or indel. A filter of ABR > = 0.40 and coverage > = 3 was applied to these values in an attempt to remove a number of false positives.
(DOCX)

Table S4 Table of potential RNA editing sites in a list of thirty-three unique Pongamia mitochondrial genes. A description of the column headings can be found in the Table S3 legend.
(DOCX)

Table S5 Transcription (RPKM) of Pongamia protein-coding genes in leaf samples that have been treated with either fresh- or salt-water. Cells highlighted in red indicate a minimum 2.5-fold change. Asterisks represent genes that have a 2.5-fold change in both leaf and root samples.
(DOCX)

Table S6 Transcription (RPKM) of Pongamia protein-coding genes in root samples that have been treated with either fresh- or salt-water. A key to this table can be found in the Table S5 legend.
(DOCX)

Table S7 Approximate nucleotide divergence percentages of seventy-seven protein-coding chloroplast and forty-one protein-coding mitochondrial genes common to three legume species, *L. japonicus*, *V. radiata* and Pongamia. Bracketed values represent the corresponding divergence at the amino-acid level. The list of forty-one mitochondrial genes includes seventeen genes (fifteen ribosomal protein and two respiratory; listed last) known to have been lost frequently during angiosperm evolution. For clarity, the duplicates and pseudogenes have been ignored.
(DOCX)
