# Supplementary figures and images for: Capturing the Biofuel Wellhead and Powerhouse: The Chloroplast and Mitochondrial Genomes of the Leguminous Feedstock Tree Pongamia pinnata
Source: PLoS One. 2012 Dec 14;7(12):e51687. doi: 10.1371/journal.pone.0051687 (PMC3522722; doi:10.1371/journal.pone.0051687)

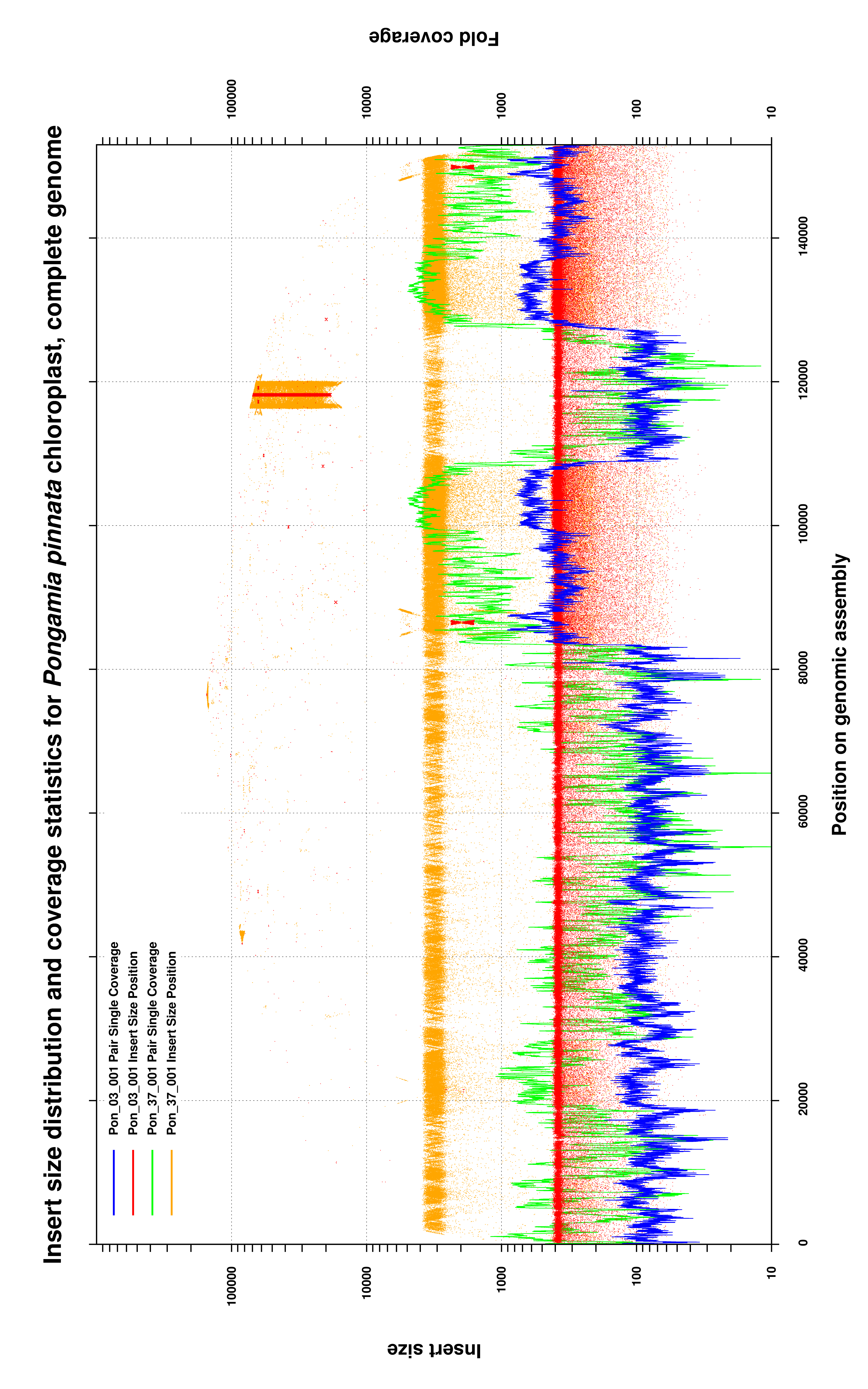

Supplement: Figure S1 — Coverage (blue and green lines) and insert-size distribution (red and yellow dots) statistics generated during assembly of the Pongamia cpDNA using the two insert libraries Pon_03_001 and Pon_37_001, respectively. Each dot represents the middle point between two paired-end or mate-pair reads. A break in coverage or insert-size distribution of both libraries at the same position would indicate mis-assembly. Likewise, small decreases or small increases would suggest regions of excessive or inadequate overlapperation, respectively. (TIF) [file pone.0051687.s001.tif]

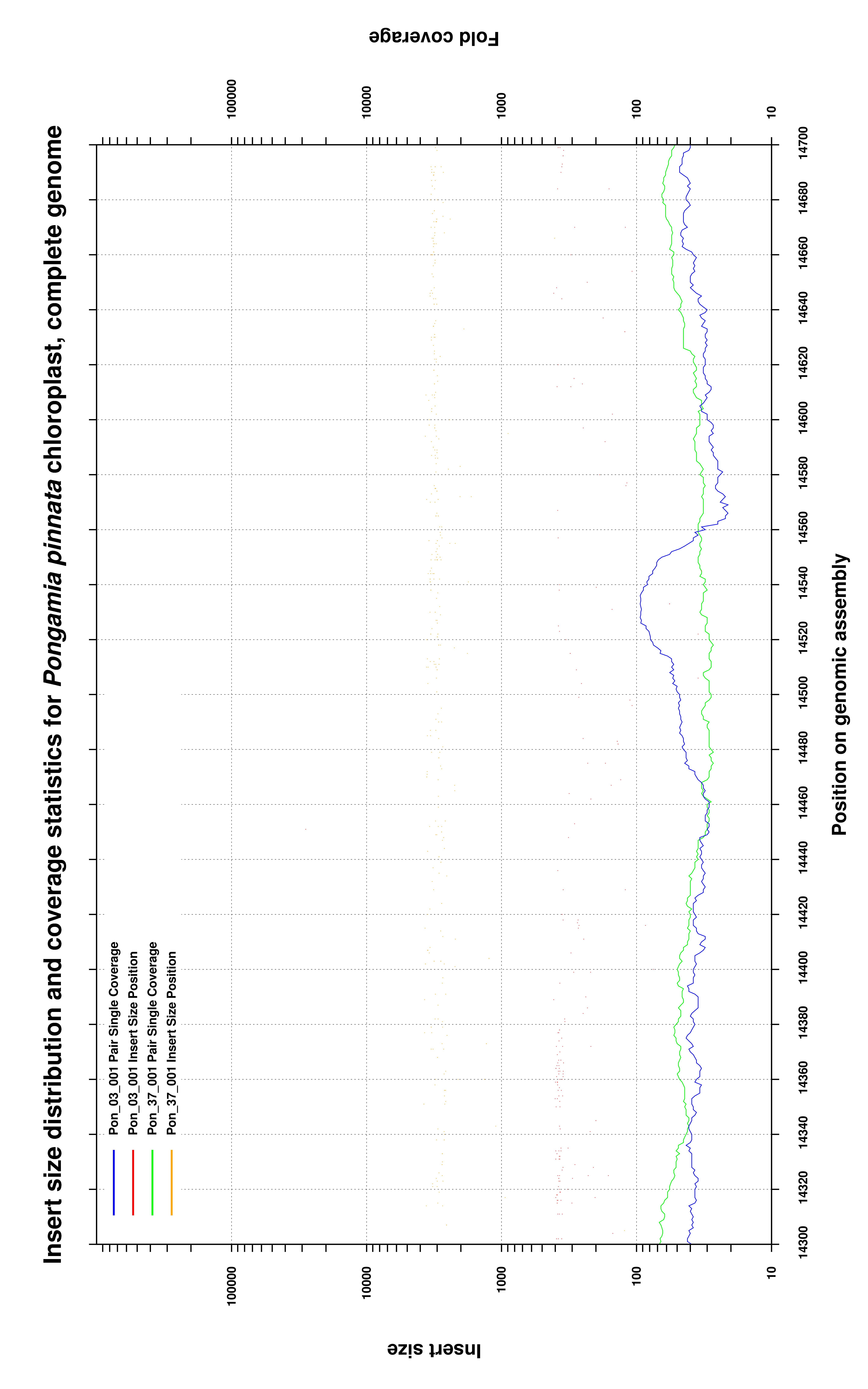

Supplement: Figure S2 — Coverage (blue and green lines) and insert-size distribution (red and yellow dots) statistics similar to those described in Figure S1 of the site surrounding the Pongamia chloroplast origin of replication. A highly AT rich and also lowly covered region between 14,400 bp and 14,600 bp downstream of beginning of the LSC has been annotated oriC. (TIF) [file pone.0051687.s002.tif]

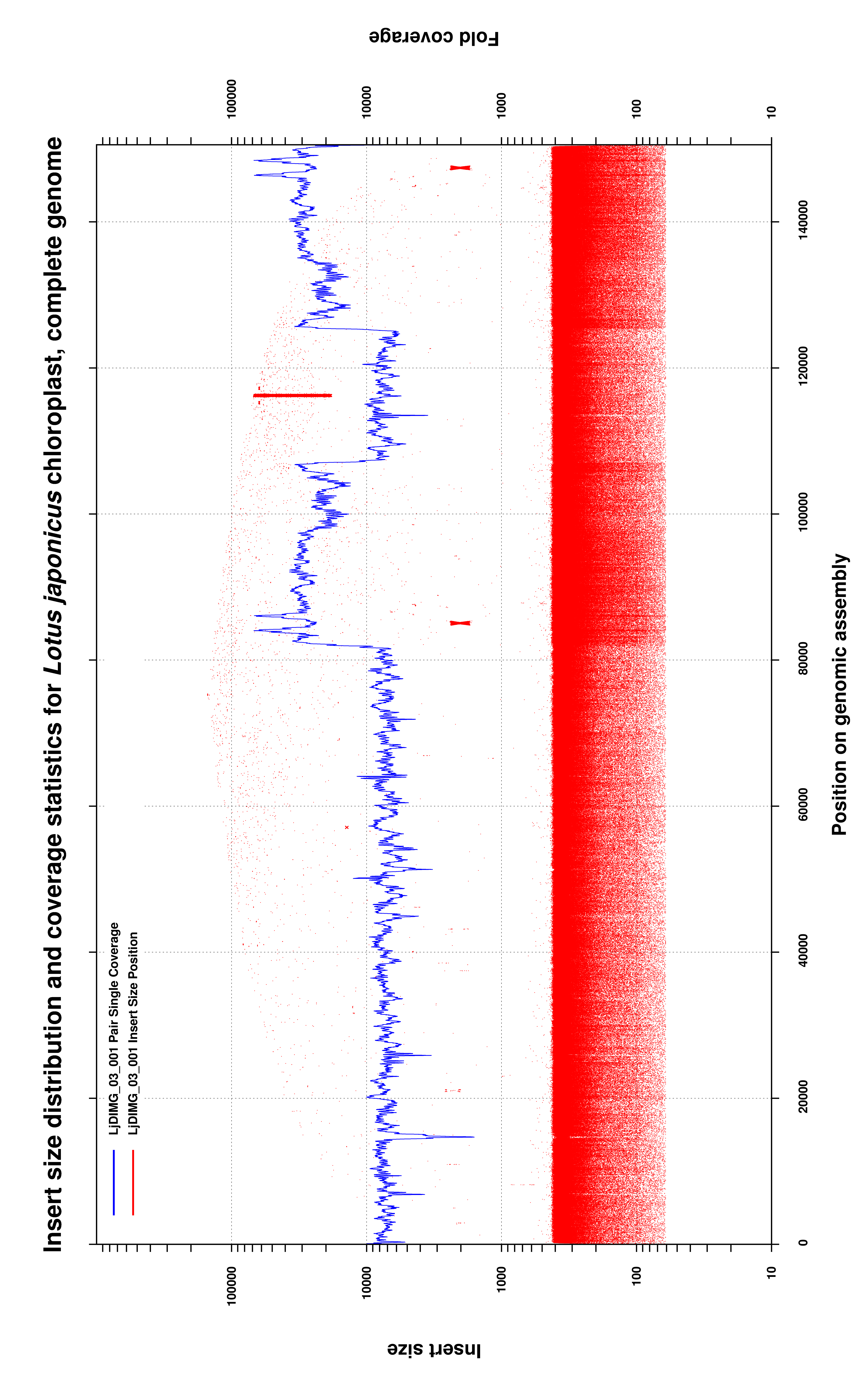

Supplement: Figure S4 — Coverage (blue line) and insert-size distribution (red dots) statistics generated during assembly of the L. japonicus cpDNA using the LjDIMG_03_001 insert library. Each dot represents the middle point between two paired reads. (TIF) [file pone.0051687.s004.tif]

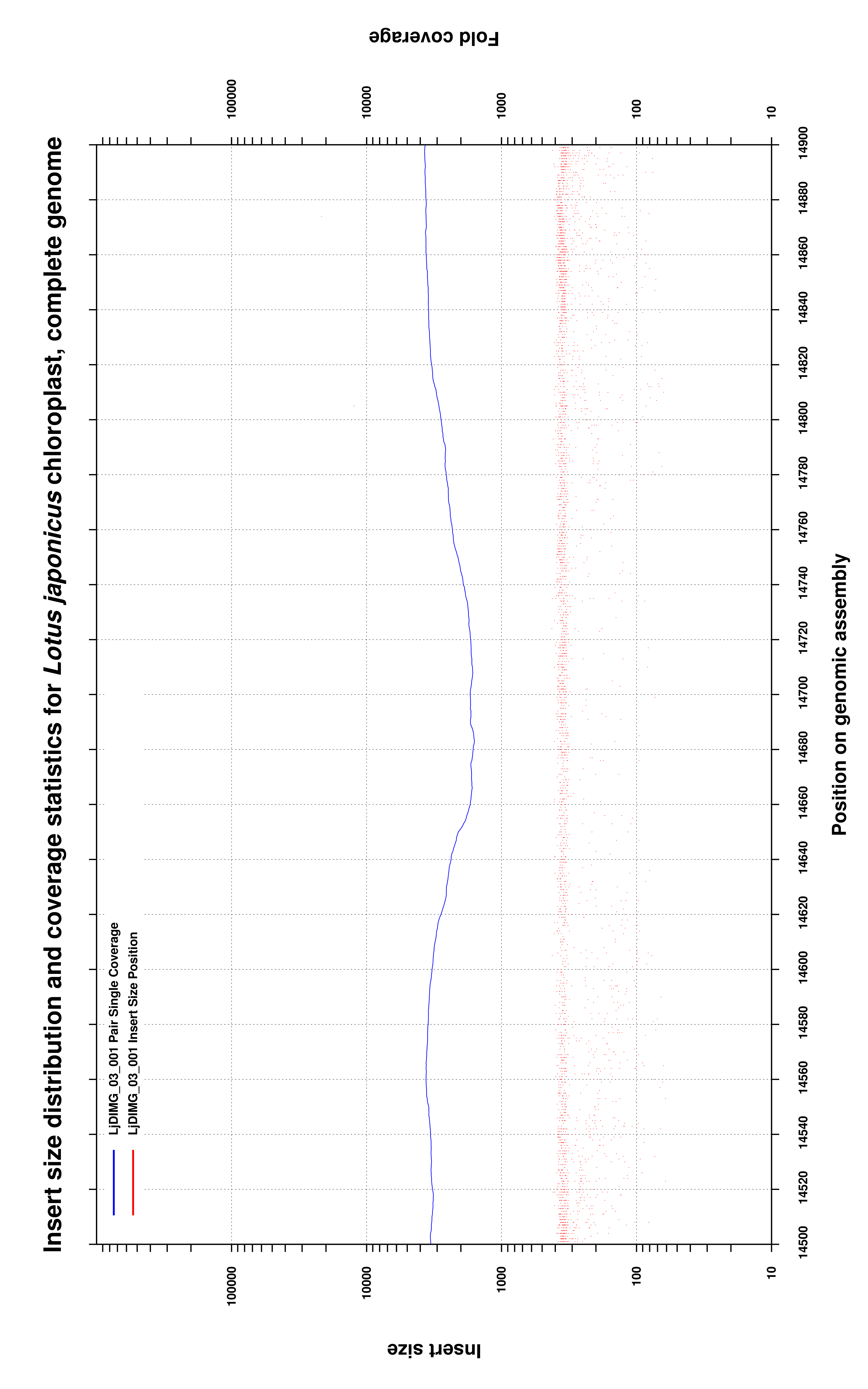

Supplement: Figure S5 — Coverage (blue line) and insert-size distribution (red dots) statistics similar to those described in Figure S4 of the site surrounding the L. japonicus chloroplast origin of replication. (TIF) [file pone.0051687.s005.tif]

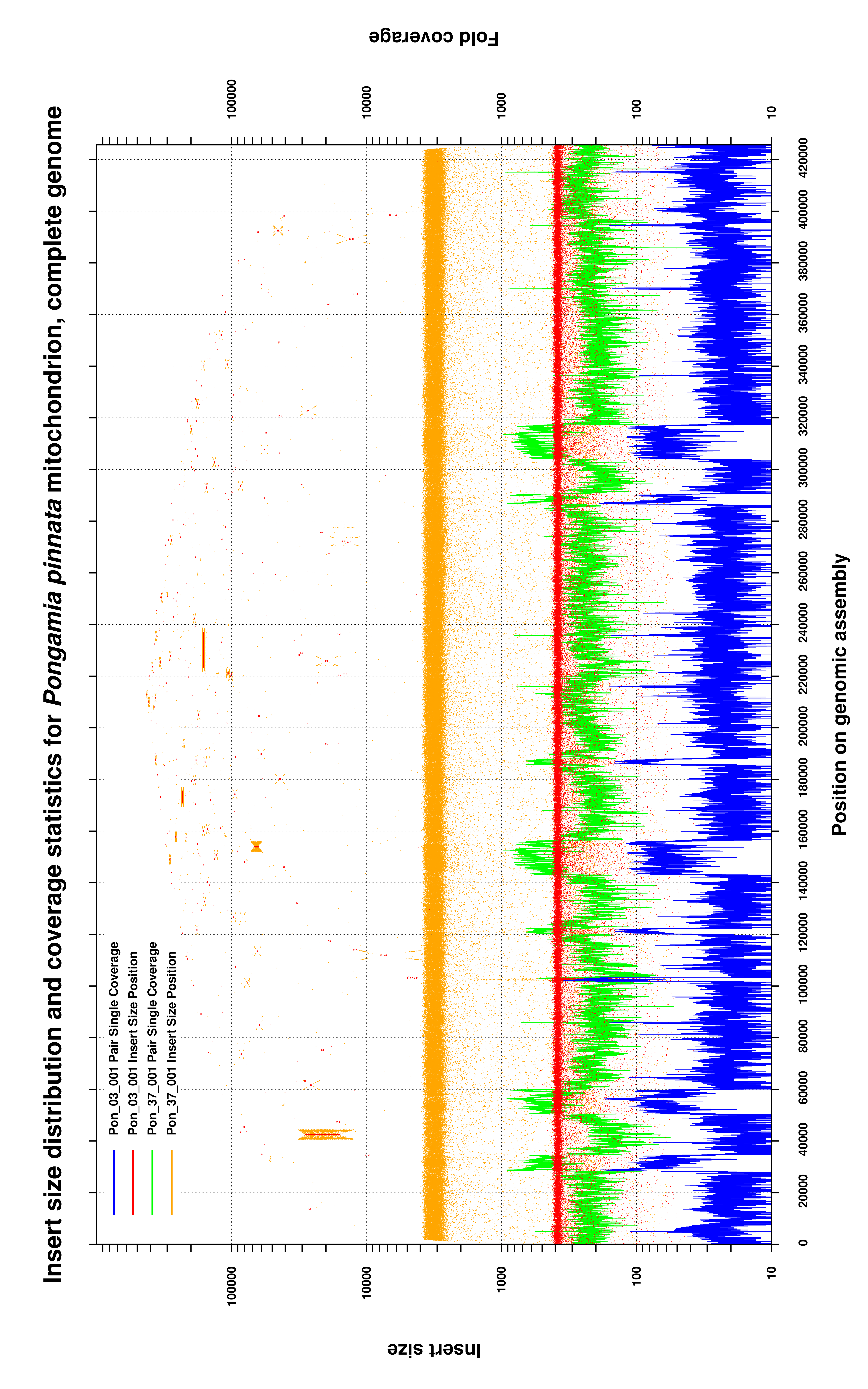

Supplement: Figure S6 — Coverage (blue and green lines) and insert-size distribution (red and yellow dots) statistics generated during assembly of the Pongamia mtDNA using the two insert libraries Pon_03_001 and Pon_37_001, respectively. Each dot represents the middle point between two paired-end or mate-pair reads. (TIF) [file pone.0051687.s006.tif]

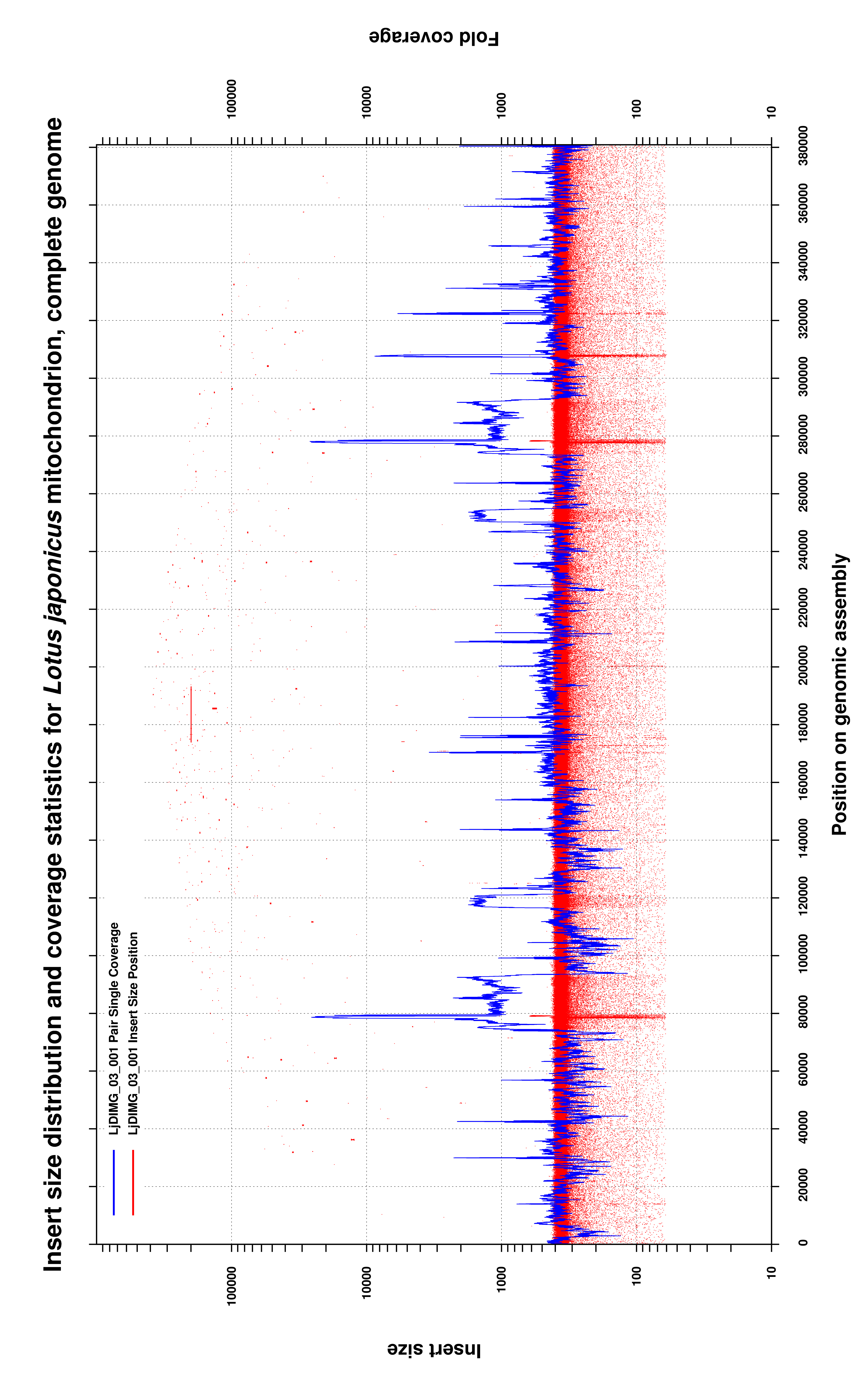

Supplement: Figure S7 — Coverage (blue line) and insert-size distribution (red dots) statistics generated during assembly of the L. japonicus mtDNA using the LjDIMG_03_001 insert library. Each dot represents the middle point between two paired reads. (TIF) [file pone.0051687.s007.tif]

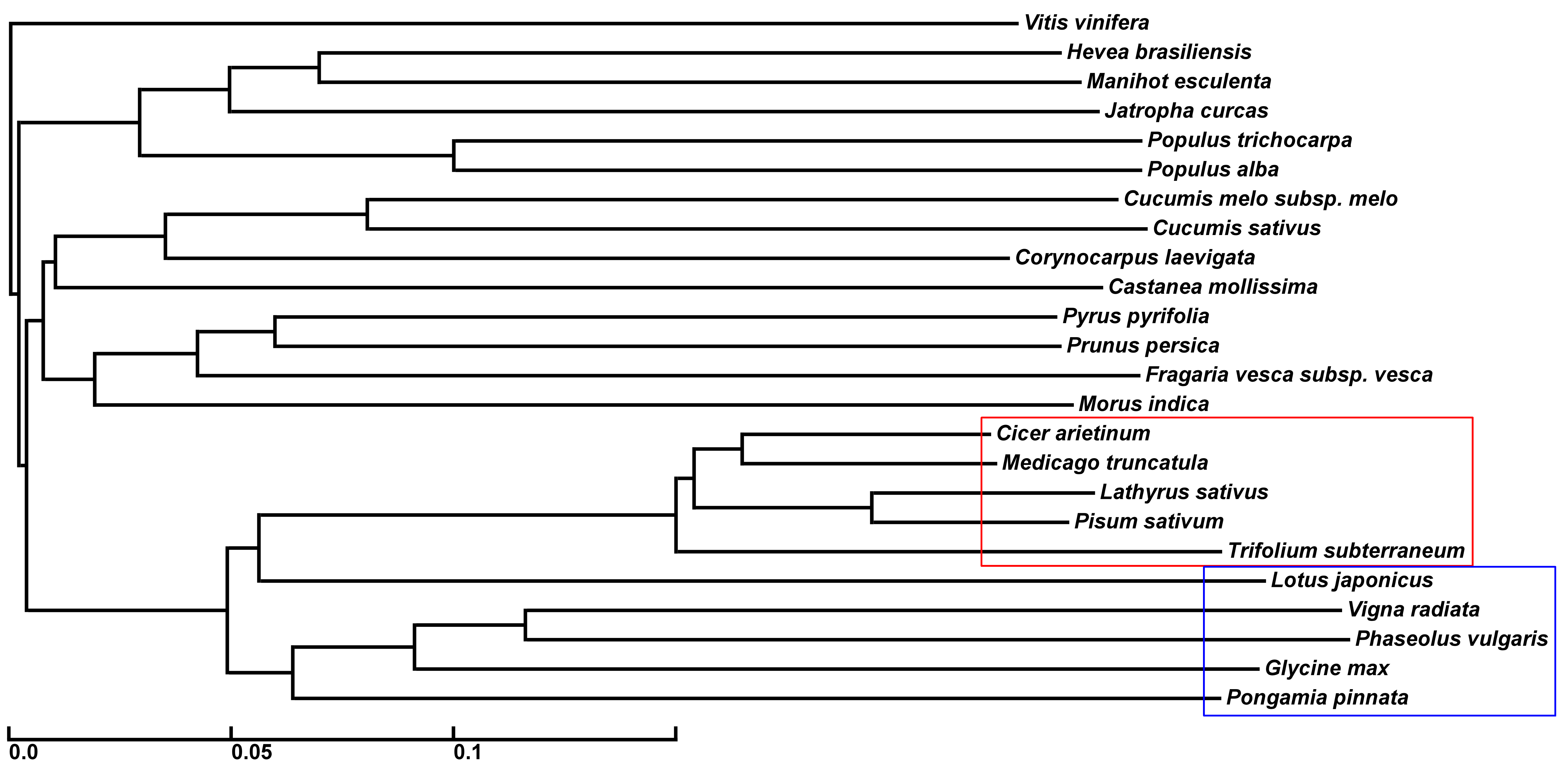

Supplement: Figure S8 — Rectangular phylogram of 23 legume-related cpDNA. Legume species boxed in red indicate the indeterminate (maintaining a nodule meristem and encoding Nodule Cysteine-Rich [NCR] peptides involved in bacteroid differentiation) nodulators, while those boxed in blue indicate the determinate (lacking a persistent meristem and NCR peptide genes) nodulators. These red and blue boxes also indicate the IRLC and non-IRLC legume clades, respectively. (TIF) [file pone.0051687.s008.tif]

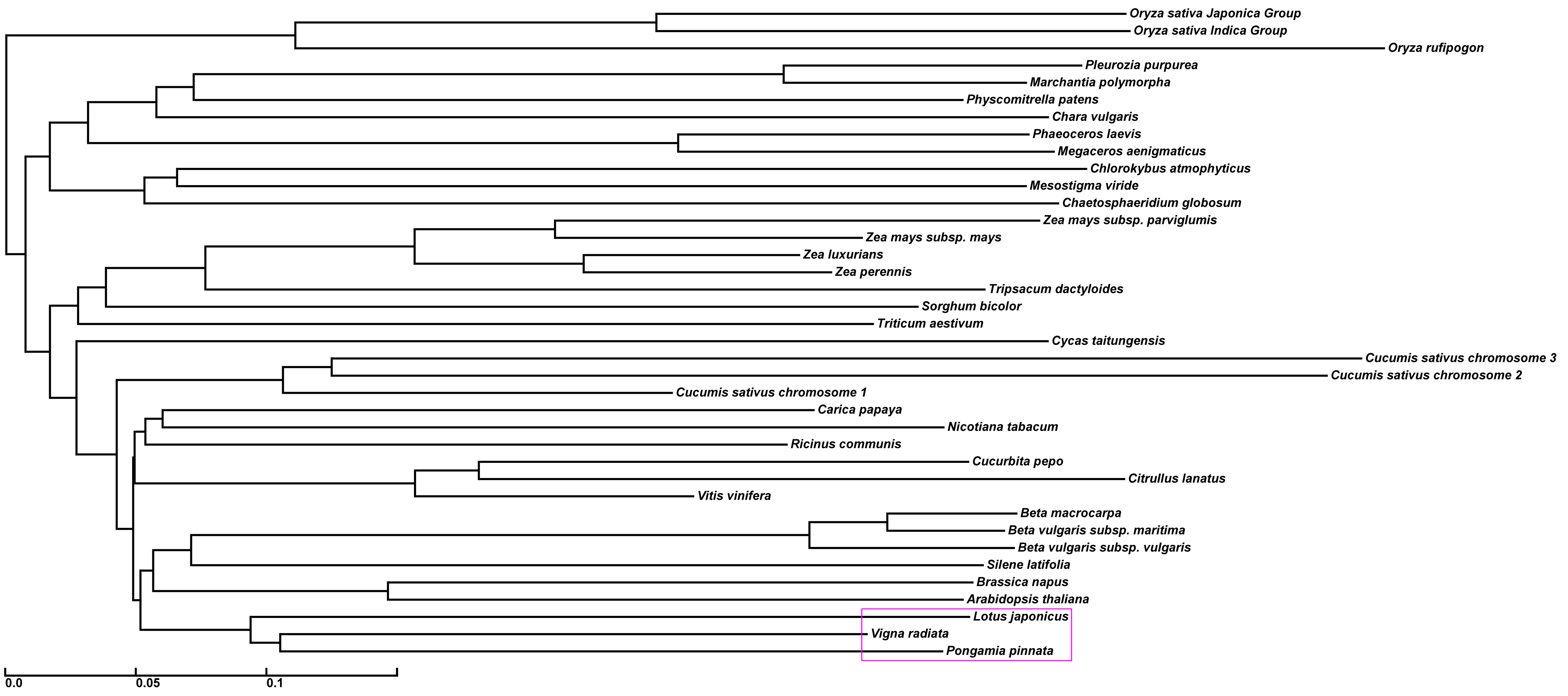

Supplement: Figure S9 — Rectangular phylogram of all 36 published mtDNA of species of Streptophyta. Three legume species have been boxed in pink. (TIF) [file pone.0051687.s009.tif]

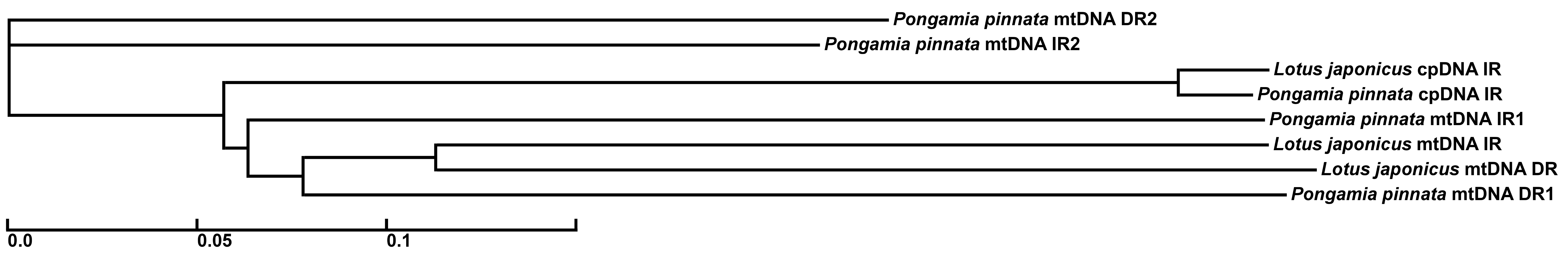

Supplement: Figure S10 — Rectangular phylogram of the Pongamia and L. japonicus cpDNA and mtDNA repeat regions. A high level of synteny between the inverted repeats of the cpDNA and low level of similarity amongst the mitochondrial repeats is noticeable. (TIF) [file pone.0051687.s010.tif]
