# Supplementary material for: Capturing the Biofuel Wellhead and Powerhouse: The Chloroplast and Mitochondrial Genomes of the Leguminous Feedstock Tree Pongamia pinnata
Source: PLoS One. 2012 Dec 14;7(12):e51687. doi: 10.1371/journal.pone.0051687 (PMC3522722; doi:10.1371/journal.pone.0051687)
Supplement: Table S1 — Codon usage, scored per thousand bp of all coding sequence, before (in parentheses) and after (in brackets) RNA editing of the L. japonicus (top), Pongamia (middle) and V. radiata (bottom) mtDNA (usage includes duplicated genes). In each of the three sub-tables, usage has been ranked in order of decreasing frequency, before putative RNA editing. (DOCX) [file pone.0051687.s011.docx]

**Table S1**

|  | **Amino acids** | | | **Codon usage** | | | | | | | | | | | | | | | | | |
| --- | --- | --- | --- | --- | --- | --- | --- | --- | --- | --- | --- | --- | --- | --- | --- | --- | --- | --- | --- | --- | --- |
|  |  |  |  | **1** | | | **2** | | | **3** | | | **4** | | | **5** | | | **6** | | |
| ***Lotus japonicus* mitochondria** | **A** | **Ala** | **Alanine** | **GCU** | (24.2) | [24.1] | **GCA** | (16.7) | [16.7] | **GCC** | (14.5) | [14.5] | **GCG** | (7.9) | [7.7] |  |  |  |  |  |  |
|  | **C** | **Cys** | **Cysteine** | **UGU** | (9.4) | [12.4] | **UGC** | (5.4) | [6.6] |  |  |  |  |  |  |  |  |  |  |  |  |
|  | **D** | **Asp** | **Aspartic acid** | **GAU** | (21.5) | [21.5] | **GAC** | (10.5) | [10.5] |  |  |  |  |  |  |  |  |  |  |  |  |
|  | **E** | **Glu** | **Glutamic acid** | **GAA** | (28.2) | [28.2] | **GAG** | (12.3) | [12.3] |  |  |  |  |  |  |  |  |  |  |  |  |
|  | **F** | **Phe** | **Phenylalanine** | **UUU** | (37.5) | [43.6] | **UUC** | (28.6) | [30.6] |  |  |  |  |  |  |  |  |  |  |  |  |
|  | **G** | **Gly** | **Glycine** | **GGA** | (25.4) | [25.4] | **GGU** | (21.6) | [21.6] | **GGG** | (11.2) | [11.2] | **GGC** | (9.5) | [9.5] |  |  |  |  |  |  |
|  | **H** | **His** | **Histidine** | **CAU** | (18.9) | [16.9] | **CAC** | (6.0) | [5.2] |  |  |  |  |  |  |  |  |  |  |  |  |
|  | **I** | **Ile** | **Isoleucine** | **AUU** | (33.0) | [33.8] | **AUA** | (22.9) | [23.5] | **AUC** | (19.7) | [19.2] |  |  |  |  |  |  |  |  |  |
|  | **K** | **Lys** | **Lysine** | **AAA** | (25.3) | [25.3] | **AAG** | (16.0) | [16.0] |  |  |  |  |  |  |  |  |  |  |  |  |
|  | **L** | **Leu** | **Leucine** | **UUA** | (25.2) | [32.6] | **CUU** | (21.6) | [23.2] | **UUG** | (20.2) | [24.8] | **CUA** | (15.9) | [19.3] | **CUC** | (11.2) | [11.9] | **CUG** | (10.6) | [13.3] |
|  | **M** | **Met** | **Methionine** | **AUG** | (27.2) | [27.8] |  |  |  |  |  |  |  |  |  |  |  |  |  |  |  |
|  | **N** | **Asn** | **Asparagine** | **AAU** | (22.1) | [22.1] | **AAC** | (10.5) | [10.5] |  |  |  |  |  |  |  |  |  |  |  |  |
|  | **P** | **Pro** | **Proline** | **CCU** | (20.2) | [15.3] | **CCA** | (15.5) | [10.9] | **CCC** | (10.8) | [8.4] | **CCG** | (8.9) | [5.6] |  |  |  |  |  |  |
|  | **Q** | **Gln** | **Glutamine** | **CAA** | (20.1) | [20.0] | **CAG** | (7.8) | [7.8] |  |  |  |  |  |  |  |  |  |  |  |  |
|  | **R** | **Arg** | **Arginine** | **AGA** | (15.9) | [15.9] | **CGA** | (15.7) | [15.5] | **CGU** | (14.5) | [11.5] | **CGG** | (9.2) | [5.5] | **AGG** | (7.5) | [7.5] | **CGC** | (7.4) | [6.1] |
|  | **S** | **Ser** | **Serine** | **UCU** | (20.7) | [19.0] | **UCA** | (18.0) | [11.7] | **AGU** | (15.4) | [15.4] | **UCC** | (14.9) | [13.6] | **UCG** | (14.3) | [10.2] | **AGC** | (9.2) | [9.2] |
|  | **T** | **Thr** | **Threonine** | **ACU** | (16.9) | [16.7] | **ACA** | (13.7) | [13.0] | **ACC** | (13.0) | [12.9] | **ACG** | (7.5) | [6.8] |  |  |  |  |  |  |
|  | **V** | **Val** | **Valine** | **GUU** | (19.3) | [19.5] | **GUA** | (17.6) | [17.7] | **GUC** | (12.7) | [12.6] | **GUG** | (12.6) | [12.9] |  |  |  |  |  |  |
|  | **W** | **Trp** | **Tryptophan** | **UGG** | (15.4) | [19.1] |  |  |  |  |  |  |  |  |  |  |  |  |  |  |  |
|  | **Y** | **Tyr** | **Tyrosine** | **UAU** | (23.5) | [25.6] | **UAC** | (7.8) | [8.5] |  |  |  |  |  |  |  |  |  |  |  |  |
|  |  |  | **STOP** | **UAA** | (1.5) | [1.6] | **UGA** | (0.8) | [1.0] | **UAG** | (0.7) | [0.7] |  |  |  |  |  |  |  |  |  |
|  |  | | | | | | | | | | | | | | | | | | | | |
| ***Pongamia pinnata* mitochondria** | **A** | **Ala** | **Alanine** | **GCU** | (24.1) | [24.0] | **GCA** | (15.7) | [15.6] | **GCC** | (14.6) | [14.6] | **GCG** | (8.3) | [8.0] |  |  |  |  |  |  |
|  | **C** | **Cys** | **Cysteine** | **UGU** | (8.7) | [11.2] | **UGC** | (4.8) | [5.9] |  |  |  |  |  |  |  |  |  |  |  |  |
|  | **D** | **Asp** | **Aspartic acid** | **GAU** | (22.8) | [22.8] | **GAC** | (11.0) | [11.0] |  |  |  |  |  |  |  |  |  |  |  |  |
|  | **E** | **Glu** | **Glutamic acid** | **GAA** | (29.4) | [29.4] | **GAG** | (13.5) | [13.5] |  |  |  |  |  |  |  |  |  |  |  |  |
|  | **F** | **Phe** | **Phenylalanine** | **UUU** | (35.7) | [41.2] | **UUC** | (27.0) | [29.1] |  |  |  |  |  |  |  |  |  |  |  |  |
|  | **G** | **Gly** | **Glycine** | **GGA** | (24.7) | [24.7] | **GGU** | (21.3) | [21.3] | **GGG** | (13.1) | [13.1] | **GGC** | (9.3) | [9.3] |  |  |  |  |  |  |
|  | **H** | **His** | **Histidine** | **CAU** | (17.3) | [15.6] | **CAC** | (6.4) | [5.7] |  |  |  |  |  |  |  |  |  |  |  |  |
|  | **I** | **Ile** | **Isoleucine** | **AUU** | (33.5) | [34.2] | **AUA** | (21.5) | [22.0] | **AUC** | (21.1) | [20.6] |  |  |  |  |  |  |  |  |  |
|  | **K** | **Lys** | **Lysine** | **AAA** | (27.9) | [27.9] | **AAG** | (16.0) | [16.0] |  |  |  |  |  |  |  |  |  |  |  |  |
|  | **L** | **Leu** | **Leucine** | **UUA** | (26.7) | [33.4] | **CUU** | (22.0) | [23.6] | **UUG** | (20.8) | [24.9] | **CUA** | (17.4) | [20.5] | **CUC** | (10.7) | [11.2] | **CUG** | (10.4) | [12.6] |
|  | **M** | **Met** | **Methionine** | **AUG** | (25.9) | [26.5] |  |  |  |  |  |  |  |  |  |  |  |  |  |  |  |
|  | **N** | **Asn** | **Asparagine** | **AAU** | (21.9) | [21.9] | **AAC** | (10.9) | [10.9] |  |  |  |  |  |  |  |  |  |  |  |  |
|  | **P** | **Pro** | **Proline** | **CCU** | (20.5) | [16.3] | **CCA** | (15.0) | [10.9] | **CCC** | (11.0) | [8.7] | **CCG** | (9.0) | [6.1] |  |  |  |  |  |  |
|  | **Q** | **Gln** | **Glutamine** | **CAA** | (21.5) | [21.4] | **CAG** | (7.3) | [7.3] |  |  |  |  |  |  |  |  |  |  |  |  |
|  | **R** | **Arg** | **Arginine** | **AGA** | (17.2) | [17.2] | **CGA** | (15.9) | [15.7] | **CGU** | (13.9) | [11.4] | **CGC** | (8.2) | [7.1] | **CGG** | (8.1) | [4.9] | **AGG** | (7.6) | [7.6] |
|  | **S** | **Ser** | **Serine** | **UCU** | (19.6) | [17.7] | **UCA** | (17.7) | [12.0] | **AGU** | (16.1) | [16.1] | **UCC** | (14.7) | [13.3] | **UCG** | (12.4) | [9.0] | **AGC** | (9.0) | [9.0] |
|  | **T** | **Thr** | **Threonine** | **ACU** | (17.3) | [17.0] | **ACA** | (13.7) | [13.3] | **ACC** | (12.5) | [12.4] | **ACG** | (6.8) | [6.2] |  |  |  |  |  |  |
|  | **V** | **Val** | **Valine** | **GUU** | (18.5) | [18.8] | **GUA** | (18.0) | [18.1] | **GUG** | (13.7) | [14.0] | **GUC** | (11.9) | [11.7] |  |  |  |  |  |  |
|  | **W** | **Trp** | **Tryptophan** | **UGG** | (13.8) | [16.9] |  |  |  |  |  |  |  |  |  |  |  |  |  |  |  |
|  | **Y** | **Tyr** | **Tyrosine** | **UAU** | (24.4) | [26.3] | **UAC** | (7.6) | [8.2] |  |  |  |  |  |  |  |  |  |  |  |  |
|  |  |  | **STOP** | **UAA** | (1.6) | [1.7] | **UGA** | (0.9) | [1.1] | **UAG** | (0.5) | [0.5] |  |  |  |  |  |  |  |  |  |
|  |  | | | | | | | | | | | | | | | | | | | | |
| ***Vigna radiata* mitochondria** | **A** | **Ala** | **Alanine** | **GCU** | (24.5) | [24.4] | **GCA** | (16.4) | [16.3] | **GCC** | (15.4) | [15.4] | **GCG** | (8.0) | [7.7] |  |  |  |  |  |  |
|  | **C** | **Cys** | **Cysteine** | **UGU** | (9.1) | [11.8] | **UGC** | (4.9) | [6.1] |  |  |  |  |  |  |  |  |  |  |  |  |
|  | **D** | **Asp** | **Aspartic acid** | **GAU** | (21.4) | [21.4] | **GAC** | (9.7) | [9.7] |  |  |  |  |  |  |  |  |  |  |  |  |
|  | **E** | **Glu** | **Glutamic acid** | **GAA** | (28.4) | [28.4] | **GAG** | (12.3) | [12.3] |  |  |  |  |  |  |  |  |  |  |  |  |
|  | **F** | **Phe** | **Phenylalanine** | **UUU** | (37.3) | [43.6] | **UUC** | (28.8) | [30.7] |  |  |  |  |  |  |  |  |  |  |  |  |
|  | **G** | **Gly** | **Glycine** | **GGA** | (25.8) | [25.8] | **GGU** | (22.7) | [22.7] | **GGG** | (11.7) | [11.7] | **GGC** | (9.7) | [9.7] |  |  |  |  |  |  |
|  | **H** | **His** | **Histidine** | **CAU** | (18.1) | [16.2] | **CAC** | (6.0) | [5.2] |  |  |  |  |  |  |  |  |  |  |  |  |
|  | **I** | **Ile** | **Isoleucine** | **AUU** | (33.0) | [33.8] | **AUA** | (22.7) | [23.3] | **AUC** | (21.0) | [20.6] |  |  |  |  |  |  |  |  |  |
|  | **K** | **Lys** | **Lysine** | **AAA** | (26.3) | [26.3] | **AAG** | (17.0) | [17.0] |  |  |  |  |  |  |  |  |  |  |  |  |
|  | **L** | **Leu** | **Leucine** | **UUA** | (26.2) | [33.4] | **CUU** | (21.9) | [23.5] | **UUG** | (21.0) | [25.3] | **CUA** | (16.4) | [19.4] | **CUC** | (10.9) | [11.7] | **CUG** | (10.2) | [12.6] |
|  | **M** | **Met** | **Methionine** | **AUG** | (27.7) | [28.2] |  |  |  |  |  |  |  |  |  |  |  |  |  |  |  |
|  | **N** | **Asn** | **Asparagine** | **AAU** | (21.5) | [21.5] | **AAC** | (10.7) | [10.7] |  |  |  |  |  |  |  |  |  |  |  |  |
|  | **P** | **Pro** | **Proline** | **CCU** | (20.5) | [16.2] | **CCA** | (14.3) | [10.1] | **CCC** | (11.7) | [9.2] | **CCG** | (8.6) | [5.4] |  |  |  |  |  |  |
|  | **Q** | **Gln** | **Glutamine** | **CAA** | (19.9) | [19.8] | **CAG** | (6.6) | [6.6] |  |  |  |  |  |  |  |  |  |  |  |  |
|  | **R** | **Arg** | **Arginine** | **AGA** | (16.2) | [16.2] | **CGA** | (15.2) | [14.9] | **CGU** | (13.4) | [10.8] | **CGG** | (7.9) | [4.9] | **AGG** | (7.9) | [7.9] | **CGC** | (7.3) | [6.1] |
|  | **S** | **Ser** | **Serine** | **UCU** | (21.0) | [18.6] | **UCA** | (18.5) | [12.4] | **AGU** | (15.5) | [15.5] | **UCC** | (14.9) | [13.4] | **UCG** | (12.9) | [9.4] | **AGC** | (9.0) | [9.0] |
|  | **T** | **Thr** | **Threonine** | **ACU** | (16.6) | [16.2] | **ACA** | (14.2) | [13.6] | **ACC** | (13.0) | [12.9] | **ACG** | (7.2) | [6.7] |  |  |  |  |  |  |
|  | **V** | **Val** | **Valine** | **GUU** | (19.1) | [19.4] | **GUA** | (17.3) | [17.4] | **GUG** | (14.3) | [14.6] | **GUC** | (12.1) | [11.9] |  |  |  |  |  |  |
|  | **W** | **Trp** | **Tryptophan** | **UGG** | (14.6) | [17.6] |  |  |  |  |  |  |  |  |  |  |  |  |  |  |  |
|  | **Y** | **Tyr** | **Tyrosine** | **UAU** | (22.9) | [24.9] | **UAC** | (7.9) | [8.5] |  |  |  |  |  |  |  |  |  |  |  |  |
|  |  |  | **STOP** | **UAA** | (1.3) | [1.4] | **UGA** | (1.0) | [1.3] | **UAG** | (0.5) | [0.5] |  |  |  |  |  |  |  |  |  |
