# Supplementary material for: Capturing the Biofuel Wellhead and Powerhouse: The Chloroplast and Mitochondrial Genomes of the Leguminous Feedstock Tree Pongamia pinnata
Source: PLoS One. 2012 Dec 14;7(12):e51687. doi: 10.1371/journal.pone.0051687 (PMC3522722; doi:10.1371/journal.pone.0051687)
Supplement: Table S2 — Codon usage, scored per thousand bp of all coding sequences of the Pongamia chloroplast (including duplicated genes). Usage has been ranked as described in Table S1. (DOCX) [file pone.0051687.s012.docx]

**Table S2**

|  | **Amino acids** | | | **Codon usage** | | | | | | | | | | | |
| --- | --- | --- | --- | --- | --- | --- | --- | --- | --- | --- | --- | --- | --- | --- | --- |
|  |  |  |  | **1** | | **2** | | **3** | | **4** | | **5** | | **6** | |
| ***Pongamia pinnata* chloroplast** | **A** | **Ala** | **Alanine** | **GCU** | 24.0 | **GCA** | 14.6 | **GCC** | 7.3 | **GCG** | 4.6 |  |  |  |  |
|  | **C** | **Cys** | **Cysteine** | **UGU** | 9.3 | **UGC** | 3.1 |  |  |  |  |  |  |  |  |
|  | **D** | **Asp** | **Aspartic acid** | **GAU** | 32.6 | **GAC** | 6.7 |  |  |  |  |  |  |  |  |
|  | **E** | **Glu** | **Glutamic acid** | **GAA** | 39.7 | **GAG** | 11.3 |  |  |  |  |  |  |  |  |
|  | **F** | **Phe** | **Phenylalanine** | **UUU** | 42.5 | **UUC** | 18.7 |  |  |  |  |  |  |  |  |
|  | **G** | **Gly** | **Glycine** | **GGA** | 28.0 | **GGU** | 22.8 | **GGG** | 9.6 | **GGC** | 4.9 |  |  |  |  |
|  | **H** | **His** | **Histidine** | **CAU** | 18.9 | **CAC** | 4.7 |  |  |  |  |  |  |  |  |
|  | **I** | **Ile** | **Isoleucine** | **AUU** | 46.6 | **AUA** | 31.8 | **AUC** | 15.3 |  |  |  |  |  |  |
|  | **K** | **Lys** | **Lysine** | **AAA** | 45.5 | **AAG** | 13.1 |  |  |  |  |  |  |  |  |
|  | **L** | **Leu** | **Leucine** | **UUA** | 35.3 | **UUG** | 21.3 | **CUU** | 21.0 | **CUA** | 13.9 | **CUG** | 6.1 | **CUC** | 6.0 |
|  | **M** | **Met** | **Methionine** | **AUG** | 23.0 |  |  |  |  |  |  |  |  |  |  |
|  | **N** | **Asn** | **Asparagine** | **AAU** | 41.0 | **AAC** | 10.6 |  |  |  |  |  |  |  |  |
|  | **P** | **Pro** | **Proline** | **CCU** | 15.6 | **CCA** | 11.9 | **CCC** | 6.8 | **CCG** | 4.4 |  |  |  |  |
|  | **Q** | **Gln** | **Glutamine** | **CAA** | 28.4 | **CAG** | 7.4 |  |  |  |  |  |  |  |  |
|  | **R** | **Arg** | **Arginine** | **AGA** | 17.8 | **CGA** | 13.2 | **CGU** | 12.9 | **AGG** | 5.4 | **CGG** | 4.0 | **CGC** | 3.1 |
|  | **S** | **Ser** | **Serine** | **UCU** | 21.0 | **UCA** | 16.9 | **AGU** | 15.1 | **UCC** | 11.6 | **UCG** | 7.1 | **AGC** | 4.3 |
|  | **T** | **Thr** | **Threonine** | **ACU** | 21.3 | **ACA** | 15.7 | **ACC** | 7.8 | **ACG** | 5.0 |  |  |  |  |
|  | **V** | **Val** | **Valine** | **GUA** | 20.3 | **GUU** | 19.2 | **GUG** | 6.1 | **GUC** | 5.6 |  |  |  |  |
|  | **W** | **Trp** | **Tryptophan** | **UGG** | 17.3 |  |  |  |  |  |  |  |  |  |  |
|  | **Y** | **Tyr** | **Tyrosine** | **UAU** | 31.1 | **UAC** | 6.8 |  |  |  |  |  |  |  |  |
|  |  |  | **STOP** | **UAA** | 1.9 | **UGA** | 0.7 | **UAG** | 0.7 |  |  |  |  |  |  |
