# Supplementary material for: Capturing the Biofuel Wellhead and Powerhouse: The Chloroplast and Mitochondrial Genomes of the Leguminous Feedstock Tree Pongamia pinnata
Source: PLoS One. 2012 Dec 14;7(12):e51687. doi: 10.1371/journal.pone.0051687 (PMC3522722; doi:10.1371/journal.pone.0051687)
Supplement: Table S3 — Table of potential RNA editing sites in a list of seventy-seven unique Pongamia chloroplast genes. Positions of these sites refer to the position after the coding sequences’ translational start site. In the table header, an “X” represents the original base (before RNA editing) and a “Y” indicates the change (after RNA editing). An allele balance ratio (ABR) is then listed alongside the evidence supporting the presence of the SNP or indel. A filter of ABR > = 0.40 and coverage > = 3 was applied to these values in an attempt to remove a number of false positives. (DOCX) [file pone.0051687.s013.docx]

**Table S3**

| **Gene** | **Pos** | **X** | **Y** | **ABR** | **A** | **C** | **G** | **T** | **N** | **Del** | **Ins** |
| --- | --- | --- | --- | --- | --- | --- | --- | --- | --- | --- | --- |
| ***psbA*** | 464 | C | A | 0.435 | 193 | 251 | 0 | 2 | 0 | 0 | A2C1 |
| ***psbA*** | 474 | C | T | 0.495 | 0 | 212 | 0 | 208 | 0 | 2 | 0 |
| ***psbA*** | 477 | G | T | 0.465 | 0 | 0 | 230 | 200 | 0 | 1 | 0 |
| ***matK*** | 116 | G | T | 0.870 | 0 | 0 | 6 | 40 | 0 | 0 | 0 |
| ***matK*** | 118 | T | - | 0.851 | 0 | 0 | 0 | 7 | 0 | 40 | 0 |
| ***matK*** | 120 | C | G | 0.851 | 0 | 7 | 40 | 0 | 0 | 0 | 0 |
| ***matK*** | 121 | A | AG | 0.816 | 49 | 0 | 0 | 0 | 0 | 0 | G40 |
| ***rbcL*** | 853 | A | C | 1.000 | 0 | 912 | 0 | 0 | 0 | 0 | 0 |
| ***atpE*** | 335 | T | C | 0.997 | 47 | 6582 | 5 | 19 | 0 | 1 | C1 |
| ***ndhC*** | 201 | A | C | 0.999 | 4 | 2884 | 1 | 7 | 0 | 0 | 0 |
| ***ndhC*** | 323 | C | T | 0.816 | 1 | 495 | 0 | 2202 | 0 | 1 | T1 |
| ***ycf3*** | 126 | T | G | 0.742 | 0 | 1 | 432 | 150 | 0 | 1 | 0 |
| ***psaB*** | 417 | C | T | 0.996 | 0 | 2 | 0 | 548 | 0 | 1 | 0 |
| ***psbC*** | 632 | G | C | 0.998 | 2 | 2100 | 4 | 4 | 1 | 0 | 0 |
| ***psbD*** | 911 | T | C | 0.511 | 4 | 278 | 14 | 266 | 0 | 1 | C14G2 |
| ***psbD*** | 918 | A | G | 0.475 | 254 | 4 | 230 | 1 | 0 | 1 | G2 |
| ***psbD*** | 920 | C | G | 0.426 | 4 | 302 | 224 | 6 | 0 | 0 | T1G1 |
| ***rpoB*** | 156 | A | - | 0.540 | 29 | 0 | 0 | 0 | 0 | 34 | 0 |
| ***rpoB*** | 163 | G | - | 0.586 | 2 | 0 | 22 | 0 | 0 | 34 | 0 |
| ***rpoB*** | 164 | A | - | 0.586 | 24 | 0 | 0 | 0 | 0 | 34 | 0 |
| ***rpoB*** | 167 | C | CT | 0.576 | 0 | 59 | 0 | 0 | 0 | 0 | T34 |
| ***rpoB*** | 172 | C | G | 0.630 | 0 | 20 | 34 | 0 | 0 | 0 | 0 |
| ***rpoB*** | 338 | C | T | 0.786 | 0 | 15 | 0 | 55 | 0 | 0 | 0 |
| ***rpoB*** | 551 | C | T | 0.600 | 0 | 8 | 0 | 12 | 0 | 0 | 0 |
| ***rpoB*** | 566 | C | T | 0.588 | 0 | 7 | 0 | 10 | 0 | 0 | 0 |
| ***rpoB*** | 1858 | G | A | 1.000 | 111 | 0 | 0 | 0 | 0 | 0 | 0 |
| ***rpoB*** | 2018 | C | T | 0.680 | 0 | 16 | 0 | 34 | 0 | 0 | 0 |
| ***rpoB*** | 2040 | T | C | 0.486 | 0 | 17 | 0 | 18 | 0 | 0 | 0 |
| ***rpoB*** | 2043 | A | G | 0.515 | 16 | 0 | 17 | 0 | 0 | 0 | 0 |
| ***rpoB*** | 2068 | A | - | 0.486 | 18 | 0 | 0 | 0 | 0 | 17 | 0 |
| ***rpoB*** | 2484 | T | G | 0.727 | 2 | 2 | 32 | 12 | 0 | 0 | 0 |
| ***rpoB*** | 2496 | T | A | 0.564 | 22 | 0 | 1 | 17 | 0 | 0 | 0 |
| ***rpoC1*** | 41 | C | T | 0.789 | 0 | 4 | 0 | 15 | 0 | 0 | 0 |
| ***rpoC1*** | 946 | G | A | 1.000 | 387 | 0 | 0 | 0 | 0 | 0 | 0 |
| ***rpoC1*** | 1344 | G | A | 0.992 | 258 | 1 | 2 | 1 | 0 | 0 | 0 |
| ***rpoC2*** | 1708 | G | A | 1.000 | 106 | 0 | 0 | 0 | 0 | 0 | 0 |
| ***rpoC2*** | 3731 | C | T | 0.941 | 4 | 334 | 14 | 5284 | 1 | 9 | 0 |
| ***rps2*** | 134 | C | T | 0.956 | 1 | 72 | 1 | 1566 | 0 | 0 | 0 |
| ***rps2*** | 248 | C | T | 0.924 | 10 | 405 | 10 | 4909 | 0 | 1 | 0 |
| ***atpF*** | 92 | C | T | 0.890 | 0 | 253 | 6 | 2043 | 0 | 0 | 0 |
| ***atpA*** | 531 | T | G | 0.999 | 2 | 1 | 4467 | 3 | 0 | 0 | 0 |
| ***atpA*** | 791 | C | T | 0.961 | 1 | 59 | 0 | 1472 | 0 | 0 | 0 |
| ***atpA*** | 1358 | G | A | 1.000 | 839 | 0 | 0 | 2 | 0 | 0 | 0 |
| ***accD*** | 594 | G | T | 1.000 | 0 | 0 | 0 | 34 | 0 | 0 | 0 |
| ***accD*** | 1475 | C | T | 0.637 | 4 | 29 | 0 | 51 | 0 | 0 | A1 |
| ***accD*** | 1478 | T | TC | 0.729 | 0 | 0 | 0 | 70 | 0 | 0 | C51 |
| ***accD*** | 1479 | G | A | 0.850 | 51 | 0 | 9 | 0 | 0 | 0 | 0 |
| ***accD*** | 1483 | C | T | 0.962 | 0 | 2 | 0 | 51 | 0 | 0 | 0 |
| ***accD*** | 1484 | A | C | 0.962 | 2 | 51 | 0 | 0 | 0 | 0 | 0 |
| ***accD*** | 1489 | G | A | 0.981 | 51 | 0 | 1 | 0 | 0 | 0 | 0 |
| ***rps16*** | 36 | G | C | 1.000 | 1 | 3 | 0 | 0 | 0 | 0 | 0 |
| ***rps16*** | 40 | C | T | 1.000 | 0 | 0 | 0 | 480 | 0 | 0 | 0 |
| ***rps16*** | 212 | C | T | 0.537 | 5 | 204 | 0 | 237 | 0 | 0 | A3 |
| ***rps18*** | 221 | C | T | 0.577 | 0 | 529 | 1 | 722 | 0 | 0 | 0 |
| ***clpP*** | 559 | C | T | 0.605 | 16 | 708 | 2 | 1084 | 0 | 0 | 0 |
| ***psbH*** | 161 | T | - | 0.480 | 0 | 0 | 0 | 92 | 0 | 85 | 0 |
| ***psbH*** | 166 | A | T | 0.494 | 87 | 0 | 0 | 85 | 0 | 0 | 0 |
| ***psbH*** | 169 | A | T | 0.497 | 86 | 0 | 0 | 85 | 0 | 0 | 0 |
| ***psbH*** | 170 | T | A | 0.497 | 85 | 0 | 0 | 86 | 0 | 0 | 0 |
| ***psbH*** | 171 | T | G | 0.497 | 0 | 0 | 85 | 86 | 0 | 0 | 0 |
| ***psbH*** | 177 | G | A | 0.512 | 84 | 0 | 80 | 1 | 0 | 0 | 0 |
| ***psbH*** | 178 | A | C | 0.535 | 74 | 85 | 0 | 0 | 0 | 0 | 0 |
| ***petB*** | 386 | T | C | 0.998 | 1 | 589 | 0 | 1 | 0 | 0 | 0 |
| ***petB*** | 611 | C | T | 0.820 | 0 | 52 | 0 | 237 | 0 | 0 | 0 |
| ***petD*** | 5 | G | C | 0.504 | 0 | 66 | 65 | 0 | 0 | 0 | 0 |
| ***petD*** | 7 | G | A | 0.503 | 83 | 0 | 82 | 0 | 0 | 0 | 0 |
| ***petD*** | 482 | A | T | 1.000 | 0 | 1 | 0 | 3 | 0 | 0 | 0 |
| ***petD*** | 483 | A | T | 1.000 | 0 | 0 | 0 | 3 | 0 | 0 | 0 |
| ***rpoA*** | 200 | C | T | 0.492 | 1 | 874 | 1 | 848 | 0 | 0 | 0 |
| ***rpoA*** | 858 | T | A | 0.710 | 125 | 1 | 0 | 51 | 0 | 0 | 0 |
| ***rpoA*** | 862 | T | A | 0.698 | 125 | 0 | 0 | 54 | 0 | 0 | 0 |
| ***rpoA*** | 865 | C | - | 0.722 | 0 | 49 | 0 | 0 | 0 | 127 | 0 |
| ***rpoA*** | 866 | T | - | 0.710 | 0 | 0 | 0 | 51 | 0 | 125 | 0 |
| ***rpoA*** | 867 | C | - | 0.714 | 0 | 50 | 0 | 0 | 0 | 125 | 0 |
| ***rps11*** | 412 | T | A | 0.600 | 6 | 3 | 0 | 4 | 0 | 0 | 0 |
| ***rpl36*** | 112 | T | C | 0.833 | 1 | 5 | 0 | 1 | 0 | 0 | 0 |
| ***rpl23*** | 71 | C | T | 0.969 | 0 | 58 | 0 | 1808 | 0 | 0 | 0 |
| ***rpl23*** | 89 | C | T | 0.816 | 1 | 304 | 0 | 1347 | 0 | 0 | 0 |
| ***ycf2*** | 1407 | G | A | 0.607 | 17 | 0 | 11 | 0 | 0 | 0 | 0 |
| ***ycf2*** | 1413 | G | T | 0.600 | 0 | 0 | 8 | 12 | 0 | 0 | 0 |
| ***ycf2*** | 1420 | A | G | 0.462 | 7 | 0 | 6 | 0 | 0 | 0 | 0 |
| ***ycf2*** | 1462 | C | T | 0.643 | 0 | 10 | 0 | 18 | 0 | 0 | 0 |
| ***ycf2*** | 1463 | C | T | 0.630 | 0 | 10 | 1 | 17 | 0 | 0 | 0 |
| ***ycf2*** | 1472 | C | T | 0.714 | 1 | 46 | 0 | 115 | 0 | 0 | 0 |
| ***ycf2*** | 1914 | G | T | 1.000 | 0 | 0 | 0 | 8 | 0 | 0 | 0 |
| ***ycf2*** | 5573 | T | A | 0.667 | 28 | 2 | 4 | 14 | 1 | 1 | 0 |
| ***ycf2*** | 5575 | C | A | 0.855 | 71 | 12 | 0 | 1 | 0 | 2 | 0 |
| ***ndhB*** | 149 | C | T | 0.714 | 0 | 8 | 0 | 20 | 0 | 0 | 0 |
| ***ndhB*** | 467 | C | T | 0.790 | 0 | 41 | 0 | 154 | 1 | 0 | 0 |
| ***ndhB*** | 542 | C | T | 0.839 | 1 | 119 | 0 | 620 | 0 | 0 | 0 |
| ***ndhB*** | 746 | C | T | 0.697 | 0 | 95 | 0 | 219 | 0 | 0 | 0 |
| ***ndhB*** | 830 | C | T | 0.622 | 0 | 48 | 0 | 79 | 0 | 0 | 0 |
| ***ndhB*** | 836 | C | T | 0.786 | 0 | 24 | 0 | 88 | 0 | 0 | 0 |
| ***ndhB*** | 1112 | C | T | 0.820 | 0 | 55 | 1 | 250 | 0 | 0 | 0 |
| ***ndhB*** | 1255 | C | T | 0.842 | 0 | 76 | 0 | 405 | 0 | 0 | 0 |
| ***ndhB*** | 1481 | C | T | 0.804 | 0 | 47 | 0 | 193 | 0 | 0 | 0 |
| ***ndhF*** | 552 | A | T | 0.400 | 6 | 0 | 1 | 4 | 0 | 0 | 0 |
| ***ndhF*** | 1975 | A | C | 1.000 | 0 | 13 | 0 | 0 | 0 | 0 | 0 |
| ***ndhD*** | 585 | C | A | 1.000 | 401 | 0 | 0 | 3 | 0 | 0 | 0 |
| ***ndhD*** | 674 | C | T | 0.576 | 0 | 266 | 0 | 362 | 0 | 0 | 0 |
| ***ndhD*** | 878 | C | T | 0.496 | 0 | 116 | 0 | 114 | 0 | 0 | 0 |
| ***ndhE*** | 233 | C | T | 0.751 | 1 | 52 | 1 | 157 | 0 | 0 | 0 |
| ***ndhG*** | 346 | A | C | 0.999 | 1 | 709 | 0 | 0 | 0 | 0 | 0 |
| ***ndhG*** | 463 | C | T | 0.992 | 1 | 2 | 0 | 260 | 0 | 1 | 0 |
| ***ndhA*** | 35 | A | AT | 0.438 | 206 | 5 | 1 | 60 | 0 | 0 | T119 |
| ***ndhA*** | 344 | C | T | 0.536 | 1 | 621 | 0 | 717 | 0 | 1 | 0 |
| ***ndhA*** | 1014 | A | G | 0.803 | 13 | 0 | 53 | 0 | 0 | 0 | 0 |
| ***ndhA*** | 1022 | G | A | 0.818 | 54 | 0 | 12 | 0 | 0 | 0 | 0 |
| ***ndhA*** | 1024 | T | - | 0.831 | 0 | 0 | 0 | 11 | 0 | 54 | 0 |
| ***ndhA*** | 1025 | G | - | 0.831 | 0 | 0 | 11 | 0 | 0 | 54 | 0 |
| ***ndhA*** | 1028 | A | AC | 0.815 | 65 | 0 | 0 | 0 | 0 | 0 | C53 |
| ***ndhA*** | 1029 | G | T | 0.828 | 0 | 1 | 11 | 53 | 0 | 0 | 0 |
| ***ndhH*** | 505 | C | T | 0.729 | 0 | 161 | 2 | 433 | 0 | 2 | 0 |
| ***ycf1*** | 2103 | A | G | 0.997 | 4 | 1 | 1353 | 0 | 0 | 11 | 0 |
| ***ycf1*** | 3384 | C | T | 1.000 | 0 | 0 | 0 | 31 | 0 | 0 | 0 |
| ***ycf1*** | 3722 | T | C | 0.996 | 1 | 2416 | 0 | 10 | 0 | 0 | 0 |
| ***ycf1*** | 3757 | C | T | 0.999 | 1 | 3 | 1 | 3417 | 0 | 2 | 0 |
| ***ycf1*** | 3962 | A | AG | 0.433 | 244 | 1 | 1 | 1 | 0 | 0 | G107 |
| ***ycf1*** | 3966 | T | TT | 0.420 | 3 | 0 | 0 | 252 | 0 | 0 | T107 |
| ***ycf1*** | 3967 | T | TA | 0.437 | 0 | 0 | 0 | 245 | 0 | 0 | A107 |
| ***ycf1*** | 4261 | T | A | 0.991 | 324 | 0 | 1 | 3 | 0 | 0 | A1 |
