# Supplementary material for: Capturing the Biofuel Wellhead and Powerhouse: The Chloroplast and Mitochondrial Genomes of the Leguminous Feedstock Tree Pongamia pinnata
Source: PLoS One. 2012 Dec 14;7(12):e51687. doi: 10.1371/journal.pone.0051687 (PMC3522722; doi:10.1371/journal.pone.0051687)
Supplement: Table S4 — Table of potential RNA editing sites in a list of thirty-three unique Pongamia mitochondrial genes. A description of the column headings can be found in the Table S3 legend. (DOCX) [file pone.0051687.s014.docx]

**Table S4**

| **Gene** | **Pos** | **X** | **Y** | **ABR** | **A** | **C** | **G** | **T** | **N** | **Del** | **Ins** |
| --- | --- | --- | --- | --- | --- | --- | --- | --- | --- | --- | --- |
| ***nad2*** | 26 | C | T | 1.000 | 0 | 0 | 0 | 4 | 0 | 0 | 0 |
| ***nad2*** | 56 | C | T | 0.800 | 0 | 2 | 0 | 8 | 0 | 0 | 0 |
| ***nad2*** | 303 | C | T | 0.667 | 0 | 6 | 0 | 12 | 0 | 0 | 0 |
| ***nad2*** | 308 | C | T | 0.765 | 0 | 4 | 0 | 13 | 0 | 1 | 0 |
| ***nad2*** | 311 | C | T | 0.947 | 0 | 1 | 0 | 18 | 0 | 0 | 0 |
| ***nad2*** | 356 | C | T | 1.000 | 0 | 0 | 0 | 16 | 0 | 0 | 0 |
| ***nad2*** | 361 | C | T | 1.000 | 0 | 0 | 0 | 13 | 0 | 0 | 0 |
| ***nad2*** | 367 | C | T | 1.000 | 0 | 0 | 0 | 9 | 0 | 0 | 0 |
| ***nad2*** | 394 | C | T | 0.875 | 0 | 1 | 0 | 7 | 0 | 0 | 0 |
| ***nad2*** | 401 | C | T | 0.875 | 0 | 1 | 0 | 7 | 0 | 0 | 0 |
| ***nad2*** | 428 | C | T | 1.000 | 0 | 0 | 0 | 20 | 0 | 0 | 0 |
| ***nad2*** | 497 | C | T | 0.938 | 0 | 1 | 0 | 15 | 0 | 0 | 0 |
| ***nad2*** | 609 | C | T | 0.696 | 0 | 7 | 0 | 16 | 0 | 0 | 0 |
| ***nad2*** | 662 | C | T | 0.773 | 0 | 5 | 0 | 17 | 0 | 0 | 0 |
| ***nad2*** | 677 | C | T | 1.000 | 0 | 0 | 0 | 21 | 0 | 0 | 0 |
| ***nad2*** | 800 | C | T | 0.783 | 0 | 5 | 0 | 18 | 0 | 0 | 0 |
| ***nad2*** | 809 | C | T | 0.696 | 0 | 7 | 0 | 16 | 0 | 0 | 0 |
| ***nad2*** | 920 | C | T | 0.889 | 0 | 3 | 0 | 24 | 0 | 0 | 0 |
| ***nad2*** | 928 | C | T | 0.893 | 0 | 3 | 0 | 25 | 0 | 0 | 0 |
| ***nad2*** | 958 | C | T | 0.952 | 0 | 1 | 0 | 20 | 0 | 0 | 0 |
| ***nad2*** | 962 | C | T | 0.933 | 0 | 1 | 0 | 14 | 0 | 0 | 0 |
| ***nad2*** | 1127 | C | T | 0.927 | 0 | 3 | 0 | 38 | 0 | 0 | 0 |
| ***nad2*** | 1246 | C | T | 0.909 | 0 | 1 | 0 | 10 | 0 | 0 | 0 |
| ***nad2*** | 1298 | C | T | 0.933 | 0 | 2 | 0 | 28 | 0 | 0 | 0 |
| ***nad2*** | 1400 | C | T | 0.850 | 0 | 3 | 0 | 17 | 0 | 0 | 0 |
| ***nad2*** | 1403 | C | T | 0.850 | 0 | 3 | 0 | 17 | 0 | 0 | 0 |
| ***nad2*** | 1408 | C | T | 0.842 | 0 | 3 | 0 | 16 | 0 | 0 | 0 |
| ***nad2*** | 1409 | C | T | 0.842 | 0 | 3 | 0 | 16 | 0 | 0 | 0 |
| ***nad2*** | 1416 | C | T | 0.684 | 0 | 6 | 0 | 13 | 0 | 0 | 0 |
| ***nad2*** | 1457 | C | T | 0.941 | 0 | 2 | 0 | 32 | 0 | 0 | 0 |
| ***cox1*** | 11 | C | T | 1.000 | 0 | 0 | 0 | 23 | 0 | 0 | 0 |
| ***cox1*** | 15 | C | T | 0.500 | 0 | 12 | 0 | 12 | 0 | 0 | 0 |
| ***cox1*** | 242 | C | T | 0.900 | 0 | 1 | 0 | 9 | 0 | 0 | 0 |
| ***cox1*** | 254 | C | T | 1.000 | 0 | 0 | 0 | 14 | 0 | 0 | 0 |
| ***cox1*** | 443 | C | T | 1.000 | 0 | 0 | 0 | 22 | 0 | 0 | 0 |
| ***cox1*** | 452 | C | T | 1.000 | 0 | 0 | 0 | 21 | 0 | 0 | 0 |
| ***cox1*** | 515 | C | T | 0.985 | 0 | 1 | 0 | 65 | 0 | 0 | 0 |
| ***cox1*** | 551 | C | T | 0.928 | 0 | 6 | 0 | 77 | 0 | 0 | 0 |
| ***cox1*** | 575 | C | T | 1.000 | 0 | 0 | 0 | 31 | 0 | 0 | 0 |
| ***cox1*** | 590 | C | T | 1.000 | 0 | 0 | 0 | 24 | 0 | 0 | 0 |
| ***cox1*** | 668 | C | T | 0.950 | 0 | 3 | 0 | 57 | 0 | 0 | 0 |
| ***cox1*** | 715 | C | T | 0.833 | 0 | 1 | 0 | 5 | 0 | 0 | 0 |
| ***cox1*** | 746 | C | T | 0.977 | 0 | 1 | 0 | 42 | 0 | 0 | 0 |
| ***cox1*** | 761 | C | T | 0.963 | 0 | 2 | 0 | 52 | 0 | 0 | 0 |
| ***cox1*** | 1079 | C | T | 1.000 | 0 | 0 | 0 | 9 | 0 | 0 | 0 |
| ***cox1*** | 1186 | C | T | 0.909 | 0 | 1 | 0 | 10 | 0 | 0 | 0 |
| ***cox1*** | 1279 | C | T | 0.957 | 0 | 2 | 0 | 44 | 0 | 0 | 0 |
| ***cox1*** | 1402 | C | T | 0.974 | 0 | 2 | 0 | 74 | 0 | 0 | 0 |
| ***cox1*** | 1405 | C | T | 0.989 | 0 | 1 | 0 | 86 | 0 | 0 | 0 |
| ***cox1*** | 1433 | C | T | 0.985 | 0 | 2 | 0 | 132 | 0 | 0 | 0 |
| ***ccmB*** | 43 | C | T | 0.762 | 0 | 5 | 0 | 16 | 0 | 0 | 0 |
| ***ccmB*** | 71 | C | T | 0.957 | 0 | 1 | 0 | 22 | 0 | 0 | 0 |
| ***ccmB*** | 128 | C | T | 0.786 | 0 | 3 | 0 | 11 | 0 | 1 | 0 |
| ***ccmB*** | 137 | C | T | 0.846 | 0 | 2 | 0 | 11 | 0 | 0 | 0 |
| ***ccmB*** | 154 | C | T | 0.600 | 0 | 6 | 0 | 9 | 0 | 0 | 0 |
| ***ccmB*** | 160 | C | T | 0.400 | 0 | 9 | 0 | 6 | 0 | 0 | 0 |
| ***ccmB*** | 164 | C | T | 0.400 | 0 | 9 | 0 | 6 | 0 | 0 | 0 |
| ***ccmB*** | 179 | C | T | 0.688 | 0 | 5 | 0 | 11 | 0 | 0 | 0 |
| ***ccmB*** | 181 | C | T | 0.500 | 0 | 8 | 0 | 8 | 0 | 0 | 0 |
| ***ccmB*** | 286 | C | T | 0.727 | 0 | 3 | 0 | 8 | 0 | 0 | 0 |
| ***ccmB*** | 338 | C | T | 0.897 | 0 | 4 | 0 | 35 | 0 | 0 | 0 |
| ***ccmB*** | 367 | C | T | 0.808 | 0 | 5 | 0 | 21 | 0 | 0 | 0 |
| ***ccmB*** | 379 | C | T | 0.588 | 0 | 7 | 0 | 10 | 0 | 0 | 0 |
| ***ccmB*** | 380 | C | T | 0.667 | 0 | 5 | 0 | 10 | 0 | 0 | 0 |
| ***ccmB*** | 424 | C | T | 0.750 | 0 | 1 | 0 | 3 | 0 | 0 | 0 |
| ***ccmB*** | 467 | C | T | 0.722 | 0 | 10 | 0 | 26 | 0 | 0 | 0 |
| ***ccmB*** | 475 | C | T | 0.778 | 0 | 8 | 0 | 28 | 0 | 0 | 0 |
| ***ccmB*** | 476 | C | T | 0.714 | 0 | 10 | 0 | 25 | 0 | 0 | 0 |
| ***ccmB*** | 485 | C | T | 0.657 | 0 | 12 | 0 | 23 | 0 | 0 | 0 |
| ***ccmB*** | 494 | C | T | 0.686 | 0 | 11 | 0 | 24 | 0 | 0 | 0 |
| ***nad1*** | 215 | C | T | 0.889 | 0 | 1 | 0 | 8 | 0 | 0 | 0 |
| ***nad1*** | 265 | C | T | 0.917 | 0 | 1 | 0 | 11 | 0 | 0 | 0 |
| ***nad1*** | 308 | C | T | 0.923 | 0 | 1 | 0 | 12 | 0 | 0 | 0 |
| ***nad1*** | 368 | C | T | 0.900 | 0 | 1 | 0 | 9 | 0 | 0 | 0 |
| ***nad1*** | 490 | C | T | 1.000 | 0 | 0 | 0 | 3 | 0 | 0 | 0 |
| ***nad1*** | 493 | C | T | 1.000 | 0 | 0 | 0 | 4 | 0 | 0 | 0 |
| ***nad1*** | 500 | C | T | 0.600 | 0 | 2 | 0 | 3 | 0 | 0 | 0 |
| ***nad1*** | 536 | C | T | 1.000 | 0 | 0 | 0 | 7 | 0 | 0 | 0 |
| ***nad1*** | 573 | C | T | 0.941 | 0 | 1 | 0 | 16 | 0 | 0 | 0 |
| ***nad1*** | 580 | C | T | 0.833 | 0 | 3 | 0 | 15 | 0 | 0 | 0 |
| ***nad1*** | 635 | C | T | 0.957 | 0 | 1 | 0 | 22 | 0 | 0 | 0 |
| ***nad1*** | 674 | C | T | 1.000 | 0 | 0 | 0 | 19 | 0 | 0 | 0 |
| ***nad1*** | 725 | C | T | 0.952 | 0 | 1 | 0 | 20 | 0 | 0 | 0 |
| ***nad1*** | 734 | C | T | 0.941 | 0 | 1 | 0 | 16 | 0 | 0 | 0 |
| ***nad1*** | 740 | C | T | 0.938 | 0 | 1 | 0 | 15 | 0 | 0 | 0 |
| ***nad1*** | 743 | C | T | 0.933 | 0 | 1 | 0 | 14 | 0 | 0 | 0 |
| ***nad1*** | 755 | C | T | 0.909 | 0 | 1 | 0 | 10 | 0 | 0 | 0 |
| ***nad1*** | 898 | C | T | 0.857 | 0 | 1 | 0 | 6 | 0 | 0 | 0 |
| ***nad1*** | 928 | C | T | 0.750 | 0 | 2 | 0 | 6 | 0 | 0 | 0 |
| ***nad1*** | 953 | C | T | 1.000 | 0 | 0 | 0 | 7 | 0 | 0 | 0 |
| ***atp1*** | 246 | C | T | 0.663 | 1 | 141 | 0 | 277 | 0 | 0 | 0 |
| ***atp1*** | 1484 | C | T | 0.972 | 0 | 15 | 1 | 528 | 0 | 0 | 0 |
| ***sdh3*** | 352 | T | C | 0.559 | 0 | 19 | 0 | 15 | 0 | 0 | 0 |
| ***sdh3*** | 355 | A | C | 0.500 | 17 | 17 | 1 | 0 | 0 | 0 | 0 |
| ***sdh3*** | 357 | T | C | 0.429 | 0 | 15 | 0 | 20 | 0 | 0 | 0 |
| ***sdh3*** | 713 | C | T | 0.500 | 0 | 3 | 0 | 3 | 0 | 0 | 0 |
| ***ccmFn*** | 145 | C | T | 1.000 | 0 | 0 | 0 | 4 | 0 | 0 | 0 |
| ***ccmFn*** | 154 | C | T | 1.000 | 0 | 0 | 0 | 4 | 0 | 0 | 0 |
| ***ccmFn*** | 251 | C | T | 1.000 | 0 | 0 | 0 | 4 | 0 | 0 | 0 |
| ***ccmFn*** | 259 | C | T | 1.000 | 0 | 0 | 0 | 4 | 0 | 0 | 0 |
| ***ccmFn*** | 266 | C | T | 0.800 | 0 | 1 | 0 | 4 | 0 | 0 | 0 |
| ***ccmFn*** | 268 | C | T | 1.000 | 0 | 0 | 0 | 5 | 0 | 0 | 0 |
| ***ccmFn*** | 282 | C | T | 0.800 | 0 | 1 | 0 | 4 | 0 | 0 | 0 |
| ***ccmFn*** | 286 | C | T | 1.000 | 0 | 0 | 0 | 5 | 0 | 0 | 0 |
| ***ccmFn*** | 365 | C | T | 0.714 | 0 | 2 | 0 | 5 | 0 | 0 | 0 |
| ***ccmFn*** | 372 | C | T | 1.000 | 0 | 0 | 0 | 9 | 0 | 0 | 0 |
| ***ccmFn*** | 1572 | A | T | 1.000 | 0 | 0 | 0 | 3 | 0 | 0 | 0 |
| ***nad4L*** | 8 | C | T | 0.600 | 0 | 2 | 0 | 3 | 0 | 0 | 0 |
| ***nad4L*** | 41 | C | T | 0.875 | 0 | 1 | 0 | 7 | 0 | 0 | 0 |
| ***nad4L*** | 55 | C | T | 0.800 | 0 | 2 | 0 | 8 | 0 | 0 | 0 |
| ***nad4L*** | 86 | C | T | 0.800 | 1 | 3 | 0 | 12 | 0 | 0 | 0 |
| ***nad4L*** | 95 | C | T | 0.882 | 0 | 2 | 0 | 15 | 0 | 0 | 0 |
| ***nad4L*** | 100 | C | T | 0.737 | 0 | 5 | 0 | 14 | 0 | 0 | 0 |
| ***nad4L*** | 110 | C | T | 0.913 | 0 | 2 | 0 | 21 | 0 | 0 | 0 |
| ***nad4L*** | 131 | C | T | 0.783 | 0 | 5 | 0 | 18 | 0 | 0 | 0 |
| ***nad4L*** | 158 | C | T | 0.933 | 0 | 1 | 0 | 14 | 0 | 0 | 0 |
| ***nad4L*** | 179 | C | T | 0.833 | 0 | 2 | 0 | 10 | 0 | 0 | 0 |
| ***nad4L*** | 188 | C | T | 0.842 | 0 | 3 | 0 | 16 | 0 | 0 | 0 |
| ***nad4L*** | 197 | C | T | 0.647 | 0 | 6 | 0 | 11 | 0 | 0 | 0 |
| ***atp4*** | 56 | C | T | 0.976 | 0 | 2 | 0 | 81 | 0 | 0 | 0 |
| ***atp4*** | 59 | C | T | 0.952 | 0 | 4 | 0 | 80 | 0 | 0 | 0 |
| ***atp4*** | 89 | C | T | 0.921 | 0 | 5 | 0 | 58 | 0 | 0 | 0 |
| ***atp4*** | 118 | C | T | 0.992 | 0 | 1 | 0 | 119 | 0 | 0 | 0 |
| ***atp4*** | 215 | C | T | 0.935 | 0 | 7 | 0 | 100 | 0 | 0 | 0 |
| ***atp4*** | 227 | C | T | 0.940 | 0 | 9 | 0 | 142 | 0 | 0 | 0 |
| ***atp4*** | 248 | C | T | 0.816 | 0 | 37 | 0 | 164 | 0 | 0 | 0 |
| ***atp4*** | 251 | C | T | 0.911 | 0 | 18 | 0 | 185 | 0 | 0 | 0 |
| ***atp4*** | 395 | C | T | 0.893 | 1 | 13 | 0 | 108 | 0 | 0 | 0 |
| ***atp4*** | 407 | C | T | 0.927 | 0 | 8 | 0 | 102 | 0 | 0 | 0 |
| ***atp4*** | 416 | C | T | 0.903 | 0 | 22 | 1 | 205 | 0 | 0 | 0 |
| ***rps10*** | 16 | C | T | 1.000 | 0 | 0 | 0 | 6 | 0 | 0 | 0 |
| ***rps10*** | 238 | C | T | 0.974 | 0 | 4 | 0 | 151 | 0 | 0 | 0 |
| ***rps10*** | 302 | C | T | 1.000 | 0 | 0 | 0 | 36 | 0 | 0 | 0 |
| ***rps10*** | 331 | C | T | 1.000 | 0 | 0 | 0 | 82 | 0 | 0 | 0 |
| ***cox2*** | 8 | A | T | 1.000 | 0 | 0 | 0 | 3 | 0 | 0 | 0 |
| ***cox2*** | 9 | C | A | 1.000 | 22 | 0 | 0 | 0 | 0 | 0 | 0 |
| ***cox2*** | 26 | C | T | 1.000 | 0 | 0 | 0 | 121 | 0 | 0 | 0 |
| ***cox2*** | 27 | G | T | 1.000 | 0 | 0 | 0 | 121 | 0 | 0 | 0 |
| ***cox2*** | 28 | A | C | 1.000 | 0 | 121 | 0 | 0 | 0 | 0 | 0 |
| ***cox2*** | 160 | C | T | 0.955 | 0 | 10 | 0 | 213 | 0 | 0 | 0 |
| ***cox2*** | 250 | C | T | 0.964 | 0 | 2 | 0 | 53 | 0 | 0 | 0 |
| ***cox2*** | 275 | C | T | 0.952 | 1 | 7 | 0 | 139 | 0 | 0 | 0 |
| ***cox2*** | 376 | C | T | 1.000 | 0 | 0 | 0 | 115 | 0 | 0 | 0 |
| ***cox2*** | 440 | C | T | 0.978 | 0 | 4 | 1 | 176 | 0 | 0 | 0 |
| ***cox2*** | 473 | C | T | 0.973 | 0 | 4 | 0 | 145 | 0 | 0 | 0 |
| ***cox2*** | 541 | C | T | 1.000 | 0 | 0 | 0 | 43 | 0 | 0 | 0 |
| ***cox2*** | 554 | C | T | 1.000 | 0 | 0 | 0 | 43 | 0 | 0 | 0 |
| ***cox2*** | 629 | C | T | 0.953 | 0 | 10 | 0 | 205 | 0 | 0 | 0 |
| ***cox2*** | 695 | C | T | 0.986 | 0 | 1 | 0 | 72 | 0 | 0 | 0 |
| ***cox2*** | 718 | C | T | 0.910 | 1 | 13 | 0 | 132 | 0 | 0 | 0 |
| ***cox2*** | 739 | C | T | 0.947 | 0 | 8 | 1 | 142 | 0 | 0 | 0 |
| ***cox3*** | 245 | C | T | 0.889 | 0 | 3 | 0 | 24 | 0 | 0 | 0 |
| ***cox3*** | 257 | C | T | 0.875 | 0 | 3 | 0 | 21 | 0 | 0 | 0 |
| ***cox3*** | 289 | C | T | 0.920 | 0 | 4 | 0 | 46 | 0 | 0 | 0 |
| ***cox3*** | 304 | C | T | 0.923 | 0 | 4 | 0 | 48 | 0 | 0 | 0 |
| ***cox3*** | 311 | C | T | 0.891 | 0 | 6 | 0 | 49 | 0 | 0 | 0 |
| ***cox3*** | 314 | C | T | 0.907 | 0 | 5 | 0 | 49 | 0 | 0 | 0 |
| ***cox3*** | 388 | C | T | 0.962 | 0 | 2 | 0 | 51 | 0 | 0 | 0 |
| ***cox3*** | 413 | C | T | 0.918 | 0 | 5 | 0 | 56 | 0 | 0 | 0 |
| ***cox3*** | 512 | C | T | 0.907 | 0 | 5 | 0 | 49 | 0 | 0 | 0 |
| ***atp8*** | 30 | C | T | 0.558 | 0 | 38 | 0 | 48 | 0 | 0 | 0 |
| ***atp8*** | 47 | C | T | 0.861 | 0 | 10 | 0 | 62 | 0 | 0 | 0 |
| ***atp8*** | 58 | C | T | 0.882 | 0 | 4 | 0 | 30 | 0 | 0 | 0 |
| ***atp8*** | 76 | C | T | 0.459 | 0 | 20 | 0 | 17 | 0 | 0 | 0 |
| ***atp8*** | 77 | C | T | 0.784 | 0 | 8 | 0 | 29 | 0 | 0 | 0 |
| ***atp8*** | 93 | A | G | 1.000 | 0 | 0 | 73 | 0 | 0 | 0 | 0 |
| ***atp8*** | 455 | C | T | 0.989 | 1 | 19 | 0 | 1782 | 0 | 0 | 0 |
| ***nad5*** | 242 | C | T | 0.800 | 0 | 1 | 0 | 4 | 0 | 0 | 0 |
| ***nad5*** | 272 | C | T | 1.000 | 0 | 0 | 0 | 4 | 0 | 0 | 0 |
| ***nad5*** | 358 | C | T | 0.857 | 0 | 1 | 0 | 6 | 0 | 0 | 0 |
| ***nad5*** | 374 | C | T | 0.750 | 0 | 1 | 0 | 3 | 0 | 0 | 0 |
| ***nad5*** | 398 | C | T | 0.667 | 0 | 2 | 0 | 4 | 0 | 0 | 0 |
| ***nad5*** | 494 | C | T | 0.789 | 0 | 4 | 0 | 15 | 0 | 0 | 0 |
| ***nad5*** | 506 | C | T | 0.750 | 0 | 3 | 0 | 9 | 0 | 0 | 0 |
| ***nad5*** | 539 | C | T | 0.667 | 0 | 4 | 0 | 8 | 0 | 0 | 0 |
| ***nad5*** | 548 | C | T | 0.688 | 0 | 5 | 0 | 11 | 0 | 0 | 0 |
| ***nad5*** | 553 | C | T | 0.611 | 0 | 7 | 0 | 11 | 0 | 0 | 0 |
| ***nad5*** | 598 | C | T | 0.792 | 0 | 5 | 0 | 19 | 0 | 0 | 0 |
| ***nad5*** | 608 | C | T | 0.609 | 0 | 9 | 0 | 14 | 0 | 0 | 0 |
| ***nad5*** | 629 | C | T | 0.857 | 0 | 2 | 0 | 12 | 0 | 0 | 0 |
| ***nad5*** | 676 | C | T | 0.714 | 0 | 2 | 0 | 5 | 0 | 0 | 0 |
| ***nad5*** | 713 | C | T | 0.667 | 0 | 3 | 0 | 6 | 0 | 0 | 0 |
| ***nad5*** | 725 | C | T | 0.786 | 0 | 3 | 0 | 11 | 0 | 0 | 0 |
| ***nad5*** | 835 | C | T | 0.867 | 0 | 2 | 0 | 13 | 0 | 0 | 0 |
| ***nad5*** | 863 | C | T | 0.786 | 0 | 3 | 0 | 11 | 0 | 0 | 0 |
| ***nad5*** | 875 | C | T | 0.800 | 0 | 2 | 0 | 8 | 0 | 0 | 0 |
| ***nad5*** | 1310 | C | T | 0.462 | 0 | 7 | 0 | 6 | 0 | 0 | 0 |
| ***nad5*** | 1413 | T | G | 1.000 | 0 | 0 | 21 | 0 | 0 | 0 | 0 |
| ***nad5*** | 1490 | C | T | 1.000 | 0 | 0 | 0 | 56 | 0 | 0 | 0 |
| ***nad5*** | 1550 | C | T | 0.833 | 0 | 1 | 0 | 5 | 0 | 0 | 0 |
| ***nad5*** | 1580 | C | T | 1.000 | 0 | 0 | 0 | 11 | 0 | 0 | 0 |
| ***nad5*** | 1589 | C | T | 0.870 | 0 | 3 | 0 | 20 | 0 | 0 | 0 |
| ***nad5*** | 1610 | C | T | 0.919 | 0 | 3 | 0 | 34 | 0 | 0 | 0 |
| ***nad5*** | 1895 | C | T | 1.000 | 0 | 0 | 0 | 17 | 0 | 0 | 0 |
| ***nad5*** | 1916 | C | T | 0.917 | 0 | 1 | 0 | 11 | 0 | 0 | 0 |
| ***nad5*** | 1918 | C | T | 0.917 | 0 | 1 | 0 | 11 | 0 | 0 | 0 |
| ***nad6*** | 146 | C | T | 1.000 | 0 | 0 | 0 | 8 | 0 | 0 | 0 |
| ***nad6*** | 161 | C | T | 0.900 | 1 | 1 | 0 | 9 | 0 | 0 | 0 |
| ***nad6*** | 169 | C | T | 1.000 | 0 | 0 | 0 | 11 | 0 | 0 | 0 |
| ***nad6*** | 191 | C | T | 0.909 | 0 | 1 | 0 | 10 | 0 | 0 | 0 |
| ***nad6*** | 289 | C | T | 1.000 | 0 | 0 | 0 | 10 | 0 | 0 | 0 |
| ***nad6*** | 463 | C | T | 1.000 | 0 | 0 | 0 | 27 | 0 | 0 | 0 |
| ***rps4m*** | 22 | C | T | 0.833 | 0 | 1 | 0 | 5 | 0 | 0 | 0 |
| ***rps4m*** | 65 | C | T | 0.923 | 0 | 1 | 0 | 12 | 0 | 0 | 0 |
| ***rps4m*** | 76 | C | T | 0.833 | 0 | 2 | 0 | 10 | 0 | 0 | 0 |
| ***rps4m*** | 160 | C | T | 0.750 | 0 | 3 | 0 | 9 | 0 | 0 | 0 |
| ***rps4m*** | 191 | C | T | 0.667 | 0 | 4 | 0 | 8 | 0 | 0 | 0 |
| ***rps4m*** | 211 | C | T | 0.600 | 0 | 4 | 0 | 6 | 0 | 0 | 0 |
| ***rps4m*** | 220 | C | T | 0.462 | 0 | 7 | 0 | 6 | 0 | 0 | 0 |
| ***rps4m*** | 293 | C | T | 0.727 | 0 | 3 | 0 | 8 | 0 | 0 | 0 |
| ***rps4m*** | 305 | C | T | 0.727 | 0 | 3 | 0 | 8 | 0 | 0 | 0 |
| ***rps4m*** | 317 | C | T | 0.818 | 0 | 2 | 0 | 9 | 0 | 0 | 0 |
| ***rps4m*** | 334 | C | T | 0.750 | 0 | 2 | 0 | 6 | 0 | 0 | 0 |
| ***rps4m*** | 491 | C | T | 0.889 | 0 | 1 | 0 | 8 | 0 | 0 | 0 |
| ***rps4m*** | 509 | C | T | 0.600 | 0 | 2 | 0 | 3 | 0 | 0 | 0 |
| ***rps4m*** | 870 | C | T | 0.946 | 0 | 2 | 0 | 35 | 0 | 0 | 0 |
| ***rps4m*** | 944 | C | T | 1.000 | 0 | 0 | 0 | 5 | 0 | 0 | 0 |
| ***rps4m*** | 955 | C | T | 1.000 | 0 | 0 | 0 | 4 | 0 | 0 | 0 |
| ***rps4m*** | 965 | C | T | 0.500 | 0 | 3 | 0 | 3 | 0 | 0 | 0 |
| ***nad9*** | 14 | C | T | 1.000 | 0 | 0 | 0 | 4 | 0 | 0 | 0 |
| ***nad9*** | 92 | C | T | 0.914 | 0 | 3 | 0 | 32 | 0 | 0 | 0 |
| ***nad9*** | 167 | C | T | 0.692 | 0 | 4 | 0 | 9 | 0 | 0 | 0 |
| ***nad9*** | 298 | C | T | 0.750 | 0 | 2 | 0 | 6 | 0 | 0 | 0 |
| ***nad9*** | 328 | C | T | 0.941 | 0 | 1 | 0 | 16 | 0 | 0 | 0 |
| ***nad9*** | 368 | C | T | 0.955 | 0 | 1 | 0 | 21 | 0 | 0 | 0 |
| ***nad9*** | 398 | C | T | 1.000 | 0 | 0 | 0 | 16 | 0 | 0 | 0 |
| ***nad9*** | 439 | C | T | 1.000 | 0 | 0 | 0 | 25 | 0 | 0 | 0 |
| ***nad9*** | 480 | G | A | 1.000 | 13 | 0 | 0 | 0 | 0 | 0 | 0 |
| ***mttB*** | 164 | C | T | 0.429 | 0 | 4 | 0 | 3 | 0 | 0 | 0 |
| ***mttB*** | 238 | C | T | 0.667 | 0 | 2 | 0 | 4 | 0 | 0 | 0 |
| ***mttB*** | 329 | C | T | 1.000 | 0 | 0 | 0 | 6 | 0 | 0 | 0 |
| ***mttB*** | 349 | C | T | 1.000 | 0 | 0 | 0 | 8 | 0 | 0 | 0 |
| ***mttB*** | 352 | C | T | 1.000 | 0 | 0 | 0 | 8 | 0 | 0 | 0 |
| ***mttB*** | 355 | C | T | 1.000 | 0 | 0 | 0 | 8 | 0 | 0 | 0 |
| ***mttB*** | 448 | C | T | 1.000 | 0 | 0 | 0 | 4 | 0 | 0 | 0 |
| ***mttB*** | 473 | C | T | 0.857 | 0 | 1 | 0 | 6 | 0 | 0 | 0 |
| ***mttB*** | 481 | C | T | 1.000 | 0 | 0 | 0 | 7 | 0 | 0 | 0 |
| ***mttB*** | 524 | C | T | 1.000 | 0 | 0 | 0 | 4 | 0 | 0 | 0 |
| ***mttB*** | 530 | C | T | 0.833 | 0 | 1 | 0 | 5 | 0 | 0 | 0 |
| ***mttB*** | 586 | C | T | 0.783 | 0 | 5 | 0 | 18 | 0 | 0 | 0 |
| ***mttB*** | 592 | C | T | 0.478 | 0 | 12 | 0 | 11 | 0 | 0 | 0 |
| ***mttB*** | 636 | C | T | 0.943 | 0 | 2 | 0 | 33 | 0 | 0 | 0 |
| ***mttB*** | 643 | C | T | 0.903 | 0 | 3 | 0 | 28 | 0 | 0 | 0 |
| ***mttB*** | 659 | C | T | 0.611 | 0 | 7 | 0 | 11 | 0 | 0 | 0 |
| ***atp9*** | 20 | C | T | 1.000 | 0 | 0 | 0 | 132 | 0 | 0 | 0 |
| ***atp9*** | 50 | C | T | 0.994 | 2 | 12 | 4 | 2075 | 0 | 2 | 0 |
| ***atp9*** | 202 | T | A | 0.500 | 5 | 0 | 2 | 5 | 0 | 0 | 0 |
| ***ccmC*** | 76 | C | T | 1.000 | 0 | 0 | 0 | 5 | 0 | 0 | 0 |
| ***ccmC*** | 103 | C | T | 1.000 | 0 | 0 | 0 | 4 | 0 | 0 | 0 |
| ***ccmC*** | 115 | C | T | 1.000 | 0 | 0 | 0 | 5 | 0 | 0 | 0 |
| ***ccmC*** | 179 | C | T | 1.000 | 0 | 0 | 0 | 3 | 0 | 0 | 0 |
| ***ccmC*** | 548 | C | T | 1.000 | 0 | 0 | 0 | 7 | 0 | 0 | 0 |
| ***ccmC*** | 568 | C | T | 0.800 | 0 | 2 | 0 | 8 | 0 | 0 | 0 |
| ***ccmC*** | 575 | C | T | 0.800 | 0 | 2 | 0 | 8 | 0 | 0 | 0 |
| ***ccmC*** | 608 | C | T | 0.500 | 0 | 3 | 0 | 3 | 0 | 0 | 0 |
| ***ccmC*** | 673 | C | T | 1.000 | 0 | 0 | 0 | 7 | 0 | 0 | 0 |
| ***rps3m*** | 64 | C | T | 0.944 | 0 | 1 | 0 | 17 | 0 | 0 | 0 |
| ***rps3m*** | 69 | C | T | 0.760 | 0 | 6 | 0 | 19 | 0 | 0 | 0 |
| ***rps3m*** | 512 | C | T | 0.926 | 0 | 4 | 0 | 50 | 0 | 0 | 0 |
| ***rps3m*** | 713 | C | T | 1.000 | 0 | 0 | 0 | 21 | 0 | 0 | 0 |
| ***rps3m*** | 890 | C | T | 0.960 | 0 | 1 | 0 | 24 | 0 | 0 | 0 |
| ***rps3m*** | 1031 | C | T | 0.889 | 0 | 1 | 0 | 8 | 0 | 0 | 0 |
| ***rps3m*** | 1345 | C | A | 1.000 | 147 | 0 | 0 | 0 | 0 | 0 | 0 |
| ***rps3m*** | 1370 | C | T | 1.000 | 0 | 0 | 0 | 93 | 0 | 0 | 0 |
| ***rps3m*** | 1508 | C | T | 0.960 | 0 | 58 | 1 | 1375 | 0 | 1 | 0 |
| ***rps3m*** | 1561 | C | T | 0.900 | 0 | 14 | 0 | 126 | 0 | 0 | 0 |
| ***rps3m*** | 1625 | C | T | 0.981 | 1 | 26 | 2 | 1322 | 0 | 0 | 0 |
| ***rpl16m*** | 15 | C | T | 0.899 | 0 | 29 | 0 | 259 | 0 | 0 | 0 |
| ***rpl16m*** | 79 | C | T | 0.986 | 0 | 3 | 0 | 206 | 0 | 0 | 0 |
| ***rpl16m*** | 227 | C | T | 0.978 | 0 | 11 | 0 | 478 | 0 | 0 | 0 |
| ***rpl16m*** | 524 | C | T | 0.963 | 1 | 12 | 0 | 314 | 0 | 0 | T1 |
| ***rpl16m*** | 530 | C | T | 0.984 | 0 | 5 | 0 | 315 | 0 | 0 | 0 |
| ***ccmFc*** | 50 | C | T | 1.000 | 0 | 0 | 0 | 7 | 0 | 0 | 0 |
| ***ccmFc*** | 52 | C | T | 0.714 | 0 | 2 | 0 | 5 | 0 | 0 | 0 |
| ***ccmFc*** | 103 | C | T | 0.818 | 0 | 2 | 0 | 9 | 0 | 0 | 0 |
| ***ccmFc*** | 122 | C | T | 0.600 | 0 | 2 | 1 | 3 | 0 | 0 | 0 |
| ***ccmFc*** | 305 | C | T | 1.000 | 0 | 0 | 0 | 15 | 0 | 0 | 0 |
| ***ccmFc*** | 310 | C | T | 0.933 | 0 | 1 | 0 | 14 | 0 | 0 | 0 |
| ***ccmFc*** | 315 | C | T | 0.571 | 0 | 6 | 0 | 8 | 0 | 0 | 0 |
| ***ccmFc*** | 334 | C | T | 0.923 | 0 | 1 | 0 | 12 | 0 | 0 | 0 |
| ***ccmFc*** | 406 | C | T | 0.826 | 0 | 4 | 0 | 19 | 0 | 0 | 0 |
| ***ccmFc*** | 896 | C | T | 0.933 | 0 | 1 | 0 | 14 | 0 | 0 | 0 |
| ***ccmFc*** | 1095 | C | T | 0.610 | 0 | 16 | 0 | 25 | 0 | 0 | 0 |
| ***ccmFc*** | 1157 | C | T | 1.000 | 0 | 0 | 0 | 20 | 0 | 0 | 0 |
| ***ccmFc*** | 1178 | C | T | 0.857 | 0 | 4 | 0 | 24 | 0 | 0 | 0 |
| ***ccmFc*** | 1252 | C | T | 0.429 | 0 | 4 | 0 | 3 | 0 | 0 | 0 |
| ***ccmFc*** | 1277 | C | T | 0.400 | 0 | 6 | 0 | 4 | 0 | 0 | 0 |
| ***atp6*** | 37 | C | T | 0.933 | 0 | 1 | 0 | 14 | 0 | 0 | 0 |
| ***atp6*** | 116 | C | T | 0.467 | 0 | 8 | 0 | 7 | 0 | 0 | 0 |
| ***atp6*** | 167 | C | T | 0.667 | 0 | 6 | 0 | 12 | 0 | 0 | 0 |
| ***atp6*** | 227 | C | T | 0.429 | 0 | 4 | 0 | 3 | 0 | 0 | 0 |
| ***atp6*** | 245 | C | T | 0.778 | 0 | 4 | 0 | 14 | 0 | 0 | 0 |
| ***atp6*** | 260 | C | T | 0.824 | 0 | 3 | 0 | 14 | 0 | 0 | 0 |
| ***atp6*** | 261 | C | T | 0.588 | 0 | 7 | 0 | 10 | 0 | 0 | 0 |
| ***atp6*** | 392 | C | T | 0.893 | 0 | 6 | 0 | 50 | 0 | 0 | 0 |
| ***atp6*** | 451 | C | T | 0.917 | 0 | 4 | 0 | 44 | 0 | 0 | 0 |
| ***atp6*** | 454 | C | T | 0.936 | 0 | 3 | 0 | 44 | 0 | 1 | 0 |
| ***atp6*** | 476 | C | T | 0.885 | 0 | 3 | 0 | 23 | 0 | 0 | 0 |
| ***nad3*** | 61 | C | T | 1.000 | 0 | 0 | 0 | 9 | 0 | 0 | 0 |
| ***nad3*** | 62 | C | T | 1.000 | 0 | 0 | 0 | 9 | 0 | 0 | 0 |
| ***nad3*** | 80 | C | T | 0.846 | 0 | 2 | 0 | 11 | 0 | 0 | 0 |
| ***nad3*** | 208 | C | T | 0.789 | 0 | 4 | 0 | 15 | 0 | 0 | 0 |
| ***nad3*** | 209 | C | T | 0.789 | 0 | 4 | 0 | 15 | 0 | 0 | 0 |
| ***nad3*** | 215 | C | T | 0.706 | 0 | 5 | 0 | 12 | 0 | 0 | 0 |
| ***nad3*** | 230 | C | T | 0.727 | 0 | 3 | 0 | 8 | 0 | 0 | 0 |
| ***nad3*** | 266 | C | T | 0.846 | 0 | 2 | 0 | 11 | 0 | 0 | 0 |
| ***nad3*** | 275 | C | T | 0.905 | 0 | 2 | 0 | 19 | 0 | 0 | 0 |
| ***nad3*** | 317 | C | T | 0.903 | 0 | 3 | 0 | 28 | 0 | 0 | 0 |
| ***nad3*** | 344 | C | T | 0.948 | 0 | 3 | 0 | 55 | 0 | 0 | 0 |
| ***nad3*** | 349 | C | T | 0.923 | 0 | 4 | 0 | 48 | 0 | 0 | 0 |
| ***rps12m*** | 71 | C | T | 1.000 | 0 | 0 | 0 | 101 | 0 | 0 | 0 |
| ***rps12m*** | 100 | C | T | 1.000 | 0 | 0 | 0 | 70 | 0 | 0 | 0 |
| ***rps12m*** | 104 | C | T | 1.000 | 0 | 0 | 0 | 70 | 0 | 0 | 0 |
| ***rps12m*** | 146 | C | T | 1.000 | 1 | 0 | 0 | 56 | 0 | 0 | 0 |
| ***rps12m*** | 196 | C | T | 1.000 | 0 | 0 | 0 | 82 | 0 | 0 | 0 |
| ***rps12m*** | 221 | C | T | 0.978 | 0 | 1 | 0 | 45 | 0 | 0 | 0 |
| ***rps12m*** | 284 | C | T | 0.933 | 0 | 2 | 0 | 28 | 0 | 0 | 0 |
| ***nad4*** | 29 | C | T | 0.868 | 0 | 5 | 0 | 33 | 0 | 0 | 0 |
| ***nad4*** | 44 | C | T | 0.921 | 0 | 3 | 0 | 35 | 0 | 0 | 0 |
| ***nad4*** | 77 | C | T | 0.714 | 0 | 2 | 0 | 5 | 0 | 0 | 0 |
| ***nad4*** | 107 | C | T | 0.769 | 0 | 3 | 0 | 10 | 0 | 0 | 0 |
| ***nad4*** | 158 | C | T | 1.000 | 0 | 0 | 0 | 48 | 0 | 0 | 0 |
| ***nad4*** | 164 | C | T | 0.787 | 0 | 10 | 0 | 37 | 0 | 0 | 0 |
| ***nad4*** | 166 | C | T | 0.830 | 0 | 8 | 0 | 39 | 0 | 0 | 0 |
| ***nad4*** | 403 | C | T | 0.952 | 0 | 1 | 0 | 20 | 0 | 0 | 0 |
| ***nad4*** | 416 | C | T | 0.967 | 0 | 1 | 0 | 29 | 0 | 0 | 0 |
| ***nad4*** | 433 | C | T | 0.943 | 0 | 2 | 0 | 33 | 0 | 0 | 0 |
| ***nad4*** | 436 | C | T | 1.000 | 0 | 0 | 0 | 34 | 0 | 0 | 0 |
| ***nad4*** | 437 | C | T | 0.971 | 0 | 1 | 0 | 33 | 0 | 0 | 0 |
| ***nad4*** | 608 | C | T | 0.926 | 0 | 2 | 0 | 25 | 0 | 0 | 0 |
| ***nad4*** | 659 | C | T | 0.846 | 0 | 2 | 0 | 11 | 0 | 0 | 0 |
| ***nad4*** | 767 | C | T | 0.714 | 0 | 6 | 0 | 15 | 0 | 0 | 0 |
| ***nad4*** | 770 | C | T | 0.833 | 0 | 3 | 0 | 15 | 0 | 0 | 0 |
| ***nad4*** | 784 | C | T | 0.550 | 0 | 9 | 0 | 11 | 0 | 0 | 0 |
| ***nad4*** | 836 | C | T | 0.860 | 0 | 8 | 0 | 49 | 0 | 0 | 0 |
| ***nad4*** | 856 | C | T | 0.576 | 1 | 25 | 0 | 34 | 0 | 0 | 0 |
| ***nad4*** | 857 | C | T | 0.667 | 0 | 20 | 0 | 40 | 0 | 0 | 0 |
| ***nad4*** | 896 | C | T | 0.917 | 0 | 2 | 0 | 22 | 0 | 0 | 0 |
| ***nad4*** | 977 | C | T | 0.905 | 0 | 2 | 5 | 19 | 0 | 0 | 0 |
| ***nad4*** | 1006 | C | T | 0.706 | 0 | 5 | 0 | 12 | 0 | 0 | 0 |
| ***nad4*** | 1007 | C | T | 0.562 | 0 | 7 | 0 | 9 | 0 | 0 | 0 |
| ***nad4*** | 1009 | C | T | 0.529 | 0 | 8 | 0 | 9 | 0 | 0 | 0 |
| ***nad4*** | 1010 | C | T | 0.529 | 0 | 8 | 0 | 9 | 0 | 0 | 0 |
| ***nad4*** | 1033 | C | T | 0.867 | 0 | 4 | 0 | 26 | 0 | 0 | 0 |
| ***nad4*** | 1129 | C | T | 1.000 | 0 | 0 | 0 | 20 | 0 | 0 | 0 |
| ***nad4*** | 1142 | C | T | 0.667 | 0 | 6 | 0 | 12 | 0 | 0 | 0 |
| ***nad4*** | 1148 | C | T | 0.773 | 0 | 5 | 0 | 17 | 0 | 0 | 0 |
| ***nad4*** | 1151 | C | T | 0.667 | 0 | 7 | 0 | 14 | 0 | 0 | 0 |
| ***nad4*** | 1172 | C | T | 0.974 | 0 | 1 | 0 | 38 | 0 | 0 | 0 |
| ***nad4*** | 1205 | C | T | 0.957 | 0 | 2 | 0 | 45 | 0 | 0 | 0 |
| ***nad4*** | 1211 | C | T | 0.905 | 0 | 4 | 0 | 38 | 0 | 0 | 0 |
| ***nad4*** | 1355 | C | T | 0.988 | 0 | 2 | 0 | 164 | 0 | 0 | 0 |
| ***nad4*** | 1373 | C | T | 0.993 | 0 | 1 | 1 | 137 | 0 | 0 | 0 |
| ***nad4*** | 1405 | C | T | 1.000 | 0 | 0 | 0 | 83 | 0 | 0 | 0 |
| ***nad4*** | 1417 | C | T | 1.000 | 0 | 0 | 0 | 55 | 0 | 0 | 0 |
| ***nad4*** | 1433 | C | T | 0.979 | 0 | 1 | 0 | 47 | 0 | 0 | 0 |
| ***nad4*** | 1438 | C | T | 1.000 | 0 | 0 | 0 | 42 | 0 | 0 | 0 |
| ***matR*** | 32 | C | T | 0.741 | 0 | 7 | 0 | 20 | 0 | 0 | 0 |
| ***matR*** | 96 | C | T | 0.467 | 0 | 16 | 0 | 14 | 0 | 0 | 0 |
| ***matR*** | 147 | C | T | 0.547 | 0 | 29 | 0 | 35 | 0 | 0 | 0 |
| ***matR*** | 236 | C | T | 0.965 | 0 | 2 | 0 | 55 | 0 | 0 | 0 |
| ***matR*** | 326 | C | T | 0.733 | 0 | 4 | 0 | 11 | 0 | 0 | 0 |
| ***matR*** | 404 | C | T | 0.970 | 0 | 1 | 0 | 32 | 0 | 0 | 0 |
| ***matR*** | 1058 | C | T | 0.867 | 0 | 2 | 0 | 13 | 0 | 0 | 0 |
| ***matR*** | 1664 | C | T | 0.778 | 0 | 12 | 0 | 42 | 0 | 0 | 0 |
| ***matR*** | 1685 | C | T | 0.731 | 0 | 7 | 0 | 19 | 0 | 0 | 0 |
| ***matR*** | 1705 | C | T | 0.873 | 0 | 8 | 1 | 55 | 0 | 0 | 0 |
| ***matR*** | 1719 | C | T | 0.887 | 0 | 8 | 0 | 63 | 0 | 0 | 0 |
| ***matR*** | 1741 | C | T | 0.872 | 1 | 16 | 0 | 109 | 0 | 0 | 0 |
| ***matR*** | 1811 | C | T | 0.938 | 0 | 2 | 0 | 30 | 0 | 0 | 0 |
| ***matR*** | 1829 | C | T | 0.917 | 0 | 2 | 0 | 22 | 0 | 0 | 0 |
| ***matR*** | 2001 | C | T | 0.556 | 0 | 4 | 0 | 5 | 0 | 0 | 0 |
| ***rpl5*** | 35 | C | T | 0.562 | 0 | 7 | 0 | 9 | 0 | 0 | 0 |
| ***rpl5*** | 47 | C | T | 0.927 | 0 | 3 | 0 | 38 | 0 | 0 | 0 |
| ***rpl5*** | 64 | C | T | 0.788 | 0 | 11 | 0 | 41 | 0 | 0 | 0 |
| ***rpl5*** | 92 | C | T | 0.959 | 0 | 2 | 0 | 47 | 0 | 0 | 0 |
| ***rpl5*** | 172 | C | T | 0.900 | 0 | 3 | 0 | 27 | 0 | 0 | 0 |
| ***rpl5*** | 320 | C | T | 0.722 | 0 | 5 | 0 | 13 | 0 | 0 | 0 |
| ***rpl5*** | 332 | C | T | 0.522 | 0 | 11 | 0 | 12 | 0 | 0 | 0 |
| ***rpl5*** | 515 | C | T | 0.762 | 0 | 5 | 0 | 16 | 0 | 0 | 0 |
| ***rpl5*** | 518 | C | T | 0.950 | 0 | 1 | 0 | 19 | 0 | 0 | 0 |
| ***rps14m*** | 194 | C | T | 0.951 | 0 | 2 | 0 | 39 | 0 | 0 | 0 |
| ***rps14m*** | 271 | C | T | 0.967 | 0 | 1 | 0 | 29 | 0 | 0 | 0 |
| ***cob*** | 286 | C | T | 0.938 | 0 | 1 | 0 | 15 | 0 | 0 | 0 |
| ***cob*** | 298 | C | T | 0.941 | 0 | 1 | 0 | 16 | 0 | 0 | 0 |
| ***cob*** | 325 | C | T | 1.000 | 0 | 0 | 0 | 21 | 0 | 0 | 0 |
| ***cob*** | 358 | C | T | 1.000 | 0 | 0 | 0 | 20 | 0 | 0 | 0 |
| ***cob*** | 407 | C | T | 0.976 | 0 | 1 | 0 | 40 | 0 | 0 | 0 |
| ***cob*** | 568 | C | T | 0.966 | 0 | 1 | 0 | 28 | 0 | 0 | 0 |
| ***cob*** | 808 | C | T | 0.969 | 0 | 3 | 0 | 93 | 0 | 0 | 0 |
| ***cob*** | 853 | C | T | 1.000 | 0 | 0 | 0 | 29 | 0 | 0 | 0 |
| ***cob*** | 908 | C | T | 0.978 | 0 | 1 | 0 | 44 | 0 | 0 | 0 |
| ***cob*** | 982 | C | T | 0.970 | 0 | 1 | 0 | 32 | 0 | 0 | 0 |
| ***cob*** | 1015 | C | T | 1.000 | 0 | 0 | 0 | 25 | 0 | 0 | 0 |
| ***cob*** | 1081 | C | T | 1.000 | 0 | 0 | 0 | 69 | 0 | 0 | 0 |
| ***cob*** | 1084 | C | T | 1.000 | 0 | 0 | 0 | 70 | 0 | 0 | 0 |
| ***cob*** | 1124 | C | T | 1.000 | 0 | 0 | 0 | 9 | 0 | 0 | 0 |
| ***nad7*** | 38 | C | T | 0.818 | 0 | 2 | 0 | 9 | 0 | 0 | 0 |
| ***nad7*** | 77 | C | T | 0.960 | 0 | 1 | 0 | 24 | 0 | 0 | 0 |
| ***nad7*** | 83 | C | T | 0.909 | 0 | 2 | 0 | 20 | 0 | 0 | 0 |
| ***nad7*** | 137 | C | T | 1.000 | 0 | 0 | 0 | 21 | 0 | 0 | 0 |
| ***nad7*** | 200 | C | T | 1.000 | 0 | 0 | 0 | 10 | 0 | 0 | 0 |
| ***nad7*** | 224 | C | T | 0.955 | 0 | 1 | 0 | 21 | 0 | 0 | 0 |
| ***nad7*** | 244 | C | T | 1.000 | 0 | 0 | 0 | 12 | 0 | 0 | 0 |
| ***nad7*** | 251 | C | T | 1.000 | 0 | 0 | 0 | 11 | 0 | 0 | 0 |
| ***nad7*** | 316 | C | T | 1.000 | 0 | 0 | 0 | 17 | 0 | 0 | 0 |
| ***nad7*** | 335 | C | T | 0.947 | 0 | 1 | 0 | 18 | 0 | 0 | 0 |
| ***nad7*** | 383 | C | T | 0.929 | 0 | 3 | 0 | 39 | 0 | 0 | 0 |
| ***nad7*** | 392 | C | T | 0.915 | 0 | 5 | 0 | 54 | 0 | 0 | 0 |
| ***nad7*** | 578 | C | T | 0.911 | 0 | 8 | 0 | 82 | 0 | 0 | 0 |
| ***nad7*** | 679 | C | T | 0.562 | 0 | 14 | 0 | 18 | 0 | 0 | 0 |
| ***nad7*** | 698 | C | T | 0.947 | 0 | 1 | 0 | 18 | 0 | 0 | 0 |
| ***nad7*** | 724 | C | T | 0.952 | 0 | 1 | 0 | 20 | 0 | 0 | 0 |
| ***nad7*** | 734 | C | T | 0.833 | 0 | 4 | 0 | 20 | 0 | 0 | 0 |
| ***nad7*** | 739 | C | T | 0.857 | 0 | 3 | 0 | 18 | 0 | 0 | 0 |
| ***nad7*** | 740 | C | T | 0.857 | 0 | 3 | 0 | 18 | 0 | 0 | 0 |
| ***nad7*** | 769 | C | T | 0.905 | 0 | 2 | 0 | 19 | 0 | 0 | 0 |
| ***nad7*** | 789 | C | T | 0.500 | 0 | 8 | 0 | 8 | 0 | 0 | 0 |
| ***nad7*** | 807 | G | A | 1.000 | 20 | 0 | 0 | 0 | 0 | 0 | 0 |
| ***nad7*** | 836 | C | T | 0.952 | 0 | 1 | 0 | 20 | 0 | 0 | 0 |
| ***nad7*** | 926 | C | T | 0.725 | 0 | 14 | 0 | 37 | 0 | 0 | 0 |
| ***nad7*** | 963 | C | T | 0.667 | 0 | 8 | 0 | 16 | 0 | 0 | 0 |
| ***nad7*** | 973 | C | T | 1.000 | 0 | 0 | 0 | 18 | 0 | 0 | 0 |
| ***nad7*** | 1057 | C | T | 0.983 | 0 | 2 | 0 | 116 | 0 | 0 | 0 |
| ***nad7*** | 1079 | C | T | 0.987 | 0 | 1 | 0 | 74 | 0 | 0 | 0 |
| ***nad7*** | 1088 | C | T | 0.864 | 0 | 8 | 0 | 51 | 0 | 0 | 0 |
| ***nad7*** | 1103 | C | T | 0.976 | 0 | 1 | 0 | 40 | 0 | 0 | 0 |
| ***nad7*** | 1124 | C | T | 0.929 | 0 | 1 | 0 | 13 | 0 | 0 | 0 |
| ***nad7*** | 1166 | C | T | 0.943 | 0 | 4 | 0 | 66 | 0 | 0 | 0 |
| ***rps1*** | 158 | C | T | 1.000 | 0 | 0 | 0 | 3 | 0 | 0 | 0 |
| ***rps1*** | 209 | C | T | 1.000 | 0 | 0 | 0 | 10 | 0 | 0 | 0 |
| ***rps1*** | 526 | C | T | 0.857 | 0 | 2 | 0 | 12 | 0 | 0 | 0 |
