# Supplementary material for: Capturing the Biofuel Wellhead and Powerhouse: The Chloroplast and Mitochondrial Genomes of the Leguminous Feedstock Tree Pongamia pinnata
Source: PLoS One. 2012 Dec 14;7(12):e51687. doi: 10.1371/journal.pone.0051687 (PMC3522722; doi:10.1371/journal.pone.0051687)
Supplement: Table S5 — Transcription (RPKM) of Pongamia protein-coding genes in leaf samples that have been treated with either fresh- or salt-water. Cells highlighted in red indicate a minimum 2.5-fold change. Asterisks represent genes that have a 2.5-fold change in both leaf and root samples. (DOCX) [file pone.0051687.s015.docx]

**Table S5**

| Chloroplast  genes | Freshwater treatment | | Saltwater treatment |  | Chloroplast  genes | Freshwater treatment | | Saltwater treatment |  | Mitochondrial genes | Freshwater treatment | | Saltwater treatment |  |
| --- | --- | --- | --- | --- | --- | --- | --- | --- | --- | --- | --- | --- | --- | --- |
| *accD* | 333.31 | 465.08 | |  | ***psbJ*** | 432.17 | 435.77 | |  | ***atp1*** | 1326.08 | 1944.77 | |  |
| *atpA* | 7028.85 | 8755.51 | |  | ***psbK*** | 23701.44 | 42413.29 | |  | ***atp4*** | 87.99 | 1045.39 | |  |
| *atpB* | 235316.76 | 199986.84 | |  | ***psbL*** | 278.66 | 389.89 | |  | ***atp6*** | 11.96 | 82.60 | |  |
| *atpE* | 494832.21 | 368783.42 | |  | ***psbM*** | 47.25 | 21.72 | |  | ***atp8*** | 567.89 | 556.04 | |  |
| *atpF* | 13080.72 | 10943.95 | |  | ***psbN*** | 977.23 | 1490.32 | |  | ***atp9*** | 1055.26 | 1649.81 | |  |
| *atpH* | 24051.64 | 19512.35 | |  | ***psbT*** | 1056.57 | 1119.30 | |  | ***ccmB*** | 13.70 | 66.11 | |  |
| *atpI* | 84153.45 | 118561.79 | |  | ***psbZ*** | 2396.27 | 2757.53 | |  | ***ccmC*** | 0.00 | 19.24 | |  |
| *ccsA* | 111.22 | 338.62 | |  | ***rbcL*** | 1760.48 | 4980.16 | |  | ***ccmFc*** | 16.99 | 44.85 | |  |
| *cemA* | 1325.07 | 2285.80 | |  | ***rpl2*** | 52943.71 | 51533.97 | |  | ***ccmFn**** | 0.82 | 12.19 | |  |
| *clpP* | 22494.34 | 20853.72 | |  | ***rpl14*** | 20148.67 | 16857.55 | |  | ***cob*** | 13.83 | 179.43 | |  |
| *matK** | 28.83 | 110.00 | |  | ***rpl16*** | 22555.15 | 17060.22 | |  | ***cox1*** | 23.71 | 132.11 | |  |
| *ndhA* | 4739.29 | 8609.93 | |  | ***rpl20*** | 3276.87 | 5317.62 | |  | ***cox2*** | 47.97 | 749.36 | |  |
| *ndhB* | 571.44 | 1959.47 | |  | ***rpl23*** | 9937.68 | 9843.22 | |  | ***cox3*** | 17.76 | 130.05 | |  |
| *ndhC* | 4701.62 | 6553.50 | |  | ***rpl32*** | 12810.12 | 14772.70 | |  | ***matR*** | 25.74 | 84.82 | |  |
| *ndhD* | 652.89 | 1922.80 | |  | ***rpl33*** | 1717.24 | 3310.63 | |  | ***mttB**** | 10.69 | 33.63 | |  |
| *ndhE** | 896.37 | 3944.89 | |  | ***rpl36*** | 795.80 | 820.30 | |  | ***nad1*** | 13.77 | 44.31 | |  |
| *ndhF* | 8187.14 | 9881.38 | |  | ***rpoA*** | 2768.54 | 3503.78 | |  | ***nad2*** | 16.43 | 71.52 | |  |
| *ndhG* | 646.02 | 1927.55 | |  | ***rpoB*** | 130.52 | 307.08 | |  | ***nad3*** | 25.81 | 65.49 | |  |
| *ndhH* | 15042.80 | 20761.57 | |  | ***rpoC1*** | 561.49 | 913.73 | |  | ***nad4L*** | 4.68 | 116.68 | |  |
| *ndhI* | 3044.24 | 4535.35 | |  | ***rpoC2*** | 4943.73 | 4883.78 | |  | ***nad4*** | 30.48 | 125.31 | |  |
| *ndhJ* | 10298.52 | 11283.49 | |  | ***rps11*** | 5557.07 | 4433.60 | |  | ***nad5*** | 12.69 | 48.79 | |  |
| *ndhK* | 8270.92 | 9841.61 | |  | ***rps12*** | 50222.36 | 50691.48 | |  | ***nad6*** | 5.73 | 53.51 | |  |
| *petA* | 3077.17 | 5198.81 | |  | ***rps14*** | 12147.11 | 9102.68 | |  | ***nad7*** | 29.91 | 115.49 | |  |
| *petB* | 756.88 | 2551.87 | |  | ***rps15*** | 18626.63 | 19369.18 | |  | ***nad9*** | 23.50 | 83.59 | |  |
| *petD* | 1416.05 | 5909.89 | |  | ***rps16*** | 1638.02 | 1731.75 | |  | ***rpl2*** | - | - | |  |
| *petG* | 55.95 | 80.03 | |  | ***rps18*** | 2952.01 | 3912.13 | |  | ***rpl5*** | 13.90 | 152.46 | |  |
| *petL* | 0.00 | 5.94 | |  | ***rps19*** | 10036.91 | 6358.16 | |  | ***rpl10*** | - | - | |  |
| *petN* | 70.88 | 82.36 | |  | ***rps2*** | 19379.67 | 16388.56 | |  | ***rpl16*** | 748.13 | 994.29 | |  |
| *psaA* | 3515.79 | 5567.47 | |  | ***rps3*** | 51850.37 | 55231.51 | |  | ***rps1*** | 3.46 | 16.69 | |  |
| *psaB* | 1362.87 | 4181.29 | |  | ***rps4*** | 1981.24 | 2414.46 | |  | ***rps2*** | - | - | |  |
| *psaC* | 1947.64 | 5576.94 | |  | ***rps7*** | 17865.82 | 15495.61 | |  | ***rps3*** | 222.90 | 362.01 | |  |
| *psaI** | 54.00 | 233.52 | |  | ***rps8*** | 10151.85 | 10500.33 | |  | ***rps4*** | 3.29 | 63.00 | |  |
| *psaJ* | 1118.26 | 1921.82 | |  | ***ycf1*** | 10172.69 | 9875.81 | |  | ***rps7*** | - | - | |  |
| *psbA* | 1256.01 | 16567.27 | |  | ***ycf2*** | 476.74 | 579.11 | |  | ***rps10*** | 63.85 | 356.17 | |  |
| *psbB* | 3919.75 | 4447.05 | |  | ***ycf3*** | 2973.42 | 4020.72 | |  | ***rps11*** | - | - | |  |
| *psbC* | 1746.47 | 6637.23 | |  | ***ycf4*** | 5867.74 | 7514.57 | |  | ***rps12*** | 50.63 | 152.36 | |  |
| *psbD* | 1427.52 | 3769.73 | |  | ***-*** | - | - | |  | ***rps13*** | - | - | |  |
| *psbE** | 683.44 | 4283.37 | |  | ***-*** | - | - | |  | ***rps14*** | 30.41 | 154.31 | |  |
| *psbF* | 218.53 | 332.62 | |  | **-** | - | - | |  | ***rps19*** | - | - | |  |
| *psbH* | 399.07 | 503.43 | |  | **-** | - | - | |  | ***sdh3*** | 24.61 | 72.86 | |  |
| *psbI* | 2394.45 | 2311.67 | |  | **-** | - | - | |  | ***sdh4*** | - | - | |  |
|  |  |  | |  |  |  |  | |  |  |  |  | |  |
| Total reads mapped: | | | | |  |  |  | |  |  | 1410921 | 1753736 | |  |
