# Supplementary material for: Capturing the Biofuel Wellhead and Powerhouse: The Chloroplast and Mitochondrial Genomes of the Leguminous Feedstock Tree Pongamia pinnata
Source: PLoS One. 2012 Dec 14;7(12):e51687. doi: 10.1371/journal.pone.0051687 (PMC3522722; doi:10.1371/journal.pone.0051687)
Supplement: Table S6 — Transcription (RPKM) of Pongamia protein-coding genes in root samples that have been treated with either fresh- or salt-water. A key to this table can be found in the Table S5 legend. (DOCX) [file pone.0051687.s016.docx]

**Table S6**

| Chloroplast  genes | Freshwater treatment | | Saltwater treatment |  | Chloroplast  genes | Freshwater treatment | | Saltwater treatment |  | Mitochondrial genes | Freshwater treatment | | Saltwater treatment |  |
| --- | --- | --- | --- | --- | --- | --- | --- | --- | --- | --- | --- | --- | --- | --- |
| *accD* | 961.35 | 920.95 | |  | ***psbJ*** | 71.34 | 258.66 | |  | ***atp1*** | 55484.57 | 46962.59 | |  |
| *atpA* | 6434.03 | 6122.33 | |  | ***psbK*** | 6510.66 | 5302.56 | |  | ***atp4*** | 2641.53 | 3372.72 | |  |
| *atpB* | 95413.88 | 90359.66 | |  | ***psbL*** | 225.01 | 181.28 | |  | ***atp6*** | 555.39 | 432.56 | |  |
| *atpE* | 253696.11 | 237718.44 | |  | ***psbM*** | 83.57 | 0.00 | |  | ***atp8*** | 33992.70 | 31398.21 | |  |
| *atpF* | 10814.89 | 10127.42 | |  | ***psbN*** | 132.96 | 241.03 | |  | ***atp9*** | 32214.88 | 34926.22 | |  |
| *atpH* | 15017.79 | 15864.58 | |  | ***psbT*** | 162.50 | 196.39 | |  | ***ccmB*** | 310.88 | 375.71 | |  |
| *atpI* | 21206.83 | 25016.13 | |  | ***psbZ*** | 835.74 | 897.79 | |  | ***ccmC*** | 47.37 | 28.62 | |  |
| *ccsA* | 180.00 | 174.03 | |  | ***rbcL*** | 2630.11 | 2205.69 | |  | ***ccmFc*** | 512.71 | 436.92 | |  |
| *cemA* | 928.39 | 952.92 | |  | ***rpl2*** | 131405.23 | 131683.56 | |  | ***ccmFn**** | 15.21 | 104.15 | |  |
| *clpP* | 41634.13 | 33268.88 | |  | ***rpl14*** | 33935.68 | 33022.47 | |  | ***cob*** | 595.44 | 1025.43 | |  |
| *matK** | 23.03 | 118.30 | |  | ***rpl16*** | 38219.61 | 36961.99 | |  | ***cox1*** | 875.31 | 1251.99 | |  |
| *ndhA* | 1955.40 | 2837.72 | |  | ***rpl20*** | 3572.62 | 6449.43 | |  | ***cox2*** | 2857.84 | 1747.21 | |  |
| *ndhB* | 309.11 | 594.94 | |  | ***rpl23*** | 15807.88 | 15982.91 | |  | ***cox3*** | 835.74 | 584.74 | |  |
| *ndhC* | 4230.49 | 2979.95 | |  | ***rpl32*** | 19554.70 | 22061.29 | |  | ***matR*** | 1117.64 | 1355.98 | |  |
| *ndhD* | 451.37 | 722.59 | |  | ***rpl33*** | 3143.37 | 1793.90 | |  | ***mttB**** | 72.22 | 450.97 | |  |
| *ndhE** | 286.77 | 727.80 | |  | ***rpl36*** | 1077.66 | 2046.60 | |  | ***nad1*** | 349.93 | 292.78 | |  |
| *ndhF* | 21162.89 | 31428.37 | |  | ***rpoA*** | 7829.41 | 9652.57 | |  | ***nad2*** | 747.72 | 831.35 | |  |
| *ndhG* | 727.14 | 599.16 | |  | ***rpoB*** | 263.45 | 262.58 | |  | ***nad3*** | 958.64 | 594.12 | |  |
| *ndhH* | 5449.26 | 6666.34 | |  | ***rpoC1*** | 1161.49 | 1656.57 | |  | ***nad4L*** | 405.46 | 245.00 | |  |
| *ndhI* | 898.67 | 1149.95 | |  | ***rpoC2*** | 8958.98 | 9173.24 | |  | ***nad4*** | 1444.85 | 1667.74 | |  |
| *ndhJ* | 9400.73 | 12028.04 | |  | ***rps11*** | 9432.32 | 8991.30 | |  | ***nad5*** | 528.26 | 532.89 | |  |
| *ndhK* | 7092.67 | 7242.15 | |  | ***rps12*** | 91675.72 | 99542.32 | |  | ***nad6*** | 340.79 | 411.85 | |  |
| *petA* | 1585.56 | 2598.97 | |  | ***rps14*** | 1824.55 | 3395.04 | |  | ***nad7*** | 1288.52 | 1485.61 | |  |
| *petB* | 433.35 | 785.57 | |  | ***rps15*** | 8313.39 | 6884.03 | |  | ***nad9*** | 597.27 | 703.31 | |  |
| *petD* | 708.56 | 1646.76 | |  | ***rps16*** | 4095.11 | 7109.36 | |  | ***rpl2*** | - | - | |  |
| *petG* | 153.95 | 93.03 | |  | ***rps18*** | 8775.24 | 7310.33 | |  | ***rpl5*** | 766.46 | 472.60 | |  |
| *petL* | 0.00 | 0.00 | |  | ***rps19*** | 26482.98 | 20145.94 | |  | ***rpl10*** | - | - | |  |
| *petN* | 97.50 | 0.00 | |  | ***rps2*** | 16390.32 | 17078.58 | |  | ***rpl16*** | 25051.89 | 16496.87 | |  |
| *psaA* | 323.28 | 371.86 | |  | ***rps3*** | 138387.00 | 139787.54 | |  | ***rps1*** | 228.30 | 258.66 | |  |
| *psaB* | 179.09 | 144.29 | |  | ***rps4*** | 3084.37 | 3097.54 | |  | ***rps2*** | - | - | |  |
| *psaC* | 642.09 | 1034.65 | |  | ***rps7*** | 56176.54 | 42080.61 | |  | ***rps3*** | 8418.65 | 6083.27 | |  |
| *psaI** | 0.00 | 202.00 | |  | ***rps8*** | 16835.46 | 25269.01 | |  | ***rps4*** | 179.25 | 216.63 | |  |
| *psaJ* | 130.00 | 471.34 | |  | ***ycf1*** | 11200.84 | 11504.67 | |  | ***rps7*** | - | - | |  |
| *psbA* | 570.14 | 1328.14 | |  | ***ycf2*** | 460.64 | 640.20 | |  | ***rps10*** | 1976.41 | 2866.25 | |  |
| *psbB* | 1229.80 | 2146.03 | |  | ***ycf3*** | 9121.40 | 8157.79 | |  | ***rps11*** | - | - | |  |
| *psbC* | 197.47 | 171.53 | |  | ***ycf4*** | 2940.00 | 3138.25 | |  | ***rps12*** | 1996.48 | 1318.63 | |  |
| *psbD* | 272.68 | 349.51 | |  | ***-*** | - | - | |  | ***rps13*** | - | - | |  |
| *psbE** | 104.47 | 631.26 | |  | ***-*** | - | - | |  | ***rps14*** | 1505.98 | 770.01 | |  |
| *psbF* | 146.25 | 265.13 | |  | **-** | - | - | |  | ***rps19*** | - | - | |  |
| *psbH* | 237.17 | 382.17 | |  | **-** | - | - | |  | ***sdh3*** | 1048.15 | 1060.51 | |  |
| *psbI* | 632.45 | 191.08 | |  | **-** | - | - | |  | ***sdh4*** | - | - | |  |
|  |  |  | |  |  |  |  | |  |  |  |  | |  |
| Total reads mapped: | | | | |  |  |  | |  |  | 113957 | 94294 | |  |
