# Supplementary material for: Capturing the Biofuel Wellhead and Powerhouse: The Chloroplast and Mitochondrial Genomes of the Leguminous Feedstock Tree Pongamia pinnata
Source: PLoS One. 2012 Dec 14;7(12):e51687. doi: 10.1371/journal.pone.0051687 (PMC3522722; doi:10.1371/journal.pone.0051687)
Supplement: Table S7 — Approximate nucleotide divergence percentages of seventy-seven protein-coding chloroplast and forty-one protein-coding mitochondrial genes common to three legume species, L. japonicus, V. radiata and Pongamia. Bracketed values represent the corresponding divergence at the amino-acid level. The list of forty-one mitochondrial genes includes seventeen genes (fifteen ribosomal protein and two respiratory; listed last) known to have been lost frequently during angiosperm evolution. For clarity, the duplicates and pseudogenes have been ignored. (DOCX) [file pone.0051687.s017.docx]

**Table S7**

| Chloroplast genes | *L. japonicus –* Pongamia | | Pongamia *– V. radiata* | | *V. radiata – L. japonicus* | |  | Chloroplast genes | *L. japonicus –* Pongamia | | Pongamia *– V. radiata* | | *V. radiata – L. japonicus* | |  | Mitochondrial genes | *L. japonicus –* Pongamia | | Pongamia *– V. radiata* | | *V. radiata – L. japonicus* | |  |
| --- | --- | --- | --- | --- | --- | --- | --- | --- | --- | --- | --- | --- | --- | --- | --- | --- | --- | --- | --- | --- | --- | --- | --- |
| *accD* | 11.9 | [18.0] | 13.4 | [22.7] | 17.0 | [28.3] |  | ***psbJ*** | 2.4 | [0.0] | 2.4 | [0.0] | 4.9 | [0.0] |  | ***atp1*** | 2.0 | [2.6] | 2.7 | [3.7] | 2.6 | [2.8] |  |
| *atpA* | 6.5 | [3.9] | 5.5 | [3.9] | 6.5 | [2.9] |  | ***psbK*** | 9.1 | [16.4] | 8.1 | [14.8] | 11.3 | [19.7] |  | ***atp4*** | 0.9 | [2.1] | 1.5 | [3.1] | 1.7 | [3.1] |  |
| *atpB* | 6.7 | [3.2] | 6.5 | [4.4] | 8.0 | [5.2] |  | ***psbL*** | 4.3 | [2.6] | 5.1 | [0.0] | 5.1 | [2.6] |  | ***atp6*** | 2.2 | [2.5] | 1.7 | [1.3] | 2.2 | [2.9] |  |
| *atpE* | 10.7 | [12.8] | 8.7 | [10.5] | 10.9 | [11.3] |  | ***psbM*** | 4.8 | [0.0] | 4.8 | [2.9] | 7.6 | [2.9] |  | ***atp8*** | 1.4 | [1.9] | 2.5 | [3.8] | 3.1 | [4.4] |  |
| *atpF* | 7.7 | [10.3] | 8.7 | [11.7] | 10.9 | [13.3] |  | ***psbN*** | 5.3 | [0.0] | 6.8 | [2.3] | 5.3 | [2.3] |  | ***atp9*** | 1.8 | [1.4] | 0.4 | [1.4] | 1.3 | [2.7] |  |
| *atpH* | 5.7 | [1.2] | 6.1 | [1.2] | 5.7 | [0.0] |  | ***psbT*** | 3.9 | [0.0] | 11.1 | [2.9] | 10.7 | [0.0] |  | ***ccmB*** | 1.0 | [2.4] | 1.9 | [5.3] | 1.9 | [5.8] |  |
| *atpI* | 6.9 | [5.7] | 4.8 | [4.5] | 8.7 | [5.3] |  | ***psbZ*** | 5.8 | [3.2] | 4.8 | [6.5] | 7.9 | [9.7] |  | ***ccmC*** | 0.7 | [0.8] | 0.9 | [1.6] | 1.1 | [0.8] |  |
| *ccsA* | 12.3 | [16.3] | 12.8 | [18.2] | 13.9 | [19.2] |  | ***rbcL*** | 6.5 | [3.4] | 6.4 | [4.6] | 6.9 | [3.6] |  | ***ccmFc*** | 13.9 | [14.9] | 1.6 | [2.9] | 13.8 | [14.9] |  |
| *cemA* | 9.7 | [12.2] | 10.0 | [13.5] | 12.9 | [17.2] |  | ***rpl2*** | 4.1 | [5.8] | 3.5 | [4.0] | 5.3 | [5.8] |  | ***ccmFn*** | 0.9 | [1.6] | 0.8 | [1.2] | 0.5 | [1.0] |  |
| *clpP* | 10.7 | [14.3] | 13.4 | [19.4] | 13.4 | [15.3] |  | ***rpl14*** | 7.0 | [6.6] | 6.2 | [5.7] | 7.6 | [6.6] |  | ***cob*** | 1.4 | [1.8] | 1.1 | [1.5] | 2.2 | [2.8] |  |
| *matK* | 14.8 | [25.7] | 16.0 | [29.3] | 18.3 | [30.6] |  | ***rpl16*** | 7.8 | [4.4] | 6.1 | [3.0] | 8.3 | [5.2] |  | ***cox1*** | 0.5 | [0.2] | 0.6 | [0.2] | 0.9 | [0.4] |  |
| *ndhA* | 8.7 | [8.5] | 8.4 | [8.2] | 9.2 | [10.5] |  | ***rpl20*** | 13.0 | [16.5] | 10.8 | [16.0] | 15.2 | [23.1] |  | ***cox2*** | 2.9 | [3.1] | - | - | - | - |  |
| *ndhB* | 3.1 | [3.1] | 2.5 | [2.9] | 3.2 | [2.7] |  | ***rpl23*** | 2.5 | [5.4] | 1.4 | [2.2] | 3.2 | [5.4] |  | ***cox3*** | 1.4 | [1.1] | 0.6 | [0.0] | 1.5 | [1.1] |  |
| *ndhC* | 8.0 | [9.2] | 5.2 | [5.0] | 10.2 | [10.0] |  | ***rpl32*** | 7.2 | [4.0] | 11.7 | [18.9] | 13.7 | [18.0] |  | ***matR*** | 1.7 | [2.6] | 1.5 | [3.7] | 1.4 | [2.6] |  |
| *ndhD* | 9.2 | [10.5] | 8.5 | [9.4] | 10.6 | [12.5] |  | ***rpl33*** | 8.0 | [9.1] | - | - | - | - |  | ***mttB*** | 1.0 | [1.2] | 1.1 | [1.7] | 1.8 | [2.1] |  |
| *ndhE* | 6.9 | [7.9] | 5.6 | [8.9] | 8.5 | [7.9] |  | ***rpl36*** | 9.6 | [5.4] | 10.5 | [8.1] | 13.2 | [8.1] |  | ***nad1*** | 0.6 | [0.6] | 0.1 | [0.3] | 0.7 | [0.9] |  |
| *ndhF* | 13.0 | [16.8] | 13.6 | [19.2] | 16.3 | [20.2] |  | ***rpoA*** | 9.9 | [14.1] | 9.6 | [14.4] | 11.7 | [17.7] |  | ***nad2*** | 0.7 | [0.8] | 0.6 | [0.8] | 0.9 | [0.8] |  |
| *ndhG* | 8.3 | [9.7] | 10.0 | [13.6] | 10.0 | [13.1] |  | ***rpoB*** | 9.4 | [9.6] | 7.1 | [7.1] | 9.7 | [10.2] |  | ***nad3*** | 0.3 | [0.8] | 0.8 | [0.0] | 1.1 | [0.8] |  |
| *ndhH* | 6.8 | [5.9] | 7.0 | [6.9] | 8.3 | [6.9] |  | ***rpoC1*** | 8.4 | [8.5] | 6.4 | [7.9] | 10.5 | [11.1] |  | ***nad4L*** | 0.3 | [1.0] | 0.7 | [1.0] | 0.3 | [0.0] |  |
| *ndhI* | 7.4 | [5.6] | 8.0 | [9.3] | 9.7 | [8.7] |  | ***rpoC2*** | 14.9 | [20.6] | 11.2 | [17.2] | 16.8 | [22.8] |  | ***nad4*** | 0.8 | [1.2] | 0.3 | [0.2] | 0.7 | [1.4] |  |
| *ndhJ* | 7.1 | [7.0] | 6.3 | [5.7] | 8.6 | [8.9] |  | ***rps11*** | 7.0 | [5.8] | 8.6 | [8.7] | 8.4 | [6.5] |  | ***nad5*** | 0.5 | [0.9] | 0.4 | [0.6] | 0.8 | [1.3] |  |
| *ndhK* | 10.0 | [13.5] | 6.7 | [6.6] | 9.7 | [9.8] |  | ***rps12*** | 5.3 | [2.4] | 5.6 | [2.4] | 5.6 | [1.6] |  | ***nad6*** | 1.0 | [1.0] | 1.3 | [2.0] | 1.6 | [2.0] |  |
| *petA* | 8.4 | [6.9] | 6.6 | [5.3] | 9.8 | [9.4] |  | ***rps14*** | 5.6 | [7.0] | 5.3 | [5.0] | 6.3 | [7.0] |  | ***nad7*** | 0.7 | [1.0] | 0.6 | [1.3] | 0.8 | [1.3] |  |
| *petB* | 7.7 | [3.3] | 4.3 | [1.9] | 7.1 | [1.4] |  | ***rps15*** | 8.4 | [13.3] | 12.1 | [23.3] | 12.8 | [17.8] |  | ***nad9*** | 1.2 | [1.6] | 0.9 | [1.1] | 0.7 | [0.5] |  |
| *petD* | 6.4 | [3.8] | 6.2 | [1.2] | 7.5 | [3.1] |  | ***rps16*** | 7.4 | [8.8] | - | - | - | - |  | ***rpl2*** | - | - | - | - | - | - |  |
| *petG* | 3.5 | [2.7] | 3.5 | [5.4] | 3.5 | [2.7] |  | ***rps18*** | 7.9 | [12.5] | 9.4 | [15.7] | 11.7 | [17.5] |  | ***rpl5*** | 1.4 | [2.7] | 1.2 | [2.2] | 1.1 | [2.7] |  |
| *petL* | 11.5 | [12.9] | 10.4 | [12.9] | 15.6 | [19.4] |  | ***rps19*** | 7.5 | [9.8] | 5.0 | [6.5] | 8.6 | [9.8] |  | ***rpl10*** | - | - | - | - | - | - |  |
| *petN* | 3.3 | [3.4] | 2.2 | [3.4] | 5.6 | [6.9] |  | ***rps2*** | 8.3 | [6.8] | 5.8 | [8.5] | 10.5 | [12.3] |  | ***rpl16*** | 0.5 | [0.5] | 1.4 | [2.7] | 1.8 | [2.2] |  |
| *psaA* | 6.6 | [3.3] | 5.5 | [2.7] | 7.3 | [3.5] |  | ***rps3*** | 7.2 | [7.8] | 8.4 | [9.7] | 9.5 | [12.5] |  | ***rps1*** | - | - | 5.3 | [9.6] | - | - |  |
| *psaB* | 5.8 | [2.3] | 5.1 | [2.5] | 7.1 | [3.3] |  | ***rps4*** | 10.9 | [13.4] | 7.6 | [10.0] | 10.7 | [11.9] |  | ***rps2*** | - | - | - | - | - | - |  |
| *psaC* | 5.3 | [1.2] | 2.0 | [1.2] | 4.5 | [0.0] |  | ***rps7*** | 2.6 | [1.3] | 2.4 | [2.6] | 2.4 | [2.6] |  | ***rps3*** | 2.3 | [4.2] | 2.6 | [4.2] | 2.5 | [4.1] |  |
| *psaI* | 7.6 | [17.6] | 6.7 | [8.8] | 8.6 | [14.7] |  | ***rps8*** | 8.9 | [11.2] | 9.6 | [11.2] | 11.1 | [14.2] |  | ***rps4*** | 1.7 | [3.4] | 2.0 | [5.5] | 2.8 | [6.1] |  |
| *psaJ* | 8.1 | [4.5] | 5.9 | [0.0] | 9.6 | [4.5] |  | ***ycf1*** | 19.9 | [35.4] | 23.6 | [38.4] | 26.9 | [43.8] |  | ***rps7*** | - | - | - | - | - | - |  |
| *psbA* | 5.1 | [1.1] | 4.1 | [1.1] | 5.5 | [0.8] |  | ***ycf2*** | 6.5 | [10.7] | 6.6 | [11.5] | 6.7 | [12.0] |  | ***rps10*** | 2.7 | [8.2] | 1.5 | [4.5] | 3.0 | [5.5] |  |
| *psbB* | 5.8 | [2.6] | 5.6 | [1.6] | 7.3 | [2.8] |  | ***ycf3*** | 6.3 | [4.8] | 4.3 | [2.4] | 7.5 | [5.4] |  | ***rps11*** | - | - | - | - | - | - |  |
| *psbC* | 6.0 | [1.1] | 5.0 | [1.5] | 6.5 | [1.3] |  | ***ycf4*** | 34.6 | [57.4] | 23.5 | [41.8] | 34.6 | [55.0] |  | ***rps12*** | 0.5 | [0.0] | 0.5 | [0.8] | 1.1 | [0.8] |  |
| *psbD* | 4.1 | [0.0] | 3.7 | [0.0] | 4.9 | [0.0] |  | ***-*** | - | - | - | - | - | - |  | ***rps13*** | - | - | - | - | - | - |  |
| *psbE* | 4.4 | [1.2] | 4.4 | [2.4] | 4.8 | [1.2] |  | ***-*** | - | - | - | - | - | - |  | ***rps14*** | 0.7 | [2.0] | 1.0 | [3.0] | 0.3 | [1.0] |  |
| *psbF* | 5.0 | [2.6] | 6.7 | [2.6] | 4.2 | [5.1] |  | **-** | - | - | - | - | - | - |  | ***rps19*** | - | - | - | - | - | - |  |
| *psbH* | 10.8 | [12.3] | 6.8 | [9.6] | 9.9 | [12.3] |  | **-** | - | - | - | - | - | - |  | ***sdh3*** | - | - | - | - | - | - |  |
| *psbI* | 9.9 | [0.0] | 7.2 | [0.0] | 13.5 | [0.0] |  | **-** | - | - | - | - | - | - |  | ***sdh4*** | - | - | - | - | - | - |  |
|  | | | | | | | |  | | | | | | | |  | | | | | | | |
| Average: | | | | | | | |  | 7.99 | [8.42] | 7.49 | [8.47] | 9.62 | [10.09] | |  | 1.60 | [2.26] | 1.29 | [2.30] | 1.87 | [2.63**]** | |

Note: The *V. radiata* cpDNA contains the *rps16* and *rpl33* pseudogenes. All three cpDNA also contain the *ycf15* and *ycf68* pseudogenes as well as partial copies of the *ycf1* and *ycf2* genes. The *infA*, *sprA*, *rpl21* and *rpl22* genes are also notably absent from all three cpDNA. The mitochondrial genes, *cox2* and *rps1* are absent from *V. radiata* and *L. japonicus* respectively. Also, the *sdh3* gene is intact in the Pongamia mtDNA.
